# Antidiabetic Activity and Inhibitory Effects of Derivatives of Advanced Aminoguanidine Glycation

**DOI:** 10.1021/acsomega.5c01940

**Published:** 2025-10-07

**Authors:** Patrícia de Albuquerque Sarmento, Andressa Letícia Lopes da Silva, Edeildo Ferreira da Silva-Júnior, Elita Scio, Danielle Maria de Oliveira Aragão, Êurica Adélia Nogueira Ribeiro, Érica Erlanny da Silva Rodrigues, Pedro Gregório Vieira Aquino, Bárbara Viviana de Oliveira Santos, Antônio Euzébio Goulart Santana, Aline Cavalcanti de Queiroz, Magna Suzana Alexandre-Moreira, Henrique Douglas Melo Coutinho, João Xavier de Araújo Júnior

**Affiliations:** 1 Wound Treatment Research Laboratory − LPTF/School of Nursing, Federal University of Alagoas, Maceió, AL 57072-970, Brazil; 2 Pharmacology and Immunity Laboratory-LAFI/Institute of Biological and Health Sciences, Federal University of Alagoas, Maceió, AL 57072-970, Brazil; 3 Biological and Molecular Chemistry Research Group (GPQBioMol), Institute of Chemistry and Biotechnology, Federal University of Alagoas, Maceió, AL 57072-970, Brazil; 4 Laboratory of Bioactive Natural Products - LPNB Institute of Biological Sciences, 28113 Federal University of Juiz de Fora, Juiz de Fora, MG 36036-900, Brazil; 5 Cardiovascular Pharmacology Laboratory (LFC)/ Institute of Pharmaceutical Sciences (ICF), Federal University of Alagoas, Maceió, AL 57072-970, Brazil; 6 Medicinal Chemistry Laboratory (LQM)/Institute of Pharmaceutical Sciences (ICF), Federal University of Alagoas, Maceió, AL 57072-970, Brazil; 7 Research Group on Bioactive Substances of Natural and Synthetic Origin, Federal University of Agreste de Pernambuco, Garanhuns, PE 55292-270, Brazil; 8 Teacher Training Center, UACEN, Federal University of Campina Grande, Campina Grande, PB 58429-900, Brazil; 9 Natural Products Research Laboratory, Federal University of Alagoas, Maceió, AL 57072-970, Brazil; 10 Laboratory of Microbiology and Molecular Biology (LMBM), Regional University of Cariri, Crato, CE 63105-000, Brazil

## Abstract

Aminoguanidine is a drug that prevents the formation of AGEs by reacting with initial glycation products and is effective in improving proteinuria and vessel elasticity, preventing diabetic retinopathy, and treating patients with diabetic nephropathy. Structural modifications of this molecule were carried out, and 19 derivatives were studied to present a potential hypoglycemic and antiglicante effect, preventing such complications for diabetics. For this purpose, an in vitro cytotoxicity test was initially carried out by colorimetric assay with MTT [3-(4,5-dimethylthiazol-2-yl)-2,5 diphenyltetrazolium], in macrophages of the J774 lineage. The AGEs were produced in vitro from the junction of glucose with bovine serum albumin and tested for evaluation of the antiglicante activity with reading in a fluorescence spectrophotometer. Derivatives with the best in vitro response were submitted to in vivo acute toxicity tests with Wistar rats and later evaluation of their antidiabetic and antiglycant potential in rats with streptozotocin-induced diabetes. After euthanasia, the heart, kidney, liver, and pancreas were removed for histopathological examination, and the blood for blood count and glycated hemoglobin, glucose, insulin, triglycerides, total cholesterol, HDL cholesterol, serum albumin, fructosamine, TGO, TGP, and GGT were collected. It is possible to conclude that the studied derivatives have the potential for the production of a drug that can be produced with them or associated with them and that is capable of reducing the glycemic indices and, at the same time, having an antiglicant action protecting individuals from macro- and microvascular complications.

## Graphical abstract

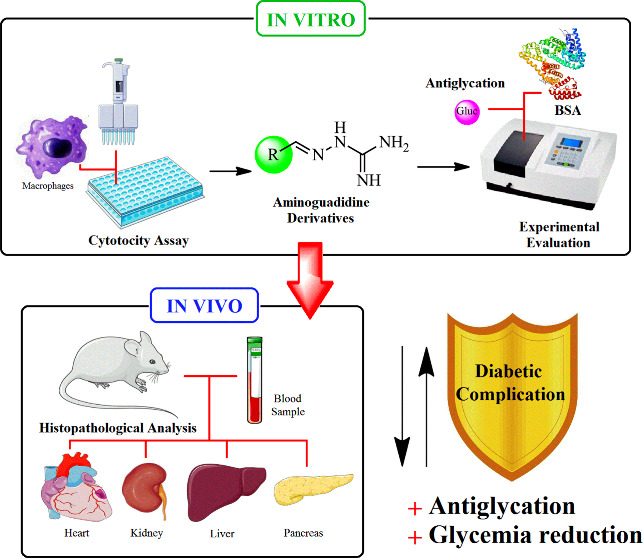

## Introduction

Diabetes Mellitus is a group of metabolic diseases characterized by chronic hyperglycemia resulting from defects in insulin secretion or action, or both.^1^ In the long term, affected individuals have a higher risk of complications, which involve microangiopathies, such as retinopathy, nephropathy and diabetic neuropathy, and macroangiopathy, which is arteriosclerosis of the large arteries: cerebral, coronary and lower limb arteries, leading to renal failure, blindness and amputations.^2^

Diabetes affects approximately 346 million people worldwide, making it a global epidemic.^1^ In Brazil, an estimated six million people have diabetes, with the association between diabetes and hypertension being the main causes of hospitalizations in the country’s public health system.^3^

It is estimated that deaths from diabetes will double between 2005 and 2030. More than 80% of deaths from diabetes occur in low- and middle-income countries.^1^ Unlike developed countries, where diabetes primarily affects older age groups, in developing countries, individuals aged 35 to 64 are the most affected.^3^ In Brazil, mortality by age and gender in individuals with diabetes was 57% higher than in the general population.^4^ And despite government campaigns and programs to detect the disease, there is still little knowledge of the population about the diagnosis before the onset of complications.^4^

Among the theories that explain cell and tissue damage that lead to disease complications, Advanced Glycation End Products (AGEs) formation and accumulation is considered one of the most important. AGEs are a group of heterogeneous molecules produced by biochemical reactions formed from the nonenzymatic glycation of proteins, lipids and nucleic acids.^5^

The diet is considered the main exogenous source of AGEs, processed foods that undergo heat treatment, in addition to grilled and fried foods, have high levels of AGEs. There is evidence that these add to endogenous AGEs, favoring the emergence and progression of the various complications of diabetes.^6^

The pathological effects of AGEs are related to the ability of these compounds to modify the chemical and functional properties of the most diverse biological structures. Through the generation of free radicals, the formation of cross-links with proteins or interactions with cell receptors. The interaction of AGEs with their receptor (RAGE) can activate complex signaling pathways that cause oxidative stress, morphofunctional changes and increased expression of inflammatory mediators.^6^

Agents with anti-AGE properties are currently being investigated. Investigated drugs include aminoguanidine, aspirin, OBP 9195, ALT-946, alagebriun also known as ALT-711, metformin and angiotensin-II receptor blockers which, although none of them have yet been approved for specific indications as anti-AGEs, some are already in preclinical and clinical testing phases.^7^

Aminoguanidine is considered one such anti-AGE agent. It is being tested in humans, as it prevents the formation of AGEs by reacting with the initial products of glycation and is effective in improving proteinuria, vessel elasticity, preventing diabetic retinopathy and treating patients with diabetic nephropathy. However, some side effects are associated with its chronic use and include a higher incidence of glomerulonephritis and vitamin B6 deficiency, which has required research to establish a safe dosage for its therapeutic use in patients with diabetes.^8^

The present work used as a starting point the structure of aminoguanidine, which underwent molecular hybridization and bioisosterism processes, as planning strategies for such modifications. Seeking an effective, low-cost and mostly safe therapeutic alternative for the treatment of diabetes and its complications or as a potential inhibitor of advanced glycation end products.

## Methodology

Before conducting the experiments inherent in this research, the project was submitted to the Ethics Committee on the use of animals (CEUA) of the Pro-Rectory of Research at the Federal University of Juiz de Fora and approved under number 029/2013.

### Obtaining the Synthesis Derived from Aminoguanidine

The syntheses used in this study were based on previous works by our research group, including the synthesis and pharmacological evaluation of amidine derivatives.^9,10^ Guanylhydrazones (LQM01-LQM20) were synthesized by condensation of the appropriate aryl aldehyde (1.0 equiv) with aminoguanidine hydrochloride (1.25 equiv) in methanol under reflux^9,10^ (Figure 1). The reaction mixture was stirred overnight, then cooled to room temperature to induce crystallization. The resulting solids were collected by filtration and dried under vacuum. After solvent removal under reduced pressure, the crude solids were triturated with ethyl acetate to afford the desired guanylhydrazones as fine powders. Experimental detail in Supporting Information.

**Figure 1:**
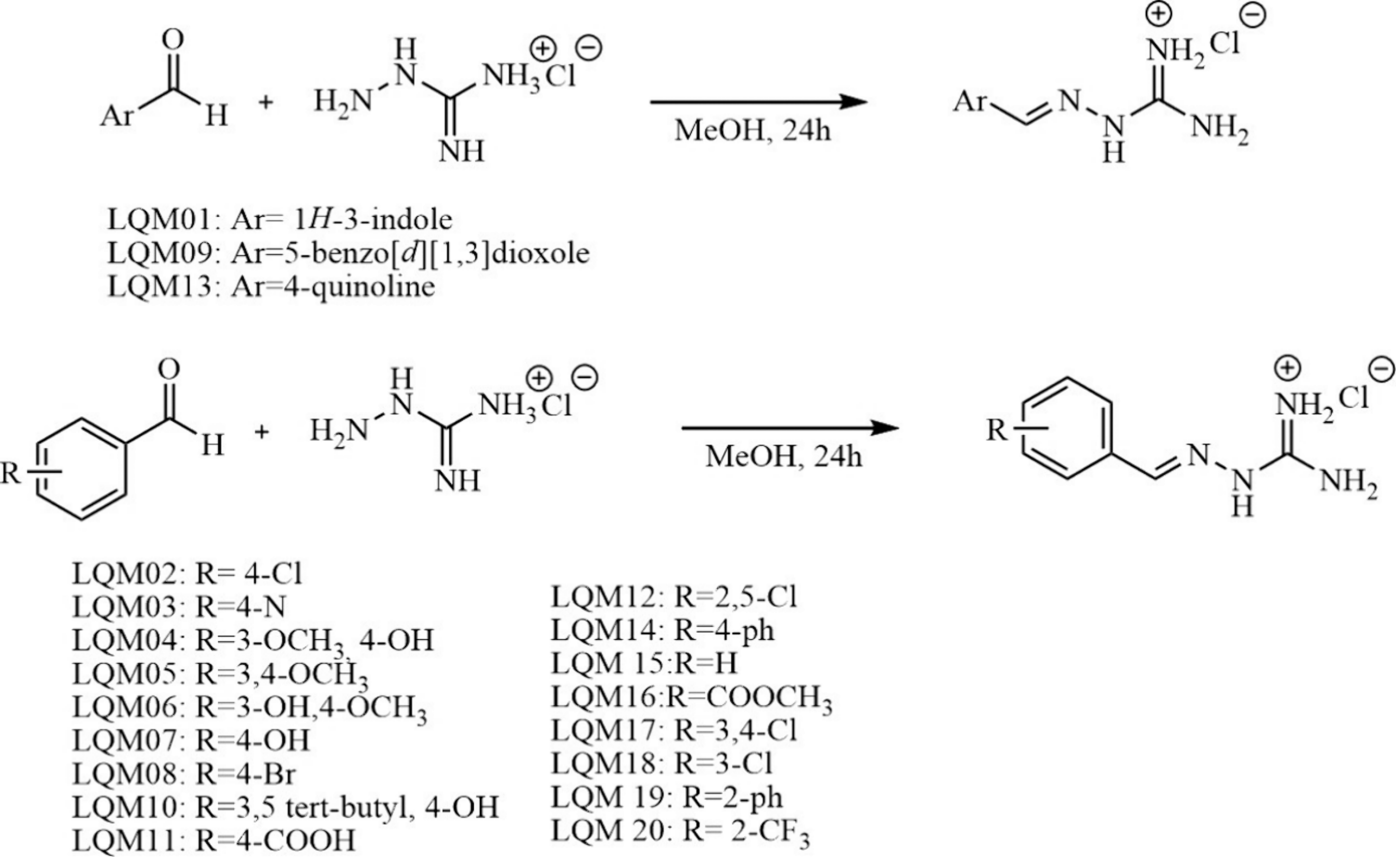
Guanylhydrazone synthesis.

### Cytotoxicity Assessment

To evaluate a possible cytotoxic effect of aminoguanidine derivatives, the colorimetric assay with MTT [3-(4,5-dimethylthiazol-2-yl)-2,5 diphenylterazolium] was used in macrophages of the J774 lineage. These macrophages were maintained in a culture medium containing 10% fetal bovine serum (BSA) and 1% antibiotic, at 37 °C, in an atmosphere of 95% air and 5% CO2.

In this assay, 96-well plates were used, to which 100 μL of a suspension were added at a concentration of 2 × 105 cells/100 μL. Then, these cells were exposed to amnoguanidine derivatives at concentrations ranging from 10 μM, 100 μM and 1000 μM, and subsequently kept in a humidified oven for 48 h.

After this interval, 10 μL of MTT (5 mg/mL) dissolved in 10% BSA was added to each well and incubated at 37 °C for 4 h. The supernatant was then discarded and placed in each culture plate with 150 μL/well of DMSO solution. Cells that have mitochondrial integrity, that is, remain viable, metabolize MTT through the enzyme succinate-dehydrogenase, to a violaceous salt insoluble in aqueous medium and soluble in DMSO called formazam. After 15 min of incubation at room temperature, the resulting solutions were taken to an absorbance reader, measured spectrophotometrically at 540 nm. Five individual wells per treatment were analyzed, and percentage viability was determined relative to controls [(absorbance of treated cells/absorbance of untreated cells) × 100].^11^

### Determination of In Vitro Antiglycant Activity

Performing the test to determine the antiglycant potential of a substance consists of producing the AGE, from the addition of BSA plus a sugar that can be ribose, fructose or glucose.

Preceding the tests with aminoguanidine derivatives, some studies were necessary to standardize the technique, considering that the literature presents several ways of producing AGEs in vitro.^12^

Tests for in vitro antiglycant activity were performed in sterile 96-well plates. Before starting the tests, all materials, including all solutions, buffer and samples, were subjected to ultraviolet light for 15 min in a laminar flow hood, a place where the plate was prepared.

All reagents and samples were sterilized by filtration through 0.2 μm membrane filters to perform the test. The following solutions were added to each well in this order:

- 75 μL of sodium azide 8 g/L;
- 37.5 μL of 800 mM glucose;
- 37.5 μL of 800 mM fructose;
- 75 μL Sample;
- 75 μL of BSA 40 mg/mL.

Having as a variation the blank that contained:

- 150 μL of 100 mM sodium phosphate buffer, pH 7.4;
- 75 μL of sodium azide 8 g/L
- 75 μL of BSA 40 mg/mL.

The negative fluorescence control was used

- 125 μL of 100 mM phosphate buffer, pH 7.4
- 75 μL of sodium azide 8g/L

And the control of the technique that follows the pattern without the sample being analyzed. As positive controls, amnoguanidine and quercetin at 2 mg/mL were used, as well as the tested samples, which were the 19 amnoguanidine derivatives.

The prepared plates were immediately placed in an oven at 37 °C, covered with aluminum foil, and incubated for 48 h. They were not manipulated until the reading was performed. A fluorescence spectrophotometer measured the formation of AGEs, with excitation at 350 nm and emission at 450 nm.^12^

The results were presented in percentage of inhibition and calculated from the following equation:

%inhibition=[1−(solutionfluorescencewithinhibitors/solutionfluorescencewithoutinhibitors)]x100%

### Acute Dose Toxicity Study

The experimental protocol of this research was approved by the Research Bioethics Committee of the Federal University of Juiz de Fora, through opinion n° 029/2013.

Acute toxicity studies are those used to assess the toxicity produced by a test substance when administered in one or more doses over a period not exceeding 24 h, followed by observation of the animals for 14 days after administration.

Acute toxicity was performed with derivatives LQM03, LQM013 and LQM015 at concentrations of 2 mg/kg, 10 mg/kg and 100 mg/kg of animal weight, being treated orally (gavage) with a single dose. This test was conducted following the guidelines of the Guide for conducting nonclinical studies of toxicology and pharmacological safety, necessary for developing medicines, of the National Health Surveillance Agency - ANVISA, of January 31, 2013.

The animals were separated into ten groups according to Table 1, with six animals each. Each animal received 1 mL of the sample, at the specific concentration of its group, solubilized in Twen 80, which is inert and does not cause changes in the active principle to be tested, and the negative control received only H2O.

**Table 1:** Groups for Acute Toxicity Test, Treatments with Concentration, and Number of Wistar Rats Per Group

group	treatment	N
negative control	water	6
LQM03/2	derivative LQM03 with 2 mg/kg of weight	6
LQM03/10	derivative LQM03 with 10 mg/kg of weight	6
LQM03/100	derivative LQM03 with 100 mg/kg weight	6
LQM13/2	derivative LQM13 with 2 mg/kg of weight	6
LQM13/10	derivative LQM13 with 10 mg/kg of weight	6
LQM13/100	derivative LQM13 with 100 mg/kg of weight	6
LQM15/2	derivative LQM15 with 2 mg/kg of weight	6
LQM15/10	derivative LQM15 with 10 mg/kg of weight	6
LQM15/100	derivative LQM15 with 100 mg/kg of weight	6
	total of animals	60

On the first day, the animals were monitored for signs of general toxicity and mortality, at 30, 60, 120, 180, and 240 min after administration of the single dose.^13^

The clinical evaluation observed the following parameters: changes in the skin, hair, eyes, mucous membranes, respiratory, circulatory and central nervous system changes, that is, changes in the conscious state and disposition, in the motor system, in muscle tone, in the central nervous system (remors, convulsions and sedation) and in the autonomic nervous system (tearing, salivation and breathing). In the subsequent 14 days, in addition to these parameters, the animals were monitored by observing the variation in the animal’s body mass and water and feed consumption.

The monitoring of water and feed consumption was carried out through daily measurements, with their respective records in animal consumption sheets. Feed consumption was calculated considering the feed provided, and the leftover feed in the feeders, and then weighed. The same was done to record daily water consumption, using volumetrically graduated individual drinkers.

After this period, euthanasia was performed for histological observation of the kidney, liver and heart, in addition to a complete blood count and biochemical parameters for evaluating lipid and protein profiles and hepatic and nephrotoxicity.

### Animals and Experimental Groups

The in vivo assays were carried out respecting the Ethical Principles in Animal Experimentation, with rats (Rattus novergicus Albinus - Wistar lineage), adult, males, aged two months, acquired at the Central Animal Facility of the UFJF and transferred to the laboratory’s sectorial vivarium. of Natural Resources. The animals were observed for 15 days before the test, to verify the clinical conditions and identify variables that could influence the experiment.

The animals were housed on appropriate shelves, kept in polypropylene cages, one cage per group, lined with sawdust, in a photoperiod of 12 h of light and dark, minimal noise, room temperature 21 ± 1 °C, maintained by air conditioning. Food and water intake were monitored daily. Commercial feed was used (standard feed for rodents - Nuvital, Nuvilab, Brazil).

After weighing, they were separated by the probabilistic Random selection method into 11 groups, identified based on the therapy, as shown in Table 2.

**Table 2:** Experimental Groups, Diabetes Induction, Treatments with Concentration, and Number of Wistar Rats per Group

group	diabetes	treatment	N
GLI	yes	glimepiride	6
CND	yes	water	6
amino D	yes	aminoguanidine 2 mg/kg of weight	6
LQM03D	yes	derivative LQM03 with 2 mg/mL	5
LQM13D	yes	derivative LQM13 with 2 mg/mL	5
LQM15D	yes	LQM15 derivative with 2 mg/mL	5
CNN	no	water	6
amino N	no	aminoguanidine 2 mg/kg of weight	6
LQM03N	no	derivative LQM03 with 2 mg/mL	5
LQM13N	no	derivative LQM13 with 2 mg/mL	5
LQM15N	no	LQM15 derivative with 2 mg/mL	5
		total of animals	60

### Diabetes Induction

After marking the animals (as recommended by the Brazilian Collegiate for Animal Experimentation (COBEA), the animals were weighed individually, to determine the calculation of streptozotocin to be injected in each animal. Therefore, with results were expressed in mg and transformed into g for weighing on the scale.

Streptozotocin concentration = 20 mg/kg

20 mg ------------------- 1000g

X ----------------------- Mean animal weight

X = Average animal weight x 40

--------------------------------------

1000

The induction of the diabetogenic condition occurred through an intravenous injection in the caudal vein of streptozotocin (20 mg/kg) diluted in 120 μL 0.1 M citrate buffer, pH 4.5, in rats previously submitted to a 16-h fast. The animals used as negative controls for the induction (normal animals) received only the 0.1 M citrate buffer, pH 4.5. Seven days after induction, fasting blood glucose was measured to confirm the diabetogenic condition. Streptozotocin was chosen because it is a chemical agent with specific cytotoxicity for beta cells. This drug causes primary insulin insufficiency of the pancreas, causing a triphasic response in glycemic levels during the first hours of administration, followed by the establishment of permanent diabetes in the subsequent 24 h.^14^

Animals that received streptozotocin during induction and presented fasting glycemia above 150 mg/dl were used in the study. Glycemia dosages were performed in the caudal vein and an 8-h fast was maintained.

### Treatment and Euthanasia

For 14 days the animals were treated with the respective treatments, through gavage and monitored for behavior, body weight variation, food intake, and water consumption. During treatment, two animals died, one from the normal LQM 13 group and the other from the diabetic LQM13 group. It cannot be said that it was due to the ingestion of the derivative. As the gavage is risky, a route error may have occurred during administration.

After this period, the animals were anesthetized with an intraperitoneal injection of an association between Xylasine (10 mg/kg) and Ketamine (90 mg/kg) and euthanized by exsanguination with thoracotomy and cardiac puncture to collect 4 to 5 mL of blood for biochemical analyzes of hemogram, glycated hemoglobin, fructosamine, for evaluation of antiglicante activity in vivo and other exams for verification of renal and hepatic function and cholesterol levels.

### Histological Evaluation

Vital organs (heart, pancreas, kidney and liver) were collected, weighed and evaluated macroscopically. To calculate the mass of these organs, the relative value was considered, according to the following equation:

-
Relativemass=organmass/bodymass×100

Afterward, they were placed in formaldehyde 10% (v/v) to prepare histological slides. The fragments were processed according to routine techniques for inclusion in paraffin blocks, cut in a microtome at 3–4 μm and stained using the Hematoxylin-Eosin (H.E.) technique. A pathologist, a professor at the Federal University of Alagoas, regarding the presence or absence of histopathological alterations evaluated the organs described above.

### Statistical Analysis

To aid the statistical analysis, the GraphPad Prism 5.0 software was used. Results were expressed as mean + standard deviation or mean + standard error. Differences were determined by analysis of variance (ANOVA) and Dunnett’s post-test. Results were considered statistically significant when *p* < 0.05.

## Results and Discussion

The observed evaluation parameters are the percentage of cell death, that is, it verifies when a substance had an action on cell viability of less than 80%,^15^ being considered toxic in comparison with the medium that presented 100% viability cell phone. In addition, with this test, the IC_50_ can be calculated, that is, the product concentration necessary to inhibit 50% of cell growth.^16^

The MTT assay results indicated that J774 cells treated with aminoguanidine derivatives were safe and showed no toxic effects in 17 of the 19 derivatives at the lowest experimental concentration of 10 μM.

It is also observed that by increasing this concentration to 100 μM of the sample, ten derivatives remained within the viability standard adopted here, which was 80% of viable cells. Therefore, it can be stated that the cytotoxic evaluation of the compounds on the cells is dose-dependent, that is, the higher the concentration of the derivatives, the greater the toxicity, with lower cell viability, as can be seen in Figure 2.

**Figure 2:**
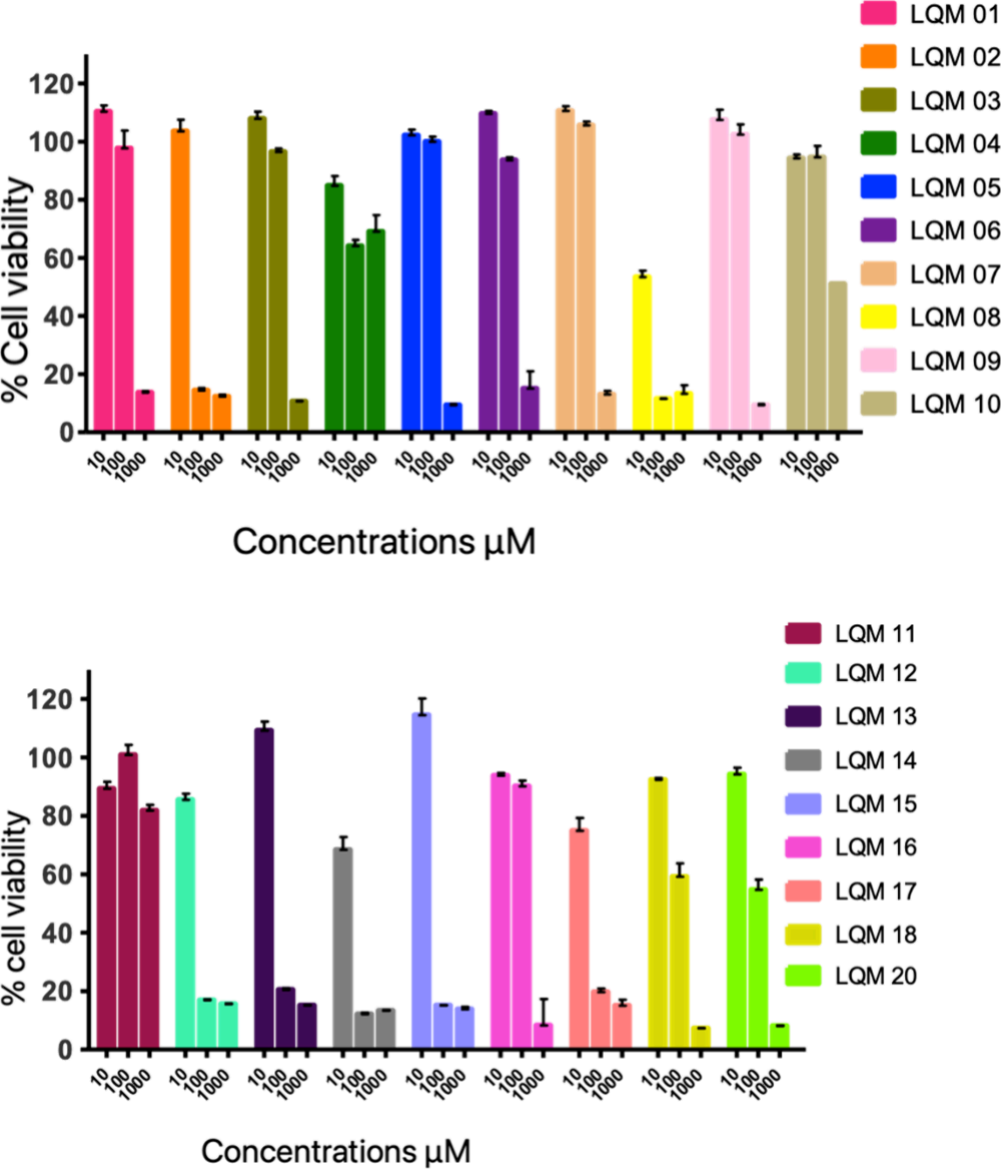
Evaluation of aminoguanidine derivatives at concentrations of 10, 100, and 1000 μM, in the reduction of the percentage of cell viability in macrophages of the J774 lineage, by the colorimetric assay with MTT. In figure A from the derivative LQM 01 to LQM 10 and in figure B from the derivative LQM 11 to LQM 20 The results represent the mean ± standard error of three replicates.

This result suggests that the cytotoxic effects of aminoguanidine derivatives are concentration-dependent, highlighting the need for further studies on their pharmacokinetics and pharmacodynamics.

### Evaluation of In Vitro Antiglicant Activity

The system of employing glucose and BSA for in vitro nonenzymatic glycation studies is a widely used model. Proteins can be modified when exposed to reducing sugars through spontaneous glycation.^17^ This study adopted the BSA-glucose model to select aminoguanidine derivatives with antiglycant potential.

Aminoguanidine and quercetin were standards used as positive controls to inhibit the formation of AGEs (Figure 3). Both inhibited, respectively, 73.61% and 82.43% of AGEs. Therefore, we obtained promising results with seven aminoguanidine derivatives. LQM3, LQM5, LQM6, LQM7, LQM13, LQM16 and LQM17 showed no statistical difference when compared to aminoguanidine. The derivative LQM13 showed no statistical difference when compared with quercetin, considering *p* < 0.05.

**Figure 3:**
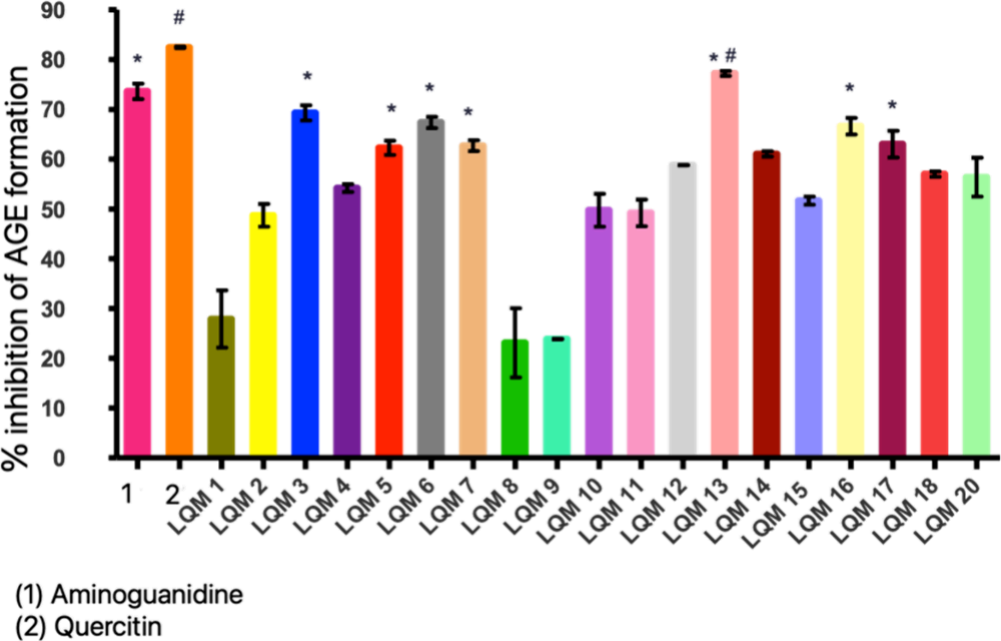
Inhibition of the formation of AGEs formed from glucose–BSA, by aminoguanidine derivatives and their controls at a concentration of 2.0 mg/mL with a 48 h reading. Notes: triplicate averages. * There was no statistically significant difference when compared to aminoguanidine, # There was no statistically significant difference when compared to quercetin after ANOVA with Dunnett’s post-test considering *p* < 0.05.

Furthermore, it was observed that the derivatives LQM4, LQM12, LQM14, LQM15, LQM18 and LQM19 could inhibit more than 50% of the formation, also showing a strong potential to be an anti-AGE. Notably, these results refer to tests at a concentration of 2 mg/mL with a 48-h spectrofluorimetric reading.

Similar results were found in studies with microalgae extracts, whose fractions of ethyl acetate from Chlorella and Nitzschia laevis showed inhibition percentages of 88.02% and 91.68%. This antiglycant capacity of these microalgae was attributed to phenolic compounds, such as flavonoids, tannins and phenolic acids, which have strong antioxidant capacities due to their redox properties. Such antioxidant effects contribute to the subsequent inhibition of the protein glycation process.^17^

With the results presented, we were able to improve the technique presented,^18^ who in a study with methanolic extract *Origanum majorana L*. achieved inhibition percentages, both with the extract and with the positive control, aminoguanidine, lower than this research. The concentrations used were between 0.1 and 0.5 mg/mL and the highest percentage of inhibition was 64.7% for the methanolic extract and 58.3% for aminoguanidine.

Based on the data regarding cellular cytotoxicity through the MTT assay and the ability to inhibit the formation of advanced glycation end products, derivatives LQM 03 and LQM 13 were selected for in vivo assays. In addition, the derivative LQM 15, despite not being one of the best for antiglycant activity, was included for the following tests because it had already shown antidiabetic potential in a preliminary study carried out by our research group.^19^

### Acute Dose Toxicity Study

All animals started the experiment after 15 days of quarantine. The syntheses tested were LQM3, LQM13 and LQM15, at concentrations of 2 mg, 10 mg and 100 mg/KG of weight. After administration, the animals were observed during the first two hours and after six hours of ingestion. In the first ten minutes after ingesting the LQM 15 derivative, the animals showed lethargy at concentrations of 10 mg and 100 mg. However, they responded to mechanical stimulation, with the effect being more pronounced in those receiving 100 mg. In the other animals, no alterations in their behavior were observed.

During the 14 days that followed, no change in behavior could be attributed to the administration of the derivatives. In addition, the percentage of lethality was zero during the treatment days, indicating a low degree of toxicity for these concentrations.

Systemic toxicity can be identified by the decrease in the body weight of the animals and by changes in the consumption of water and feed, which are important signs for the assessment of the toxicity of a substance, as it provides information about the general state of health of the animals.^20^ In this study, no significant difference was found between the negative control and the other groups that received aminoguanidine derivatives, even the animals that received the LQM15 derivatives at concentrations of 10 and 100 mg.

An adult rat, weighing about 300g, consumes approximately five grams of feed and 10 mL of water per day for each 100g of body weight.^21^ However, consumption varies according to environmental temperature and humidity, health status, sex life and time of day, and rats have nocturnal habits and feed almost always at night. Analyzing Table 3 it can be seen that the animals studied here maintained the same pattern. Small differences can be observed by the common losses in the act of feeding and water intake in the cages.

**Table 3:** Parameters of Body Mass, Feed, and Water Consumption of Wistar Rats (*n* = 6) Treated in a Single Dose with Aminoguanidine Derivatives^a^

	parameters		
groups	body mass (g)	water consumption (mL)	feed consumption (mL)
CN	271.1 ± 11.39	36.55^b^ ± 3.891	22.23^c^ ± 2.787
LQM03/2 mg	247.2^a^ ± 7.073	32.34^b^ ± 3.374	21.40^c^ ± 2.014
LQM03/10 mg	240,3^a^ ± 4.920	32.63^b^ ± 4.682	22.40^c^ ± 2.667
LQM03/100 mg	250.0^a^ ± 5.806	34.17^b^ ± 4.468	22.48^c^ ± 2.971
LQM13/2 mg	255.1^a^ ± 5.698	32.78^b^ ± 5.038	22.31^c^ ± 3.795
LQM13/10 mg	257.5^a^ ± 7.999	32.64^b^ ± 4.218	23.59^c^ ± 3.793
LQM13/100 mg	240.7^a^ ± 10.63	32.22^b^ ± 4.564	21.50^c^ ± 2.553
LQM15/2 mg	261.3^a^ ± 10.98	35.56^b^ ± 5.535	22.55^c^ ± 2.880
LQM15/10 mg	268.6^a^ ± 11.82	36.11^b^ ± 4.267	22.50^c^ ± 2.506
LQM15/100 mg	256.8^a^ ± 8.963	32.35^b^ ± 3.581	22.13^c^ ± 4.451

a Means ± standard deviation. Aminoguanidine derivatives and negative control (CN). Means followed by the same letter do not differ using ANOVA with Dunnett’s post test considering *p* < 0.05.

In this study, no significant difference was found between the negative control and the experimental groups that received aminoguanidine derivatives, even the animals that received LQM15 derivatives at concentrations of 10 and 100 mg. Analyzing Table 3, it can be seen that the animals studied here maintained the same consumption pattern. Small differences can be observed by the common losses in the act of feeding and water intake in the cages.

An adult rat, weighing about 300g, consumes approximately five grams of feed and 10 mL of water for each 100g of body weight per day.^21^ However, consumption varies according to environmental temperature and humidity, health status, sex life and time of day, and rats have nocturnal habits and feed almost always at night.

By standardizing the evaluation of the renal function of Wistar rats (*Rattus norvegicus*) from the vivarium of the Federal University of Juiz de Fora,^22^ found similar results for feed intake and water intake to those of this study.

Studies involving preclinical studies use biochemical, hematological and anatomopathological parameters to assess possible signs of toxicity after exposure to the tested substances (Figure 4 and Table 4). All studies found are unanimous in stating that there are variations in hematological and biochemical values in experimental animals due to the conditions of each vivarium and the type of study carried out.^23^

**Figure 4:**
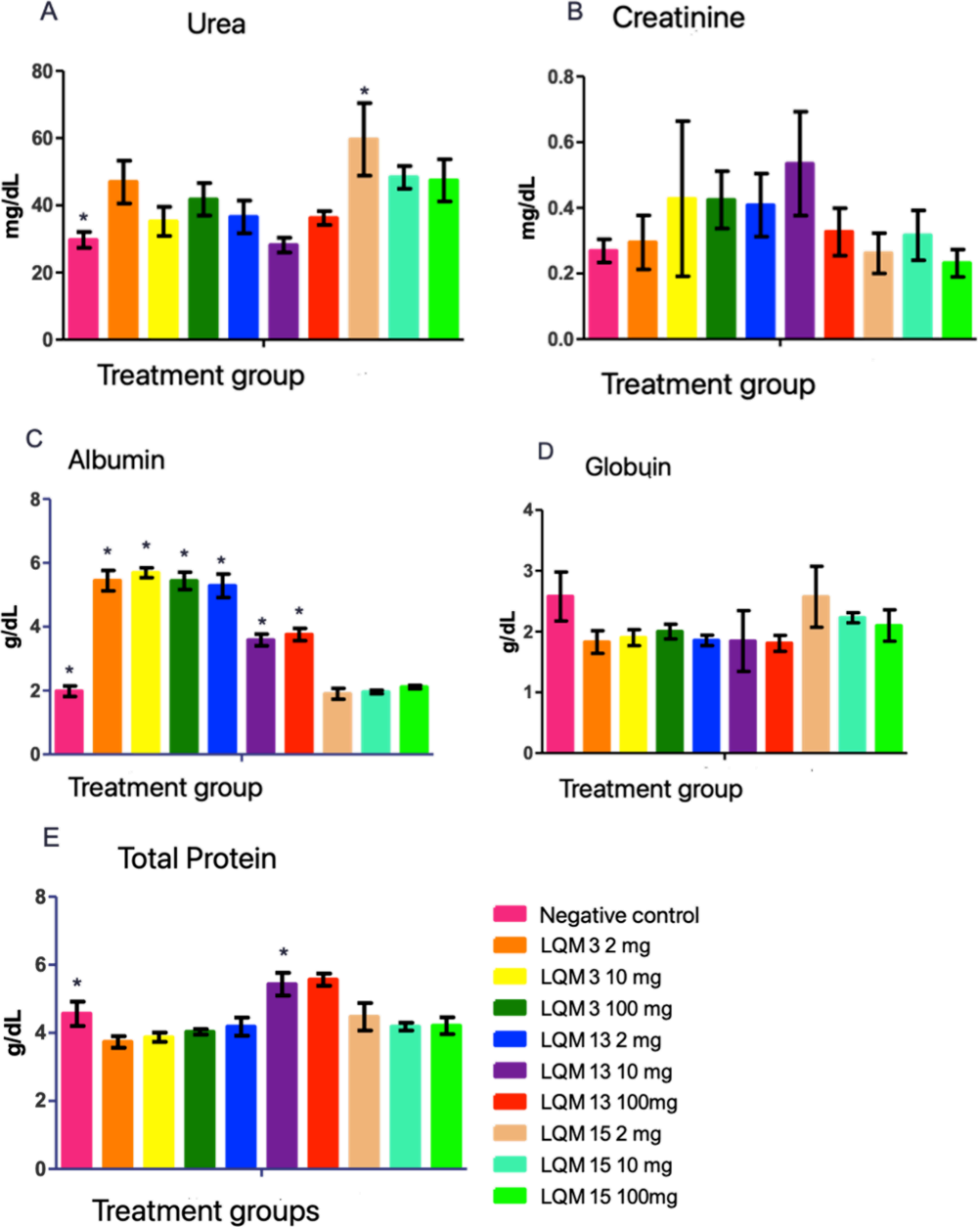
Biochemical values of renal function in Wistar rats from the acute toxicity test treated in a single dose with aminoguanidine derivatives and the negative control (*N* = 6). Notes: Means ± standard error. Aminoguanidine derivatives (LQM) and negative control (CN). Means followed by * differ from CN using ANOVA with Dunnett’s post test considering *p* < 0.05.

**Table 4:** Hematological Parameters and Leucometry Obtained from Wistar Rats (*n* = 6) Treated with a Single Dose of Aminoguanidine Derivatives^a^

			PARAMETerS			
groups	hematocrit (%)	hemoglobin (g/dL)	HCM (pg)	VCM (fL)	CHCM (%)	global leucometry
CN	45.3^a^ ± 1.229	12.9^b^ ± 1.087	24.6^c^ ± 2.157	85.9^d^ ± 2.049	28.53^e^ ± 2.153	3150^f^ ± 328.6
LQM03/2 mg	46.9^a^ ± 0.872	18.7 ± 1.608	29.0^c^ ± 1.029	72.9^d^ ± 2.630	40.03 ± 1.630	3525^f^ ± 231.2
LQM03/10 mg	47.7^a^ ± 0.843	21.0 ± 0.647	30.7 ± 2.245	70.0 ± 2.340	44.32 ± 3.862	3433^f^ ± 470.4
LQM03/100 mg	47.8^a^ ± 0.946	23.0 ± 1.101	34.5 ± 1.860	71.5^d^ ± 2.626	48.30 ± 2.580	4450^f^ ± 636.4
LQM13/2 mg	N/D	12.4^b^ ± 0.378	24.9^c^ ± 0.8310	N/D	N/D	3625^f^ ± 289.5
LQM13/10 mg	N/D	12.0^b^ ± 0.386	23.1^c^ ± 0.5432	N/D	N/D	4300^f^ ± 630.1
LQM13/100 mg	N/D	11.0^b^ ± 0.130	22.0^c^ ± 0.5546	N/D	N/D	3883^f^ ± 517.5
LQM15/2 mg	44.7^a^ ± 1.626	10.4^b^ ± 0.982	22.4^c^ ± 1.696	97.8^d^ ± 6.536	23.10^e^ ± 1.601	2725^f^ ± 318.0
LQM15/10 mg	45.3^a^ ± 1.520	11.0^b^ ± 0.602	22.8^c^ ± 0.555	94.3^d^ ± 4.750	24.42^e^ ± 1.232	2717^f^ ± 431.6
LQM15/100 mg	45.0^a^ ± 1.932	10.7^b^ ± 1.379	23.0^c^ ± 1.754	99.4^d^ ± 3.192	23.48^e^ ± 2.409	2417^f^ ± 297. 7

a Means ± standard error. MCH- mean corpuscular hemoglobin; MCV – mean corpuscular volume; MCHC – mean corpuscular hemoglobin concentration Means followed by the same letter do not differ statistically from each other using ANOVA with Dunnett’s post test considering *p* < 0.05.

The hematological values presented according to the different treatments and concentrations show few changes, being within the reference values, even with a statistically significant difference between some groups. Such alterations did not compromise the organic physiology of the treated animals. Therefore, with the hematological data presented, it was not possible to identify toxicity in the derivatives studied.

The presented results were statistically compared to the negative control, which received only H_2_O, as well as to standard reference values presented in the literature.^24^

An important piece of data to be described about the LQM 13 derivative is that regardless of the concentration administered, that is, 2 mg/kg, 10 mg/kg or 100 mg/kg of the animal’s weight, all blood samples collected clotted immediately, not being possible to establish values for hematocrit, VCM and CHCM, in this case only hemoglobin was calculated. However, the clinical relevance of such findings produced by this derivative needs more extensive studies, so that this potential coagulation capacity can be elucidated.

Global white blood cell counts had low values in all groups. The literature confirms that blood collected from the ventricle or vena cava has lower values when compared with blood collected from the caudal vein. The reason for this difference is not a consensus within the studies.

Dantas^25^ present some reasons for these findings. First, the difference could be attributed to an immunological demand of the animal in the possible places where the blood samples are collected, for example, in the tail, in the eye or in the heart. Another theory is that vascular resistance and blood stasis, generated at the surface of capillary beds, could cause a higher leukocyte count in blood samples collected from the tail. Finally, the stress induced by euthanasia could interfere with the leukocyte count.

Biochemical tests evaluating the kidney and liver function of the animals were also performed and are. The values of cholesterol, triglycerides, LDL, HDL and VLDL were also measured, but there were no pathological changes, as well as a significant difference between the animals.

Regarding renal function, parameters such as urea, creatinine, total protein and fractions (albumin and globulin) were observed. The results indicate that the animals had preserved renal function, regardless of the ingested concentration, even when the values had a statistically significant difference, compared to the negative control, these remained within the reference values described in the literature.^22^

Evaluating the toxicology in Wistar rats of the hydroethanolic extract of Dianthus basuticus, a plant used in traditional Chinese medicine as an antidiabetic,^26^ also did not find alterations in the evaluated parameters of renal function, creatinine, urea, uric acid, as well as sodium, potassium.

The results of this study revealed that there was no increase in the levels of TGO, TGP, GGT and alkaline phosphatase, on the contrary, in some groups the levels were below the negative control, indicating that the ingestion of aminoguanidine derivatives does not appear to cause liver damage. These data corroborate the histopathology of the livers studied.^26^

These enzymes are present in various cells of our body and are present in large amounts in hepatocytes. Therefore, they are considered highly sensitive indicators of hepatocellular damage and, within certain limits, can provide a quantitative rate of the degree of damage suffered by the liver.^26^

Among the morphological evaluation parameters, the weight of the organs heart, liver and kidney did not show macroscopic changes and the mass of the organs did not have a statistically significant difference between the animals that received the derivatives and the negative control.

It was possible to observe that both cardiac (p = 0.5667) and hepatic (p= 0.7072) and renal (p= 0.5474) mass indices do not have a statistically significant difference, corroborating results obtained in other studies, the example from.^20^

In these same organs, histopathological examinations were performed to assess possible morphological alterations that would show some toxicity of the derivatives, as can be seen in Figures 5, 6, and 7. After evaluating the slides, both the control group and those treated with the derivatives did not detected irreversible alterations such as cellular necrosis and apoptosis. These results corroborate the biochemical evaluation, which did not show metabolic alterations that would show nephro and/or hepatotoxicity.

**Figure 5:**
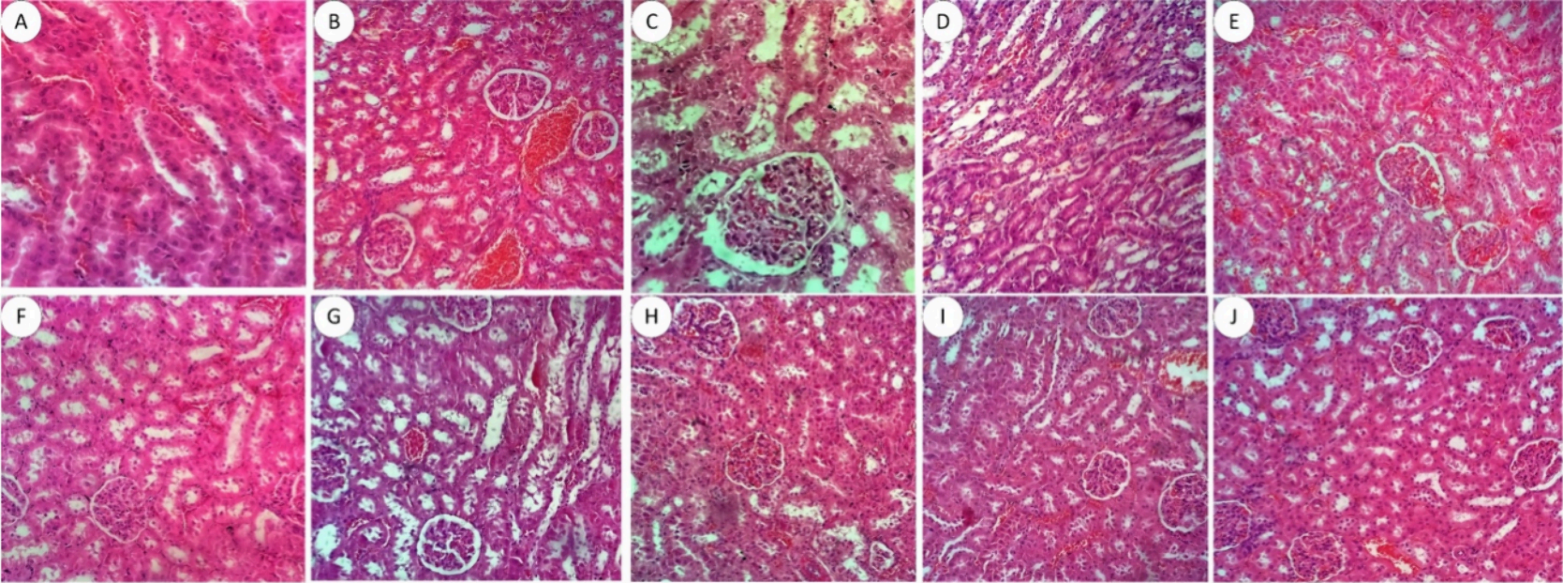
Renal histopathological analysis of the acute toxicity of a single oral dose of aminoguanidine derivatives and the negative control. (A) Negative control; (B) LQM 3 to 2 mg/kg; (C) LQM 3 to 10 mg/kg; (D) LQM 3 at 100 mg/kg; (E) LQM 13 at 2 mg/kg; (F) LQM 13 to 10 mg/kg cells denucleated; (G) LQM 13 at 100 mg/kg; (H) LQM 15 to 2 mg/kg; (I) LQM 15 to 10 mg/kg; (J) LQM 15 to 100 mg/kg. Staining: hematoxylin-eosin. Magnification: 40×. GR – glomerulus.

**Figure 6:**
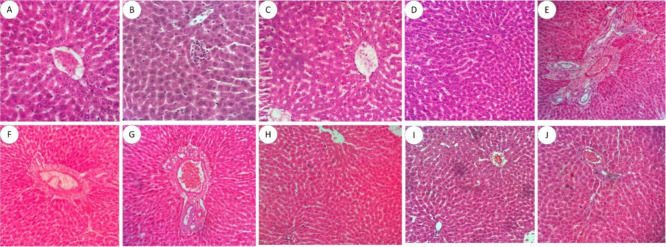
Liver histopathological analysis of the acute toxicity of a single oral dose of aminoguanidine derivatives and the negative control. (A) Negative control; (B) LQM 3 to 2 mg/kg; (C) LQM 3 to 10 mg/kg; (D) LQM 3 at 100 mg/kg; (E) LQM 13 at 2 mg/kg; (F) LQM 13 to 10 mg/kg; (G) LQM 13 at 100 mg/kg; (H) LQM 15 to 2 mg/kg; (I) LQM 15 to 10 mg/kg; (J) LQM 15 to 100 mg/kg. Staining: hematoxylin-eosin. Magnification: 40×. door space; Sinusoids; VP – portal vein; DB – bile duct; AR – artery.

**Figure 7:**
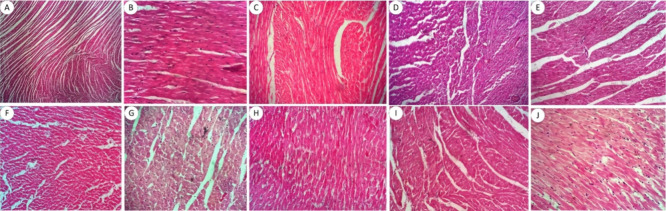
Cardiac histopathological analysis of the acute toxicity of a single oral dose of aminoguanidine derivatives and the negative control. (A) Negative control; (B) LQM 3 to 2 mg/kg; (C) LQM 3 to 10 mg/kg; (D) LQM 3 at 100 mg/kg; (E) LQM 13 at 2 mg/kg; (F) LQM 13 to 10 mg/kg; (G) LQM 13 at 100 mg/kg; (H) LQM 15 to 2 mg/kg; (I) LQM 15 to 10 mg/kg; (J) LQM 15 to 100 mg/kg. Staining: hematoxylin-eosin. Magnification: 40×.

In the kidney (Figure 5), aminoguanidine derivatives, regardless of the administered concentration, did not promote histological changes. The cortical and medullary regions were preserved, the proximal and distal convoluted tubules and the collecting duct segment showed no alterations. There were no foci of inflammation or fibrosis, congestion or areas of hemorrhage, that is, no signs of glomerulonephritis or tubular necrosis. Only non-nucleated cells (Figure 5F) were found in all groups, which may characterize a renal metabolism of derivatives.

In a similar study carried out with the ethanolic extract of *Terminalia chebula*, an Indian plant popularly used as an antidiabetic, the plant, as well as its control, which was aminoguanidine (100 mg/kg), did not show a toxic effect on the kidneys and liver of rats that also had streptozotocin-induced diabetes.^27^

The liver (Figure 6) presents preserved architecture, with morphology and histology described in the literature in mammals, presence of the portal triad, which is composed of a branch of the portal vein, a branch of the hepatic artery and a branch of a bile duct (Figure 6G). Hepatocytes, one of the main agents responsible for metabolizing a series of substances, were unchanged, as well as sinusoidal capillaries (Figure 5 D and I) and Kupffer cells (hepatic macrophages). Hepatic steatosis was not found.

Nico^28^ found similar results when evaluating the action of aminoguanidine on the liver of diabetic rats submitted to physical training. The authors suggest that aminoguanidine is not hepatotoxic at a dose of 1g/L and that its effects are better in individuals submitted to physical training.

For the heart (Figure 7) there were no specific changes visible from the groups treated with the derivatives compared to the negative control. With this type of coloring, the muscle fibers stand out, which remained preserved in all groups.

From the parameters of water and feed consumption, body weight, hematological and biochemical profile, in addition to the anatomopathological analyzes and absence of deaths during the experiment, it can be inferred that in the study for acute toxicity, the derivatives LQM 03, LQM 13 and LQM 15, at concentrations of 2 mg/kg, 10 mg/kg and 100 mg/kg did not demonstrate toxicity.

### Evaluation of In Vivo Antidiabetic and Antiglycant Activity

In the study to evaluate the in vivo antidiabetic and antiglycant potential, the body mass of the animals had little variation during the 14 days of the study, with a slight increase in both normal and diabetic animals. Signs of polyphagia and polydipsia, common symptoms in diabetics, were not observed in the treated diabetic animals during the experimente (Figure 8).

**Figure 8:**
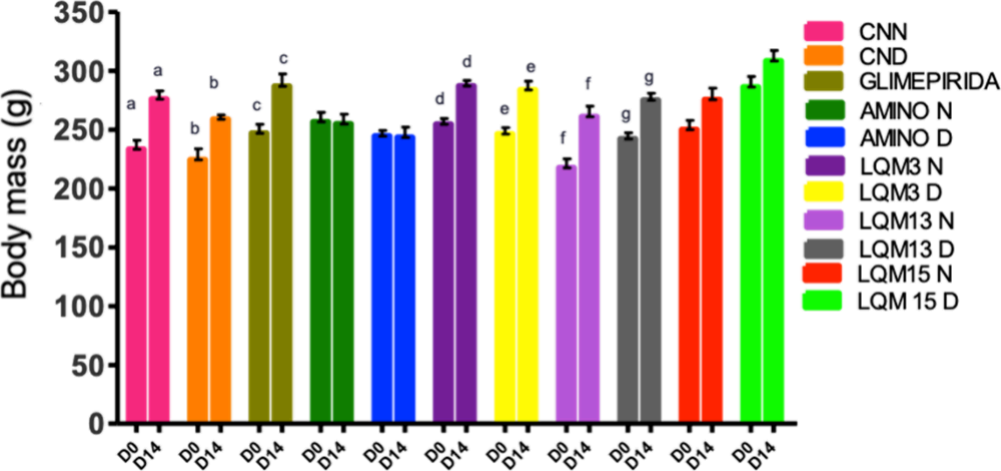
Comparison between the initial (D0) and final (D14) body mass of Wistar rats treated with aminoguanidine derivatives and their respective controls. Notes: Means ± standard error. Equal letters differ statistically from each other. Using Student’s *t* test considering *p* < 0.5.

An important data to be emphasized is that the animals treated with aminoguanidine maintained their weight without statistical difference during the 14 days of the experiment, as well as those that received the LQM 15 derivative did not gain significant weight, both normal and diabetic. This body weight maintenance action of aminoguanidine was not found in the researched studies. Unlike these findings, Majd^29^ found a significant weight gain in the group of diabetic animals treated with a 0.1% (w/v) aminoguanidine solution, ad libitum, for 8 weeks.

Weight loss, which is one of the signs of untreated diabetes, was not observed in any of the diabetic animals in this study. The maintenance of food consumption, with gradual weight gain, may be due to the hypoglycemic effect of derivatives over the days of treatments.

Erejuwa^30^ evaluated the association of honey with glibenclamide and metformin to improve glycemic control, they observed weight loss throughout the experiment in diabetic animals in their study. Neither glibenclamide nor metformin, the standard treatment drugs, prevented this weight loss.

Heart, liver, kidney and pancreatic mass values were calculated considering the animals’ body weight. After 14 days of oral administration of aminoguanidine derivatives to the rats, no alteration in the morphology of the organs was observed in relation to the control groups (Table 5). The differences in mass found are due to the variation in the body weight of the animals in some groups. Histologically, these organs also did not show alterations related to the use of derivatives or their controls (data not shown).

**Table 5:** Morphological Parameters of Rats Treated with Aminoguanidine Derivatives and Their Respective Controls for 14 Days^a^

groups	heart mass	liver mass	kidney mass	pancreatic mass
GLI	0.398 ± 0.018	3.250 ± 0.086	0.380 ± 0.008	0.475 ± 0.045
CND	0.433 ± 0.011	3.320 ± 0.119	0.422 ± 0.015	0.488 ± 0.038
amino D	0.477 ± 0.022	3.507 ± 0.074	0.443 ± 0.008	0.575 ± 0.049
LQM 3D	0.402 ± 0.011	2.958 ± 0.078	0.408 ± 0.010	0.580 ± 0.116
LQM 13D	0.475 ± 0.030	3.205 ± 0.115	0.477 ± 0.017	0.505 ± 0.059
LQM 15D	0.428 ± 0.023	3.182 ± 0.113	0.438 ± 0.029	0.520 ± 0.041
CNN	0.418 ± 0.010	3.442 ± 0.065	0.397 ± 0.016	0.577 ± 0.035
amino N	0.470 ± 0.014	3.377 ± 0.062	0.418 ± 0.018	0.572 ± 0.029
LQM 3N	0.390 ± 0.009	3.096 ± 0.073	0.414 ± 0.015	0.518 ± 0.068
LQM 13N	0.430 ± 0.008	3.165 ± 0.047	0.462 ± 0.005	0.470 ± 0.037
LQM 15N	0.420 ± 0.012	2.978 ± 0.072	0.458 ± 0.020	0.514 ± 0.030

a Means ± standard error. Means followed by the same letter differ statistically using ANOVA with Dunnet’s post test considering *p* < 0.05.

Most studies in rats with streptozotocin-induced diabetes show no changes in organ weights. Eleazu et al.^31^ found no difference for the liver, but the weight of the kidneys was 1.5 times increased. Also Dantas,^25^ evaluating some physiological parameters of rats from the Central Animal Facility of the State University of Maringá, State of Paraná, found an elevated renal mass, even in animals that were not submitted to any drug therapy, nor induction of diabetes.

Mean hematological values according to the different treatments are presented in Table 6. Assessing the hematopoietic system is one of the important parameters used in in vivo studies to determine the physiological and pathological status, as it provides important information about the body’s reaction to an aggression.^32^

**Table 6:** Hematological Parameters and Leukometry Obtained from Diabetic and Nondiabetic Wistar Rats Treated with Aminoguanidine Derivatives and Their Respective Controls^a^

	diabetics groups					
parameters	CND	AD	GLI	LQM 03	LQM 13	LQM15
hematocrit (%)	44.3 ± 1.054^a^	43.8 ± 1.537	44 ± 0.577	38.2 ± 1.068^a^	35.7 ± 0.750^a^	38.2 ± 0.3742^a^
hemoglobin (g/dL)	12.6 ± 0.523	12.4 ± 0.533	13.1 ± 0.894	12.0 ± 1.292	13.1 ± 0.687	13.5 ± 0.728
red blood cell (10^6^)	4.3 ± 0.182	4.6 ± 0.162	4.4 ± 0.295	5.0 ± 0.290	5.0 ± 0.102	4.5 ± 0.282
HCM (pg)	29.1 ± 1.792	27.0 ± 0.907	29.7 ± 1.791	24.8 ± 3.745	25.9 ± 1.298	30.3 ± 3.461
VCM (fL)	102.2 ± 4.787	95.21 ± 0.922	101.4 ± 7.942	77.10 ± 4.432	70.42 ± 2.361	85.12 ± 6.257
CHCM (%)	28.5 ± 0.967^b^	28.44 ± 1.039	29.85 ± 2.238	31.43 ± 3.181	36.9 ± 2.679^b^	35.31 ± 2.117
global leucometry (mm^3^)	3833 ± 636.4	4217 ± 585.0	5483 ± 742.9	3520 ± 554.7	2900 ± 491.2	3080 ± 383.3

	**normal groups**				
**parameters**	**CNN**	**AN**	**LQM 03**	**LQM 13**	**LQM15**
hematocrit (%)	45.2 ± 0.792^c^	44.3 ± 2.108	37 ± 0.8944^c^	39.5 ± 1.658^c^	38 ± 0.707^c^
hemoglobin (g/dL)	12.1 ± 0.733	11.1 ± 0.574	13.7 ± 0.438	13.6 ± 1.281	13.7 ± 0.872
red blood cells (10^6^)	4.9 ± 0.328	4.5 ± 0.313	4.6 ± 0.393	4.6 ± 0.393	4.7 ± 0.061
HCM (pg)	24.7 ± 1.212	25.2 ± 1.829	30.9 ± 3.531	30.7 ± 2.742	29.9 ± 2.020
VCM (fL)	93.75 ± 6.394	99.56 ± 4.694	82.79 ± 7.837	91.79 ± 15.13	80.64 ± 1.379
CHCM (%)	26.67 ± 1.418^d^	25.15 ± 0.856	37.1 ± 0.899^d^	34.7 ± 3.587^d^	36.1 ± 3.587^d^
global leucometry (mm^3^)	6092 ± 940.2^e^	2983 ± 325.2^e^	4590 ± 447.0	2563 ± 379.9^e^	4420 ± 681.3

a Means ± standard error. Means followed by the same letter differ statistically using ANOVA with Dunnet’s post test considering *p* < 0.05.

For a better understanding, the data were grouped into diabetics and nondiabetics. Despite presenting some statistically significant differences when compared to the diabetic negative control and the normal negative control, clinically they are within the parameters reported in the literature.^20^

Anemia was not observed in any group, but leukocytes were low in normal animals that received LQM 13. It is worth remembering that during the experiment there were two deaths in animals that received this derivative, one diabetic and the other normal. And in the acute toxicity study all samples taken from this group showed immediate clotting. It was not possible to correlate these findings to possible adverse effects of this derivative.

Evaluating the in vivo antidiabetic action of the aminoguanidine derivatives LQM 3, LQM 13 and LQM 15 at a concentration of 2 mg/kg of weight, it is possible to state that these three have action to control glycemic values. Statistically, it can be said that these had a similar action to the standard drug glimepiride, which presented a 53% reduction in glucose levels, while LQM3 had 57.6%, LQM13 obtained 57.9% and finally the LQM15 with 65.2% (Table 7).

**Table 7:** Blood Glucose Values in Diabetic Wistar Rats and Percentage Reduction before and after Treatment with Aminoguanidine Derivatives and Their Controls^a^

	fasting blood glucose (mg/dL)		
groups	before treatment	after treatment	% of blood glucose reduction
CNN	91,2 ± 15.50	82.3 ± 10.56	N/A
CND	205.2 ± 26.59	234.8 ± 18.93*	
AN	92.5 ± 15.91	85.8 ± 15.20	N/A
AD	193.2 ± 11.75	106.2 ± 14.69*^a^	45%
GLI	172.7 ± 51.43	81.2 ± 28.46*^a^	53%
W003 N	99.6 ± 9.18	83.4 ± 26.43	N/A
W003 D	196.8 ± 10.92	83.4 ± 8.29*^a^	57.6%
W013 N	95.0 ± 13.39	94.7 ± 19.81	N/A
W013 D	187.0 ± 12.54	78.7 ± 17.46*^a^	57.9%
W015 N	73.2 ± 10.47	97.4 ± 30.42	N/A
W015 D	199.4 ± 18.01	69.4 ± 6.23*^a^	65.2%

a Mean + Standard deviation. Means followed by the same letter in the column do not differ from glimepiride (GLI) and means followed by * in the same column differ from the diabetic negative control (CND) by ANOVA with Dunnett’s post-test considering *p* < 0.05.

Kim,^33^ studying aminoguanidine as a protector against diabetic retinopathy, at a concentration of 50 mg/kg (p.o.) once a day for 13 weeks, also found a small decrease in glycemic values in these rats. It is known that aminoguanidine is not considered an antidiabetic. Its action is to inhibit advanced glycation end products.

Glycated hemoglobin is a monitoring standard for diabetics. Its elevated values reflect the average of blood glucose levels over the last two to four months. Being indicated for chronic studies of diabetes. Despite this concept in humans, Santos^34^ presents high values of glycated hemoglobin in animals with 7 days of diabetes induction by streptozotocin. As the metabolism of Wistar rats is faster than that of humans, it is believed that the hyperglycemia condition quickly induced nonenzymatic glycation reactions.

A1C is a minor component of Hb, being found in nondiabetic adults in a proportion of 1% to 4% of normal individuals. In practice, normal reference values range from 4% to 6%.^35^

Figure 9 shows the glycated hemoglobin values in the diabetic animals in this study. Despite showing a significant difference between the animals treated with the LQM 3 and LQM 13 derivatives and aminoguanidine and the negative control, all animals obtained values below 3%, that is, clinically normal. HBA1c levels above 7% are associated with a progressively greater risk of chronic complications.^35^

**Figure 9:**
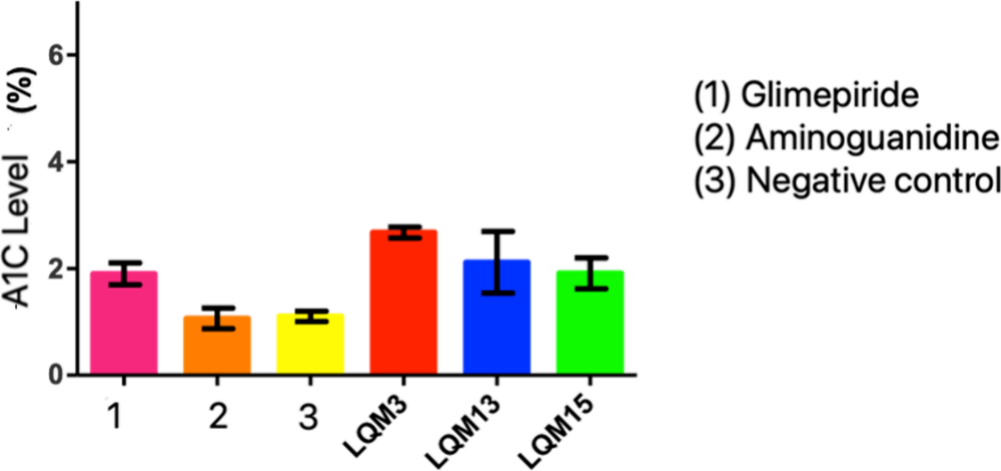
Glycated hemoglobin values of diabetic Wistar rats treated for 14 days with aminoguanidine derivatives and their respective controls. Notes: Means ± standard error. Means followed by *differ from negative control and followed by # differ from aminoguanidine using ANOVA with Dunnett’s post test considering *p* < 0.05.

By using aminoguanidine as an inhibitor of advanced glycation end products, at a concentration of 250 mg/kg, in alloxan-induced diabetic rats, Carvalho^36^ also quantified glycated hemoglobin and fructosamine in these animals. Glycated hemoglobin in this study was 4.8%, that is, higher than the values presented by all groups treated with the derivatives of this research (Figure 10).

**Figure 10:**
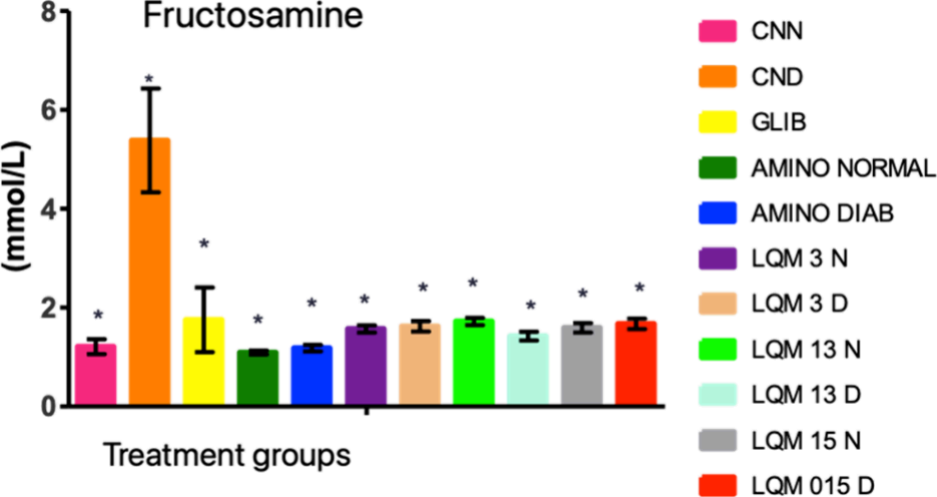
Fructosamine levels in Wistar rats treated for 14 days with aminoguanidine derivatives and their respective controls. Notes: Means ± standard error. Means followed by * differ from each other using ANOVA with Dunnett’s post test considering *p* < 0.05.

In acute studies of glycation, fructosamine is the best parameter because it represents the average concentration of glycation between albumin and glucose in the last two or 3 weeks. It is a nonenzymatic glycation product of serum proteins, mostly albumin, which constitutes the largest plasma protein mass after hemoglobin (50–80%) and the carbonyl group of glucose. It is proportional to the blood glucose concentration and correlates with fasting blood glucose and glycated hemoglobin.^37^

Unlike this study, the concentration of fructosamine was not influenced by the induction of diabetes with streptozotocin in the research by,^38^ when observing its effect on the glycemic and lipid profiles and oxidative stress. Even with STZ increasing glycemic levels by seven times, it was not possible to observe an increase in fructosamine, possibly due to the short duration of the experiment (10 days).

Other biochemical parameters were evaluated, seeking possible effects of treatment with aminoguanidine derivatives on kidney and liver function, in addition to the lipid profile of the animals. Knowing the possible nephrotoxic effects, urea and creatinine were evaluated, in addition to total proteins and fractions that may also be related to hepatotoxicity (Figure 11).

**Figure 11:**
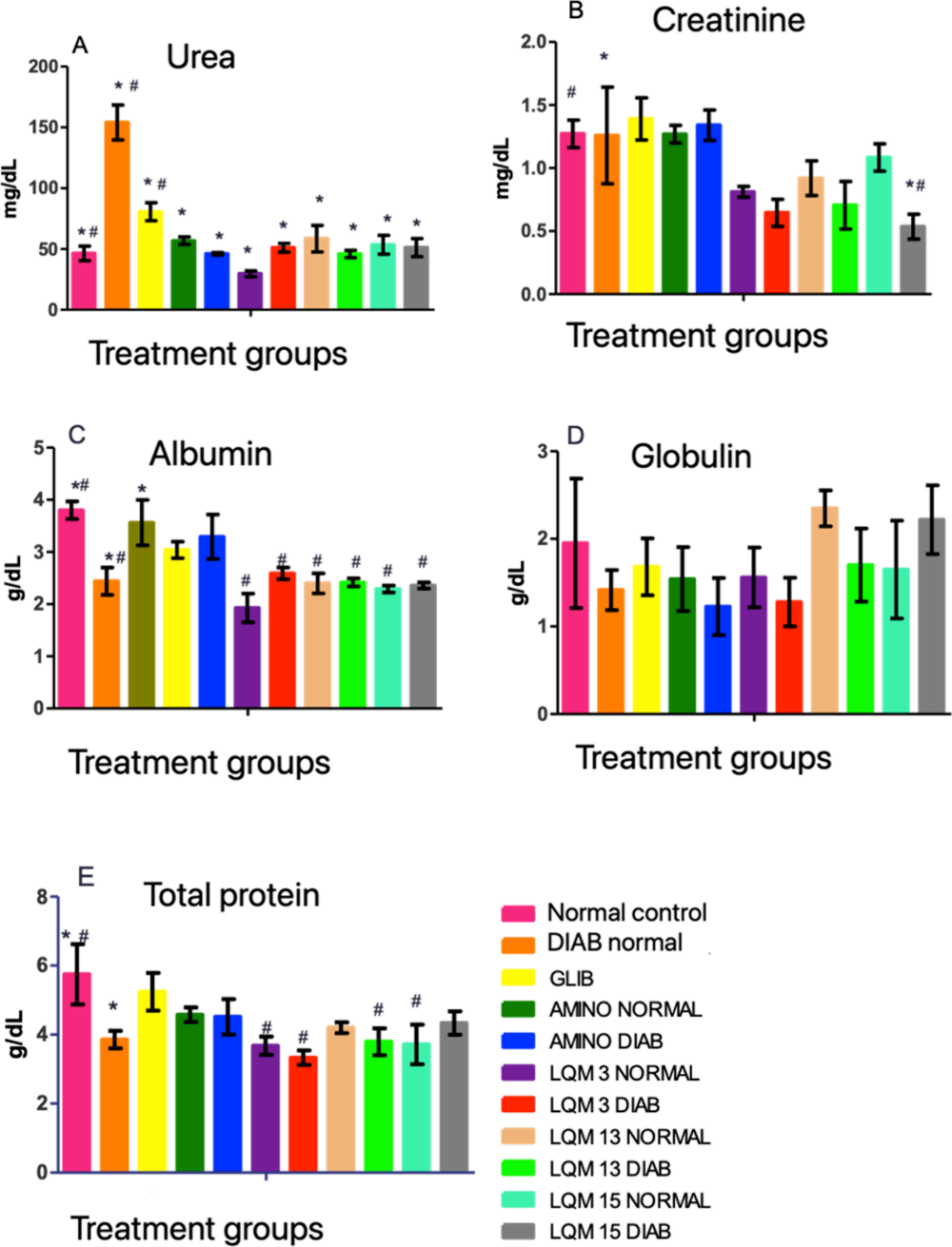
Biochemical values of renal function in diabetic and nondiabetic Wistar rats treated for 14 days with aminoguanidine derivatives and their respective controls. Notes: Means ± standard error. Means followed by *differ from diabetic negative control and followed by # differ from normal negative control using ANOVA with Dunnett’s post test considering *p* < 0.05.

Despite being used as a renal biomarker, urea does not have the same accuracy as creatinine, as the renal tubules reabsorb 40% of this product, in addition to being able to vary with protein intake, gastrointestinal bleeding and the use of some medications. Creatinine excretion is more reliable, as it only happens through the kidneys, since it cannot be reabsorbed and is not reused by the body.^39^

Therefore, when plasma creatinine levels are measured, they reflect the renal filtration rate, so that when levels are high they indicate a deficiency in renal functionality, which was not observed with the use of aminoguanidine derivatives after 14 days of treatment.

The findings of this study are in agreement with previous results described in the literature. Nayak^40^ state that the increase in AGEs in the plasma is associated with the increase in serum creatinine. Animals treated with derivatives had lower creatinine values than controls.

Liver enzymes under normal circumstances reside within hepatocytes. In the present study, the TGO and TGP values were very close to the nondiabetic negative control animals, suggesting that the acute use of derivatives may not be hepatotoxic.

Just like Nico^28^ who studied the action of aminoguanidine on liver and kidney function in rats induced chronic liver cirrhosis and even in this context, aminoguanidine was able to improve liver and kidney function.

Although alkaline phosphatase varied in all groups, the values presented are within the reference range of 56–153 U/L. The literature states that this variation can occur during pharmacological treatments.^41^

Triglyceride production is increased in insulin resistant states and diabetes, leading individuals to a higher risk of cardiovascular disease.^42^ With regard to serum levels of triglycerides, a significant decrease was detected in rats treated with the three aminoguanidine derivatives. All animals had significantly lower values than the animals in the diabetic and nondiabetic controls (Table 8).

**Table 8:** Lipid Profile Diabetic and Nondiabetic Wistar Rats after Treatment with Aminoguanidine Derivatives and Their Controls^a^

		parameters			
groups	total cholesterol	triglycerides	HDL	LDL	VLDL
CND	143.0 ± 16.5^*a^	78.3 ± 11.1*	50.7 ± 8.8*	75.3 ± 8.3*	15.7 ± 2.2*
GLI	168.0 ± 9.1^a^	112.1 ± 12.4*	19.2 ± 2.1*^b^	126.4 ± 9.8*^c^	22.4 ± 2.5*
amino D	118.7 ± 11.3	91.4 ± 14.4	31.5 ± 5.9*	68.9 ± 10.4	18.3 ± 2.9
LQM 03D	40.8 ± 6.1*^a^	62.9 ± 0.27	14.4 ± 0.9*^b^	23.5 ± 6.2*^c^	12.6 ± 0.1
LQM 13D	48.3 ± 3.2*^a^	63.4 ± 0.36	16.3 ± 1.5*^b^	28.7 ± 3.2*^c^	12.7 ± 0.1
LQM 15D	45.5 ± 3.6*^a^	62.2 ± 0.07	16.8 ± 0.8*^b^	25.3 ± 4.2*^c^	12.4 ± 0.0
CNN	100.5 ± 13.6^a^	81.3 ± 13.3	38.1 ± 7.0^b^	46.1 ± 13.4*^c^	16.2 ± 2.6
amino N	122.1 ± 8.3	75.0 ± 9.2	41.0 ± 3.7	66.2 ± 11.4	15.0 ± 1.8
LQM 03N	53.0 ± 3.7*^a^	63.5 ± 0.55	16.7 ± 0.7*^b^	33.0 ± 4.1*^c^	12.7 ± 0.1
LQM 13N	52.8 ± 3.2*^a^	62.6 ± 0.29	14.6 ± 0.4*^b^	35.2 ± 3.0*^c^	12.5 ± 0.1
LQM 15N	37.8 ± 2.4*^a^	62.1 ± 0.16	15.3 ± 0.6*^b^	19.5 ± 2.5*^c^	12.4 ± 0.0

a Mean + standard error. Means followed by * in the same column differ from the diabetic negative control (CND) and means followed by the same letter in the column differ from the normal negative control (CNN) and by ANOVA with Dunnett’s post-test considering *p* < 0.05.

In view of the effects on the reduction of serum triglycerides and other lipid parameters, studies can be suggested for the use of these derivatives in the treatment of dyslipidemia. However, this consideration needs to be investigated in more detail, including in animal models of dyslipidemia.

Thus, the evaluation of the antidiabetic and antiglycant activity of the aminoguanidine derivatives presented promising and interrelated data, as they present low toxicity, act significantly in the AGES in vitro and in vivo, with a reduction in the values of glycated hemoglobin and fructosamine, in addition to having an action antidiabetic activity in Wistar rats, with streptozotocin induction, maintaining biochemical standards for renal and hepatic function within normal limits.

## Conclusions

Based on the results obtained under the experimental conditions of this study, it can be concluded that the aminoguanidine derivatives were safe in the in vitro cytotoxicity assay and showed no toxic effects. Seventeen out of the 19 derivatives remained nontoxic when cells were treated with a concentration of 10 μM At a concentration of 100 μM, 10 of these derivatives maintained cell viability above the standard threshold of 80%.

Derivatives LQM 03, 05, 06, 07, 13, 16, and 17 had excellent action in inhibiting the formation of glycation end products in vitro. LQM 13 reached up to 94.3% AGEs inhibition, which result was superior to aminiguanidine.

In in vivo studies, derivatives LQM 03, 13, and 15 were not toxic, that is, the animals did not lose weight, nor did they show behavioral, biochemical or histological alterations that could be attributed to the use of derivatives.

In tests for antidiabetic and antiglycant activity, these three derivatives were able to reduce blood glucose levels by up to 65%, which is significant when compared with glimepiride, the standard drug used. Furthermore, the levels of fructosamine and glycated hemoglobin remained low in the groups treated with the derivatives, with biochemical parameters that did not indicate nephrotoxicity or hepatotoxicity.

Therefore, it is possible to conclude that the tested derivatives have potential for further studies, these represent prototypes of promising drugs, so that in the future it will be possible to have a product capable of reducing glycemic indices and at the same time having an antiglicant action, protecting individuals from macro and diabetes microvascular.

## Supplementary Material



## Notes

^1^H NMR and 13C NMR spectra for all compounds (PDF)
