# Supplementary material for: Antidiabetic Activity and Inhibitory Effects of Derivatives of Advanced Aminoguanidine Glycation
Source: ACS Omega. 2025 Oct 7;10(41):47914–27. doi: 10.1021/acsomega.5c01940 (PMC12547601; doi:10.1021/acsomega.5c01940)
Supplement: Supplementary file 1 [file ao5c01940_si_001.pdf]

Antidiabetic Activity and Inhibitory Effects of Derivatives of Advanced Aminoguanidine Glycation

Patrícia de Albuquerque Sarmento,[1] Andressa Letícia Lopes da Silva,[2] Edeildo Ferreira da Silva-Júnior, [3] Elita Scio,[4] Danielle Maria de Oliveira Aragão, [4] Êurica Adélia Nogueira Ribeiro,[5] Érica Erlanny da Silva Rodrigues, [6] Pedro Gregório Vieira Aquino,[7] Bárbara Viviana de Oliveira Santos, [8] Antônio Euzébio Goulart Santana, [9] Aline Cavalcanti de Queiroz, [2] Magna Suzana Alexandre-Moreira, [1] Henrique Douglas Melo Coutinho, [10]* João Xavier de Araújo Júnior [3]*

[1]Wound Treatment Research Laboratory – LPTF/School of Nursing. Federal University of Alagoas

[2] Pharmacology and Immunity Laboratory-LAFI/Institute of Biological and Health Sciences. Federal University of Alagoas

[3] Biological and Molecular Chemistry Research Group (GPQBioMol), Institute of Chemistry and Biotechnology. Federal University of Alagoas

[4] Laboratory of Bioactive Natural Products - LPNB Institute of Biological Sciences. Federal University of Juiz de Fora

[5] Cardiovascular Pharmacology Laboratory (LFC)/ Institute of Pharmaceutical Sciences (ICF). Federal University of Alagoas

[6] Medicinal Chemistry Laboratory (LQM)/Institute of Pharmaceutical Sciences (ICF). Federal University of Alagoas

[7] Research Group on Bioactive Substances of Natural and Synthetic Origin. Federal University of Agreste de Pernambuco

[8] Teacher Training Center, UACEN. Federal University of Campina Grande

[9] Natural Products Research Laboratory. Federal University of Alagoas

[10] Laboratory of Microbiology and Molecular Biology (LMBM). Regional University of Cariri

* Corresponding authors: HDM Coutinho (hdmcoutinho@gmail.com); JX de A Junior (jotaaraujo2004@gmail.com)

Supporting Information

All reagents and starting solvents were purchased from Merck/Sigma-Aldrich® (St. Louis, MO, USA) and were commercially available with high purity (>98%).

The purity of the respective compounds was determined using high-pressure liquid chromatography coupled to an ultraviolet detector at a wavelength (λ) of 254 nm (HPLC/DAD), using a Shimadzu® SIL-20AHT equipment and a C-18 Supelco Discovery® column, 25 cm x 4.6 mm, 5 µM. 100% methanol was used as the mobile phase. The sample injection volume was 10 µL and the injection flow rate was 1 mL/min. The samples were analyzed in 10-minute runs. The ^{1}H and ^{13}C NMR spectra were obtained using a Brüker® Avance DRX 600 MHz – UltraShield® equipment from the Center for Product Analysis by Nuclear Magnetic Resonance – IQB/UFAL, led by Prof. Thiago Mendonça de Aquino. DMSO-d6 was used as the analytical solvent for solubilization of the peptidomimetics. Regarding the spectra, chemical shifts (δ) were computed in parts per million (ppm) and coupling constants were reported in Hertz (Hz). Tetramethylsilane (TMS) or DMSO-d6 was used as the internal reference. The signal multiplicities were indicated as follows: simplet (s), broad simplet (sl), doublet (d), triplet (t), quartet (q), quintet (qi), sextet (sex), septet (sep), and double triplet (dt). All NMR spectra were processed and analyzed using Bruker TopSpin academic license software.

LQM 01:

0.2311 g of LQM01 (0.9723 mmol) was obtained with a yield of 70.57%. **^{1}H NMR** (400 MHz, DMSO-d6): 7.12(t, 1H, J=7.2Hz); 7.19 (t, 1H, J=7.2Hz); 7.45 (d, 1H, J=7.2Hz); 7.8 (s, 1H); 8.28(d, 1H, J=7.2); 8.36 (s, 1H). **^{13}C NMR** (100 MHz, DMSO-d6):121.237; 122,757; 123,277; 124,317; 132,247; 137,515; 145,351; 155,208.

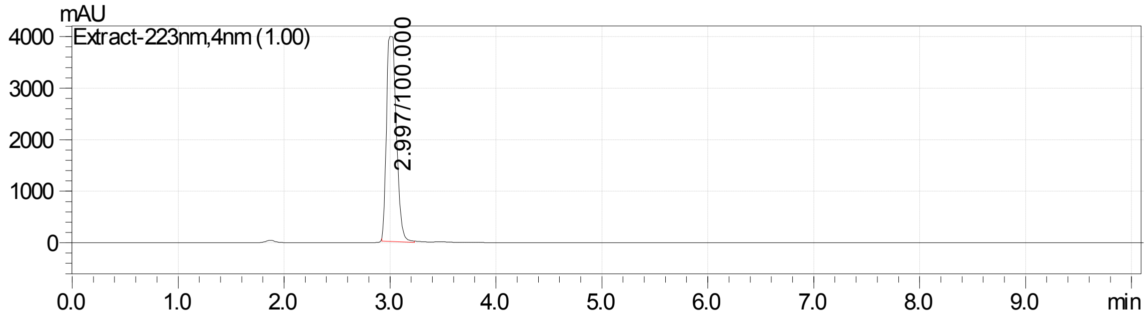

Figure S1. Chromatogram of substance LQM01

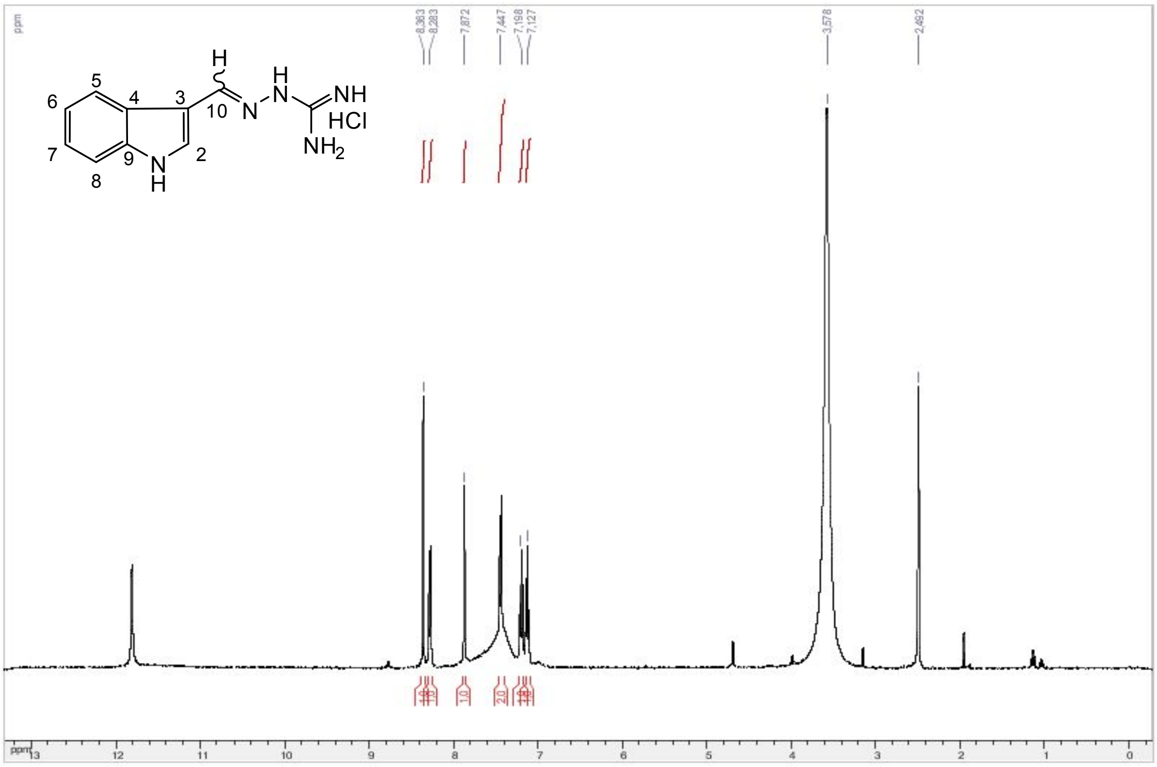

Figure S2. ^{1}H NMR (400Hz) in DMSO-d6 of LQM01

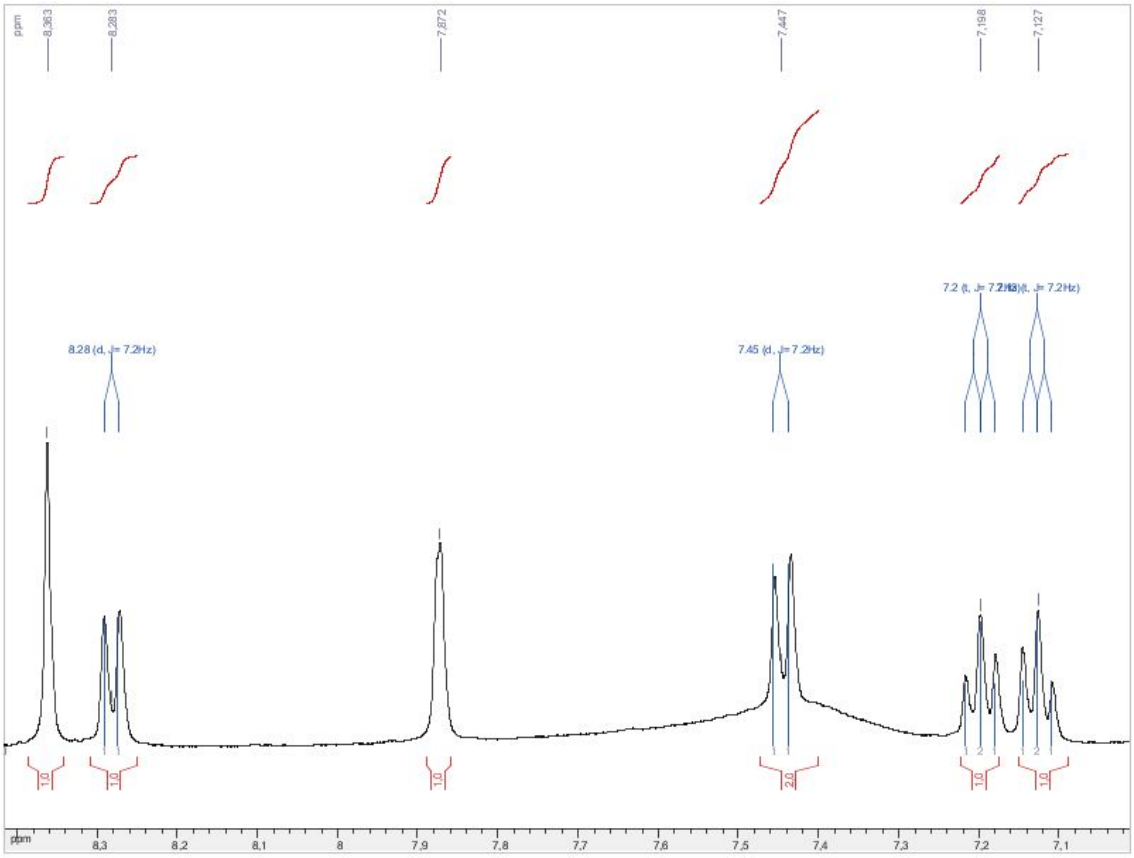

Figure S3. Enlargement of the spectrum from δ7.1 to δ8.3.

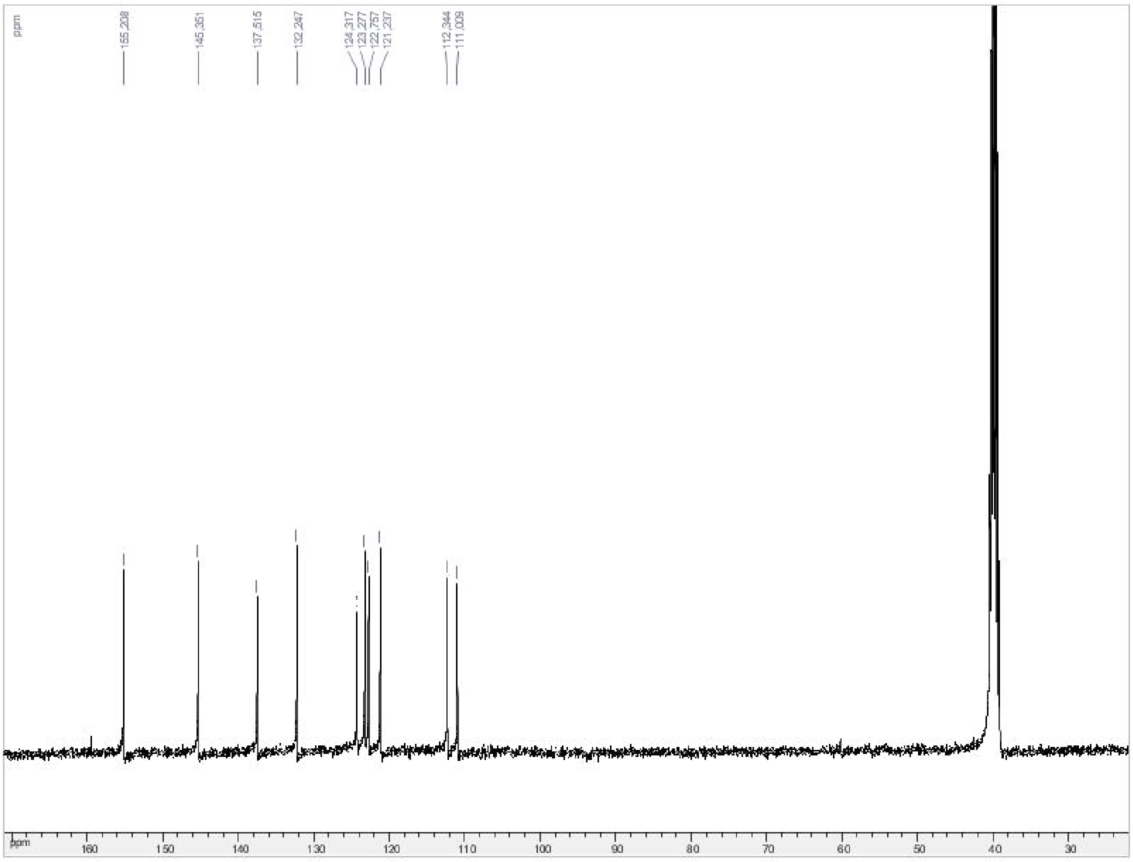

Figure S4. ^{13}C NMR (100Hz) in DMSO-d6 of LQM01.

LQM02:

0.2369g of LQM02 (1.0163 mmol) was obtained with a yield of 71.32%.**^{1}H NMR** (400 MHz, DMSO-d6): 7.45(d, 2H, J=8.5Hz); 7.85 (d, 2H, J=8.5Hz); 8.15 (s, 1H). **^{13}C NMR** (100 MHz, DMSO-d6): 129.236; 129,709; 132,818; 135,439; 146,013; 155,830.

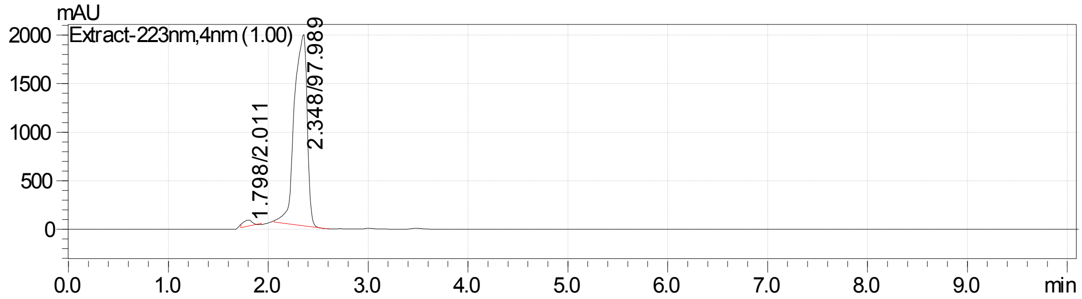

Figure S5. Chromatogram of substance LQM02

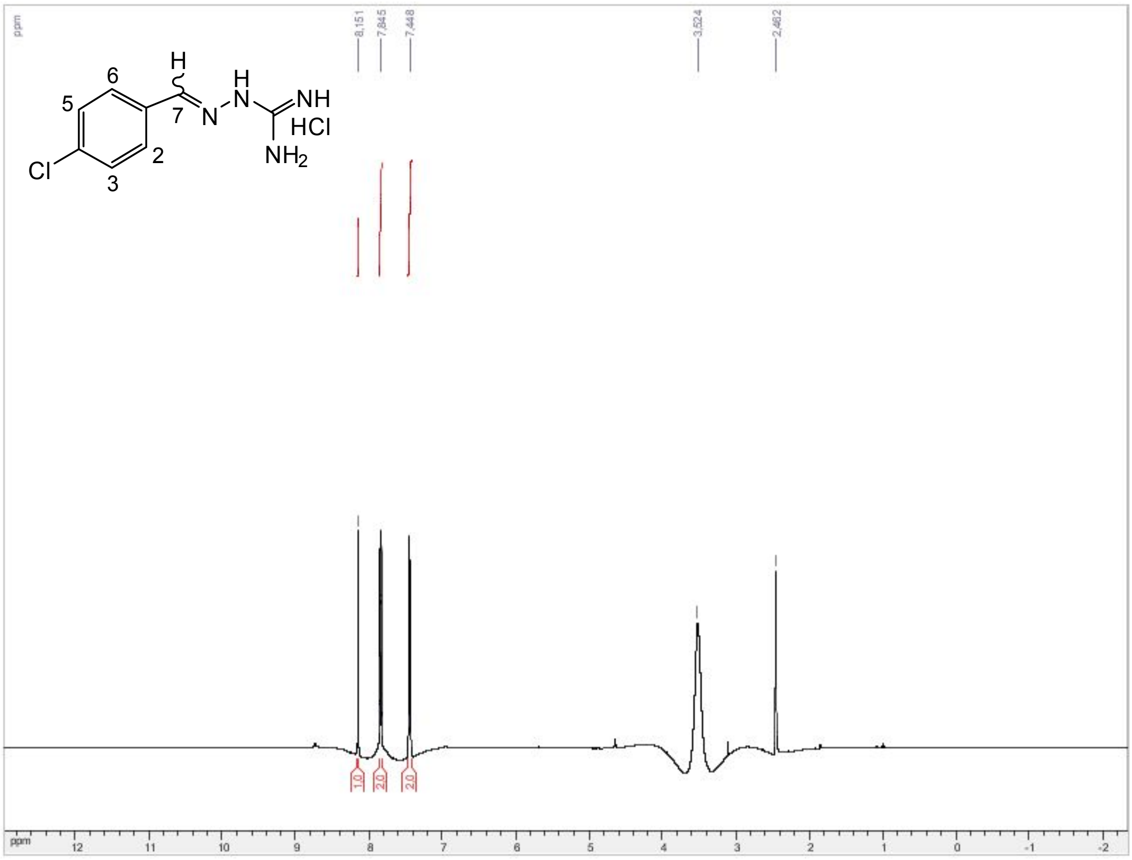

Figure S6. 1H NMR (400Hz) spectrum in DMSO-d6 of LQM02.

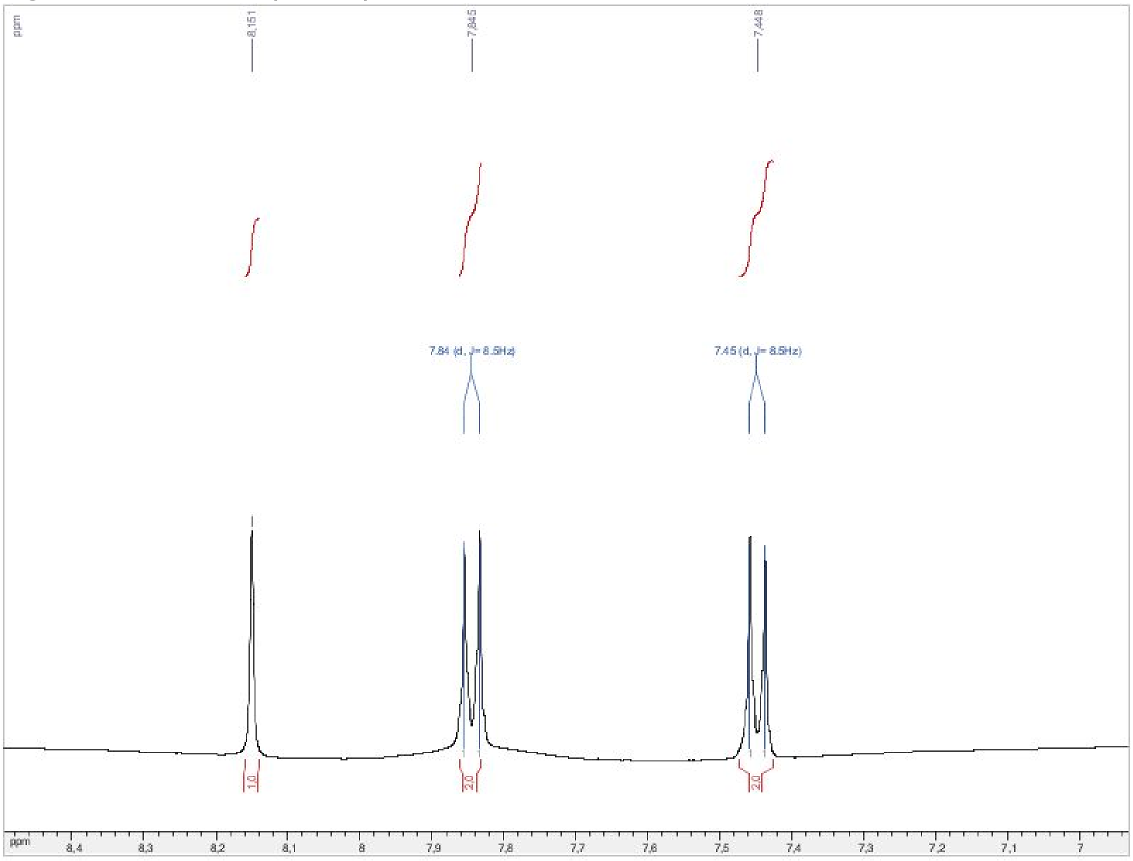

Figure S7. Enlargement of the spectrum from δ7.0 to δ8.4.

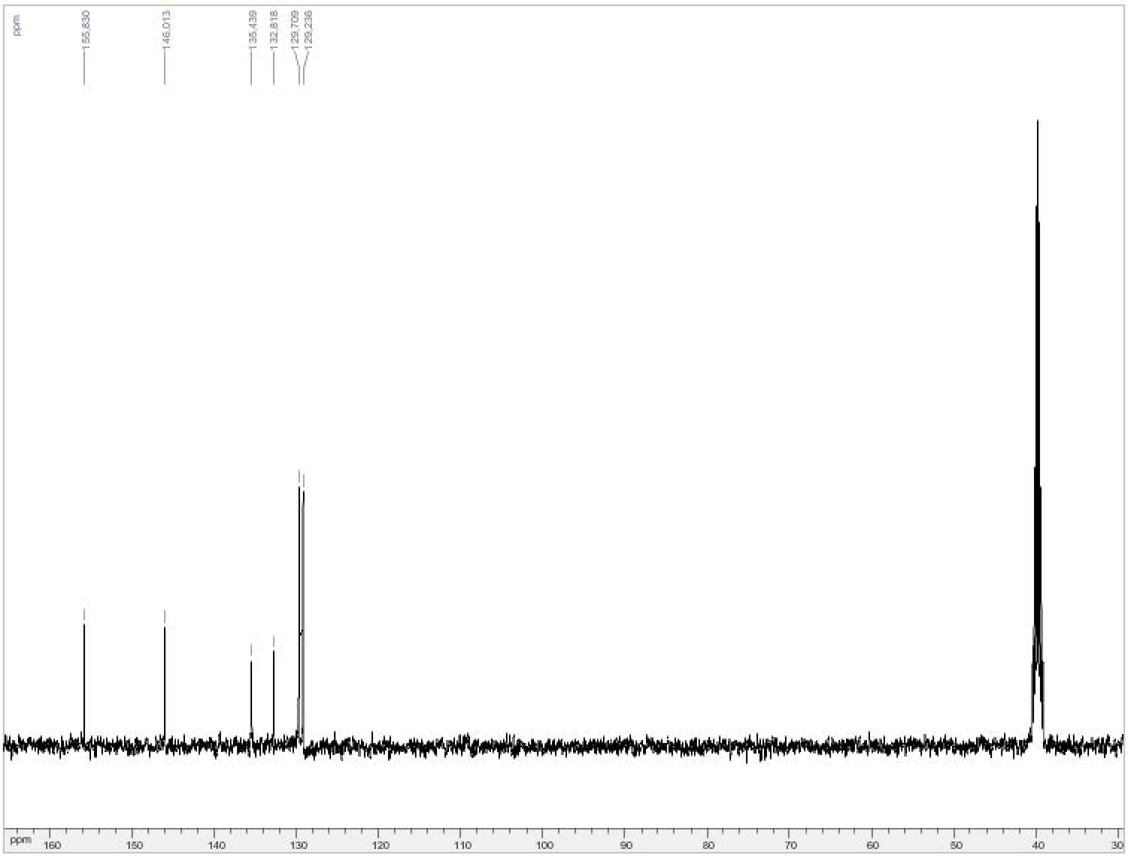

Figure S8. ^{13}C NMR (100Hz) in DMSO-d6 of LQM02.

LQM03:

0.3023 g of substance 3 (1.3515 mmol) was obtained with a yield of 88.53%. **^{1}H NMR** (400 MHz, DMSO-d6): 7.87 (d, 2H, J=8.5); 8.04 (d, 2H, J=8.5Hz); 8.24 (s, 1H,). **^{13}C NMR** (100 MHz, DMSO-d6): 112.671; 119.117; 128.607; 133.029; 138.301; 145.362; 155.895.

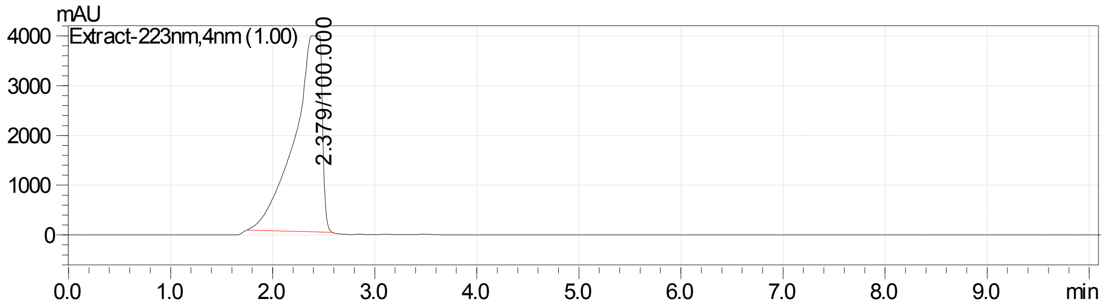

Figure S9. Chromatogram of substance LQM03

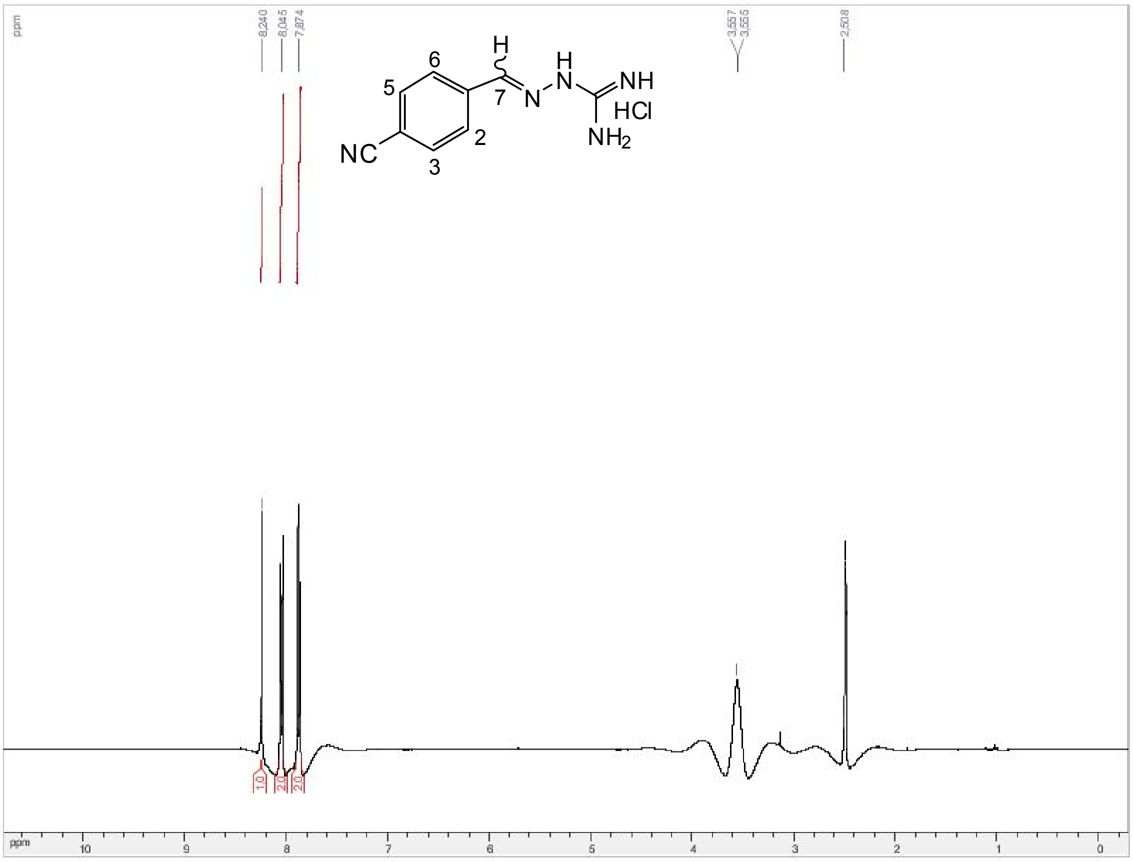

Figure S10. 1H NMR (400Hz) in DMSO-$_{d6}$ of LQM03.

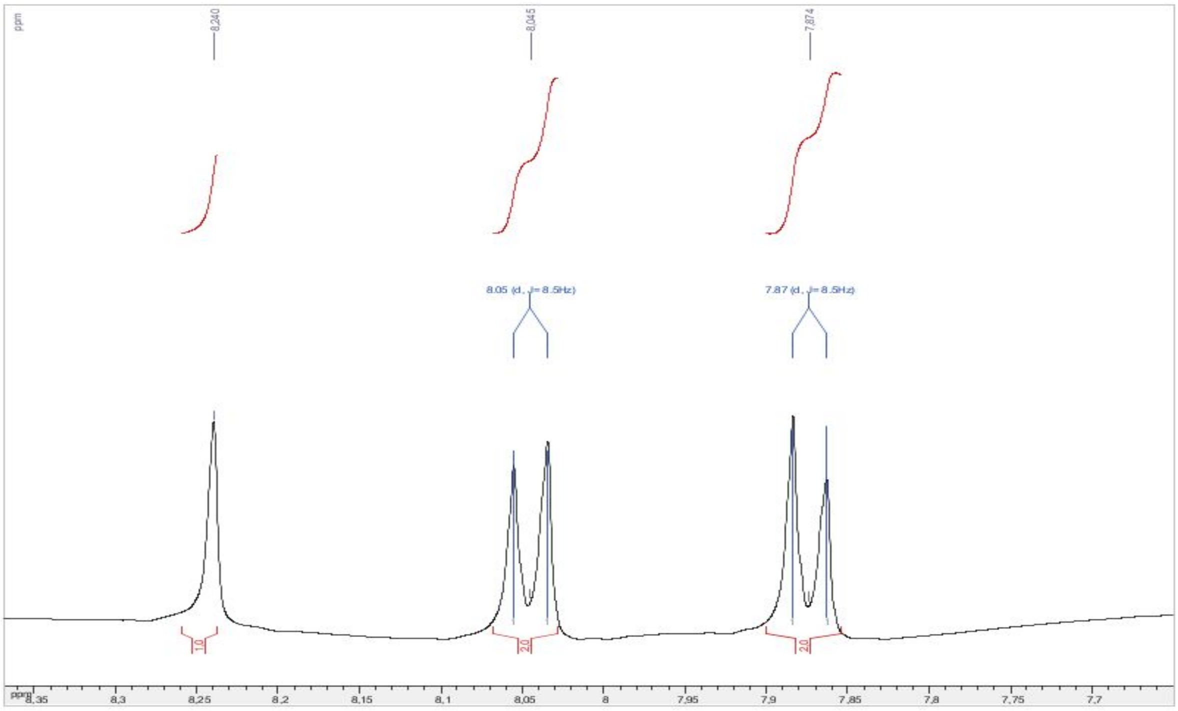

Figure S11. Enlargement of the spectrum from δ7.6 to δ8.4.

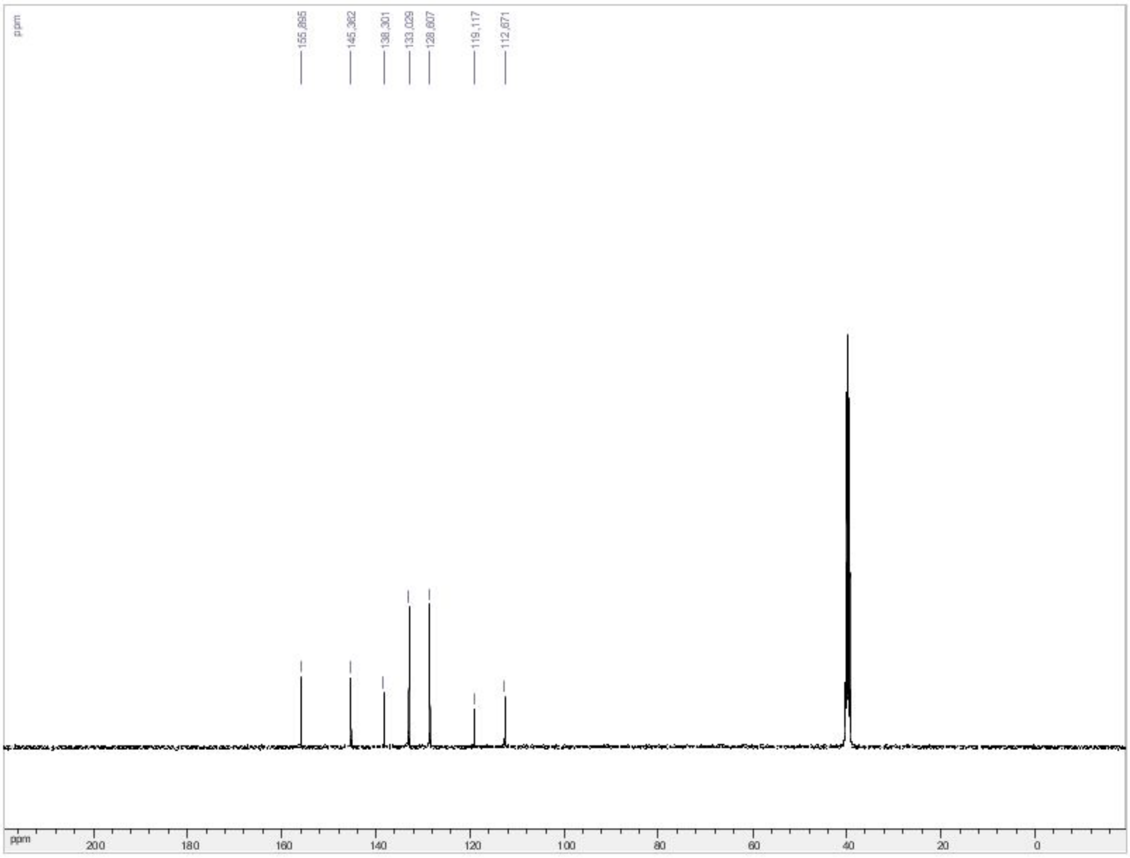

Figure S12. 13C NMR (100Hz) in DMSO-d6 of LQM03.

LQM04:

0.2857 g of substance 4 (1.1676 mmol) was obtained with a yield of 92.27%. **^{1}H NMR** (400 MHz, DMSO-d6): 3.79 (s, 3H); 6.95 (d, 1H, J=8.3); 7.18 (dd, 1H, J=8.3Hz and 2.0Hz); 7.33 (d, 1H, J=2.0Hz) 8.04 (s, 1H,). **^{13}C NMR** (100 MHz, DMSO-d6): 56.11; 112.053; 113.813; 121.095; 126.637; 147.063; 147.627; 150.499; 155.615.

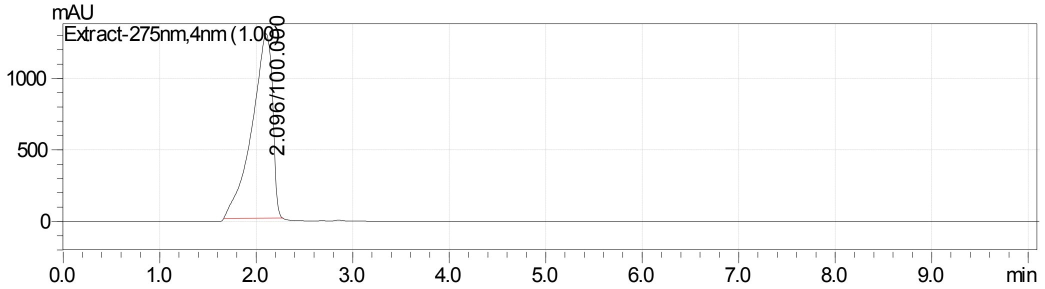

Figure S13. Chromatogram of substance LQM04

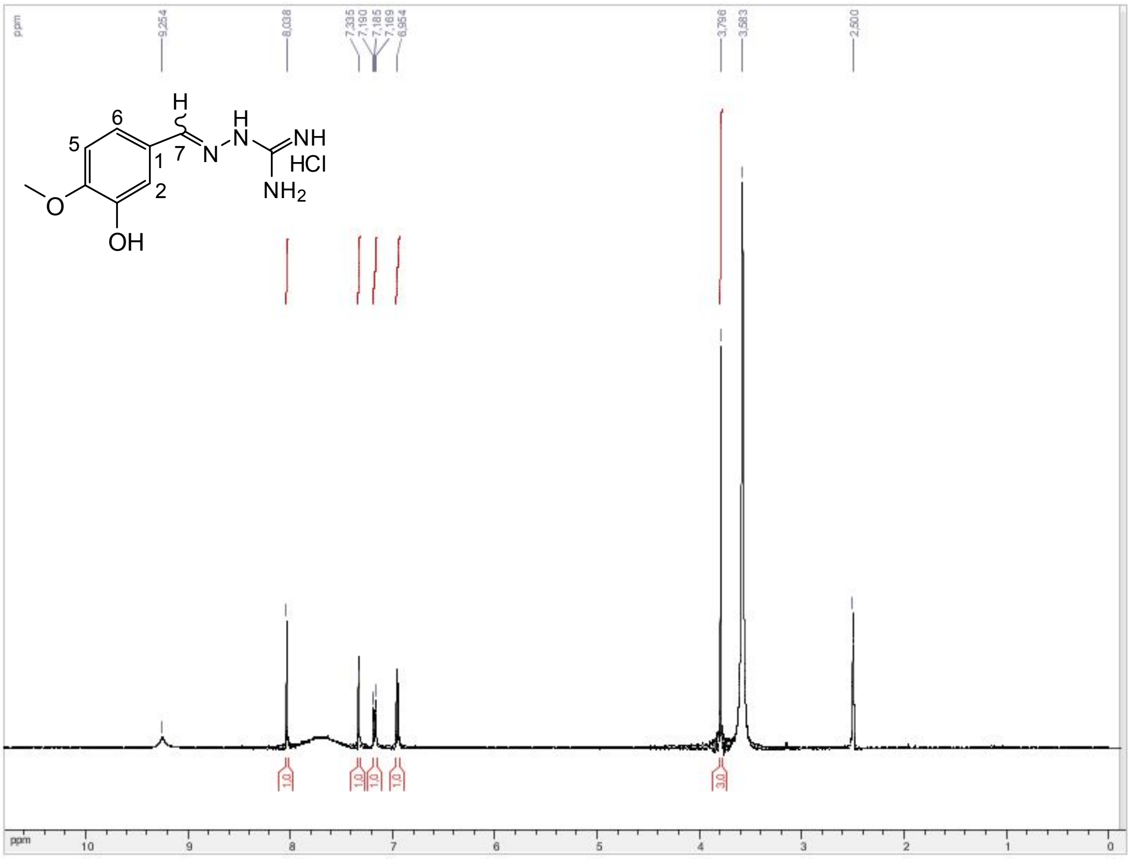

Figure S14. ^{1}H NMR (400Hz) in DMSO-*d6* of LQM04.

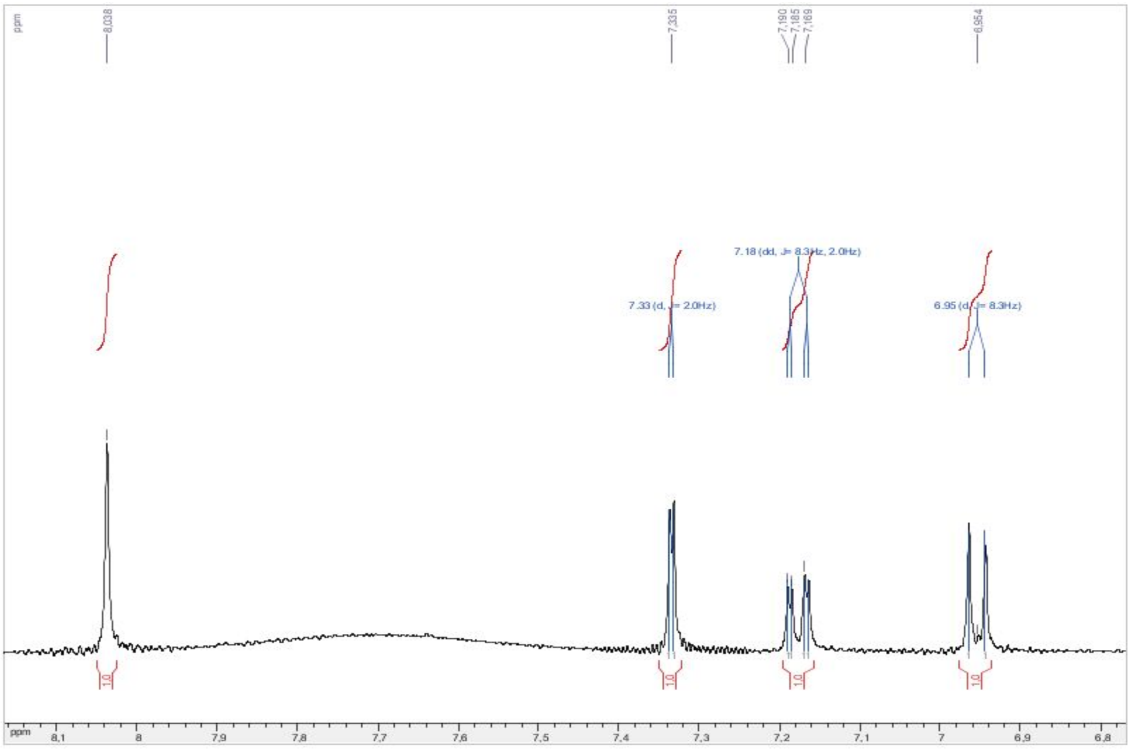

Figure S15. Enlargement of the spectrum from δ7.6 to δ8.4.

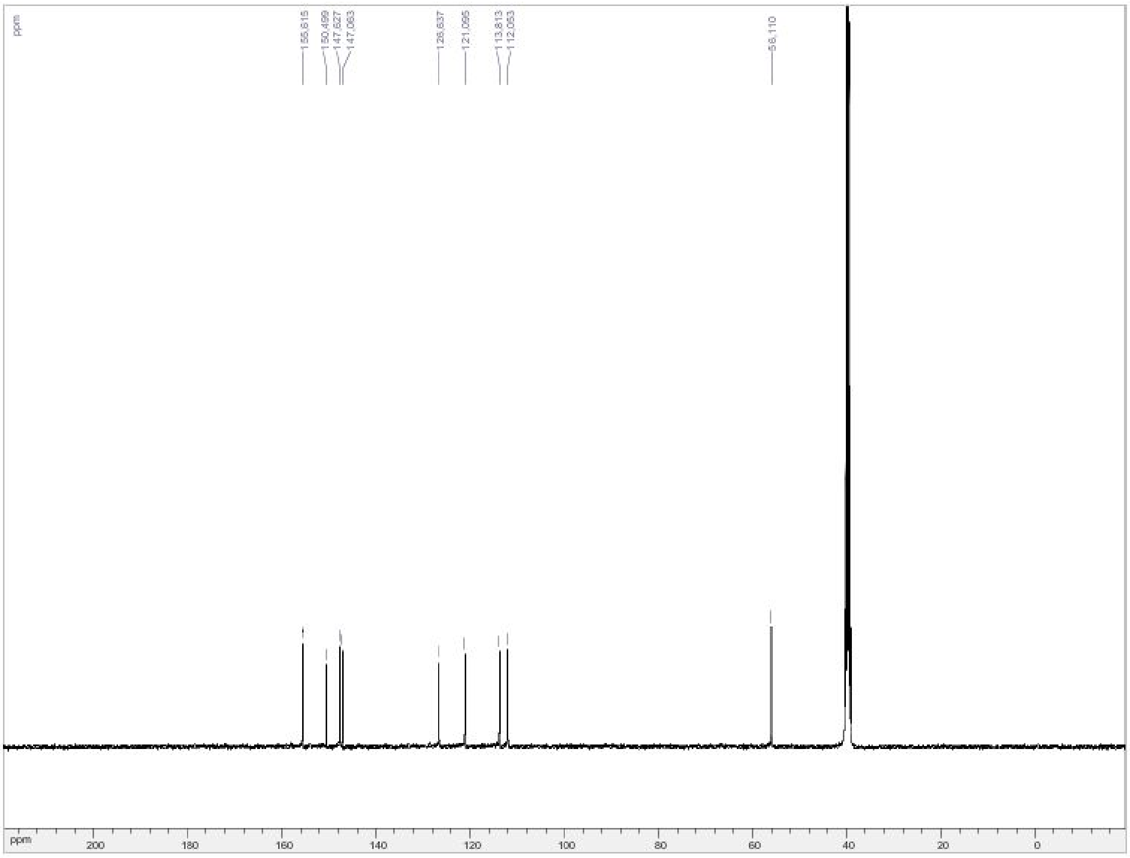

Figure S16. ^{13}C NMR (100Hz) in DMSO-*d6* of LQM04.

LQM05:

0.2889 g of substance 5 (1.1167 mmol) was obtained with a yield of 92.65%.

^{1}H NMR (400 MHz, DMSO-d6): 3.80 (s, 6H); 6.97 (d, 1H, J=7.9); 7.23 (d, 1H, J=7.9 Hz); 7.54 (s, 1H); 8.09 (s, 1H). **^{13}C NMR** (100 MHz, DMSO-d6): 109.173; 111.606; 123.099; 126.535; 147.391; 149.51; 151.474; 155.633; 155.702; 56.168.

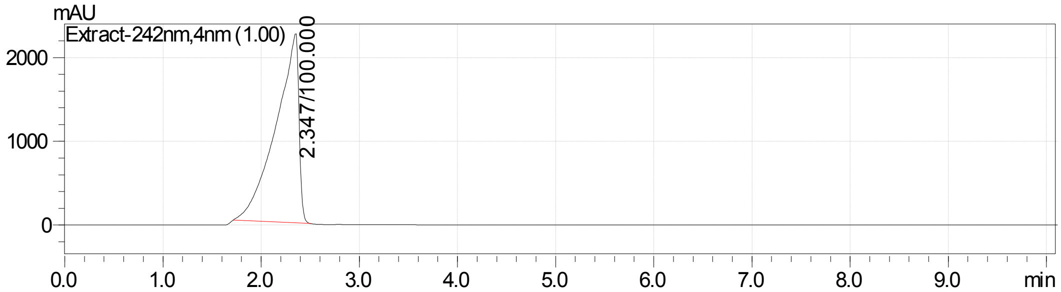

Figure S17. Chromatogram of substance LQM05

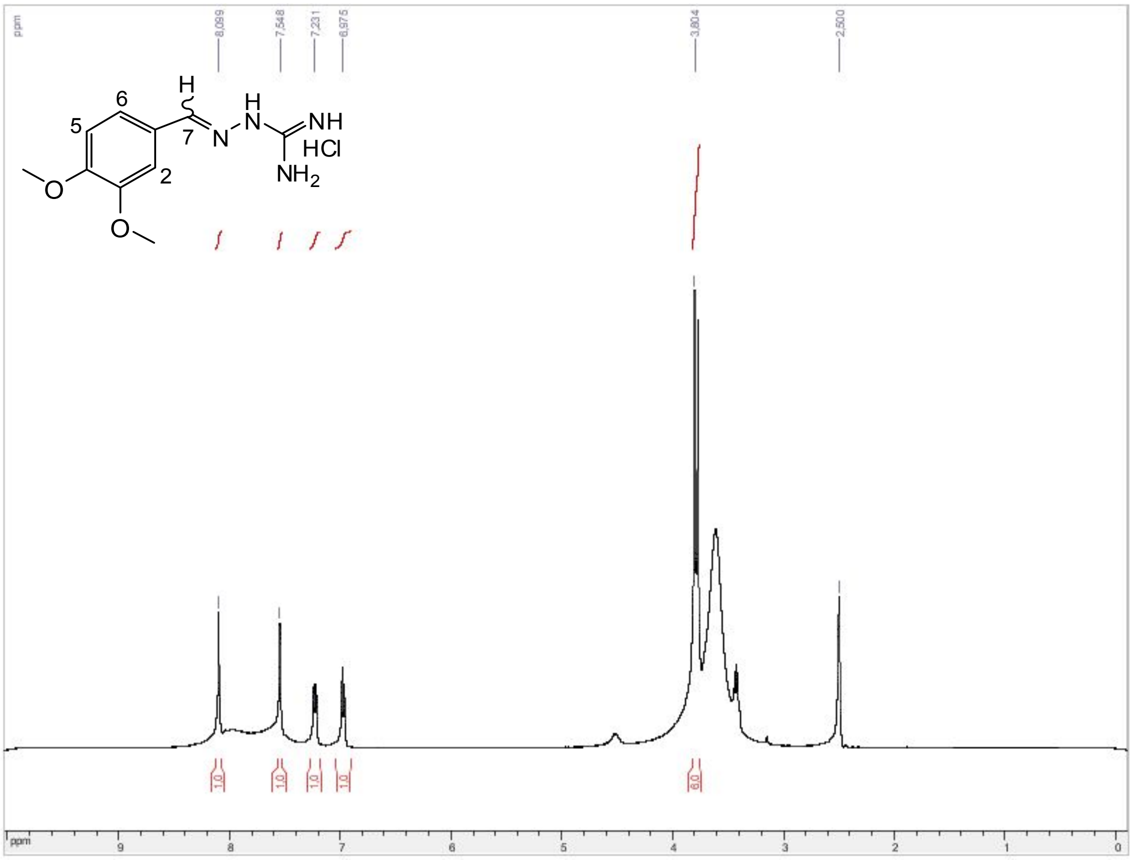

Figure S18. ^{1}H NMR (400Hz) spectrum in DMSO-*d6* of LQM05

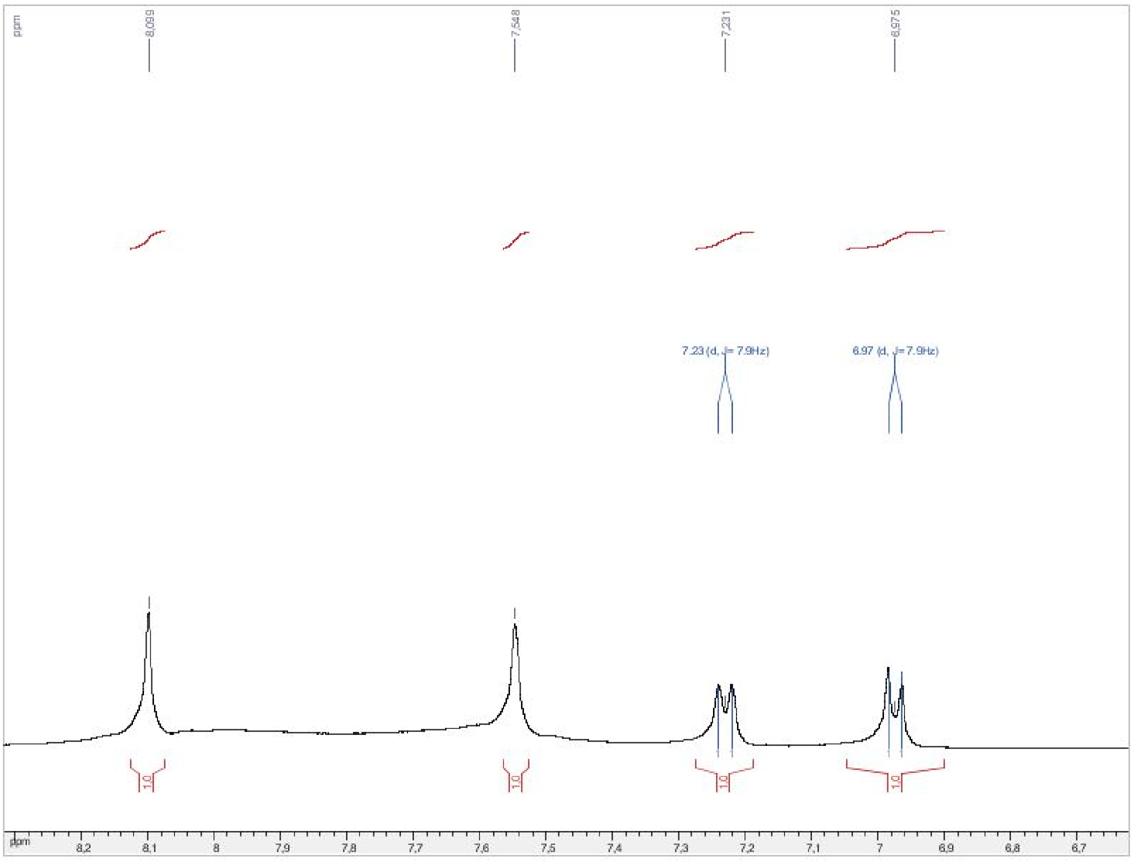

Figure S19. Enlargement of the spectrum from δ7.6 to δ8.5.

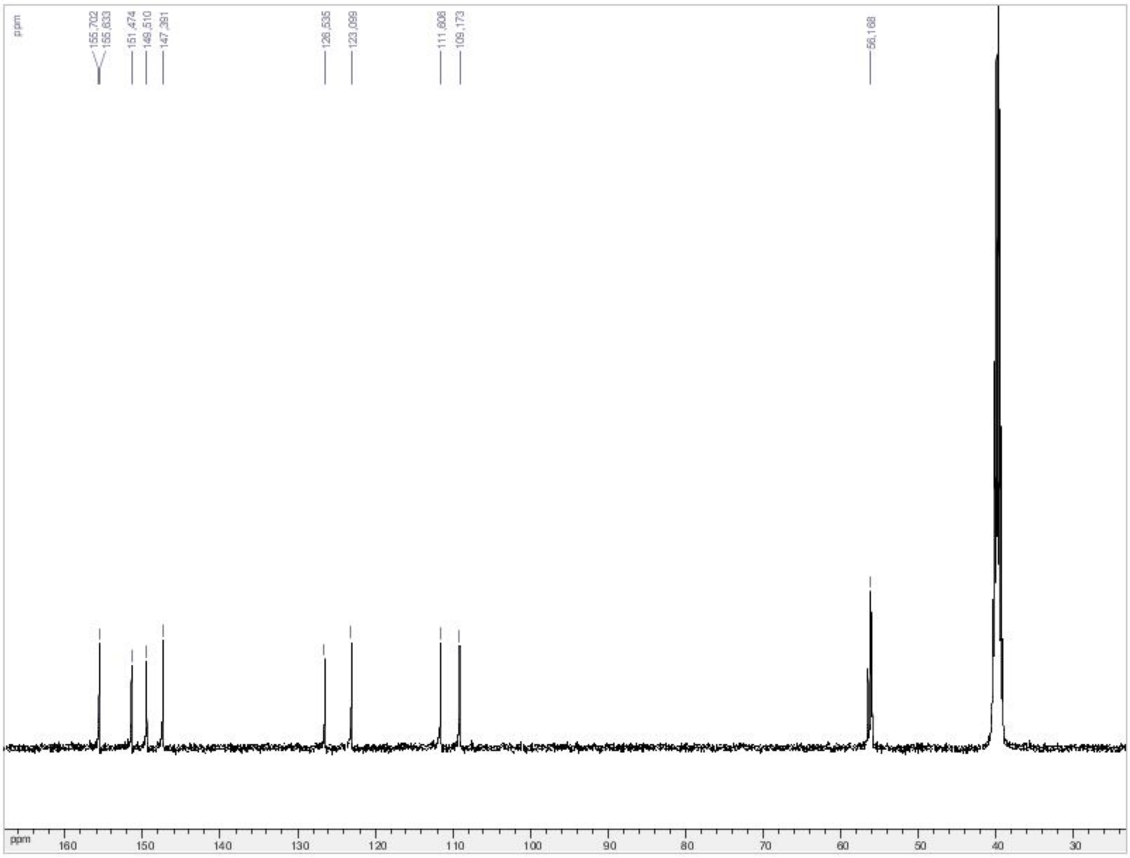

Figure S20. ^{13}C NMR (100Hz) in DMSO-d6 of LQM05.

LQM06:

0.3175 g of substance 6 (1.2976 mmol) was obtained with a yield of 98.27%. **^{1}H NMR** (100 MHz, DMSO-d6): 3.81 (s, 3H); 6.83 (d, 1H, J=8.0); 7.13 (dd, 1H, J=8.0Hz and 1.8Hz); 7.50 (1.8, 1H); 8.04 (s, 1H). **^{13}C NMR** (400 MHz, DMSO-d6): 56.241; 110.082; 115.671; 123.182; 125.222; 147.74; 148.514; 149.787; 155.524.

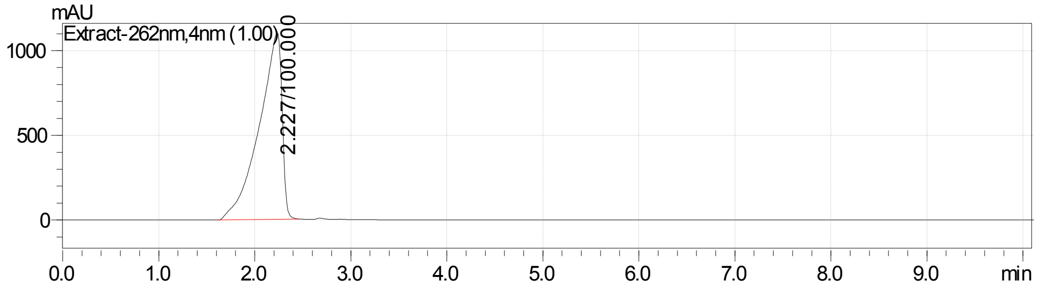

Figure S21. Chromatogram of substance LQM06

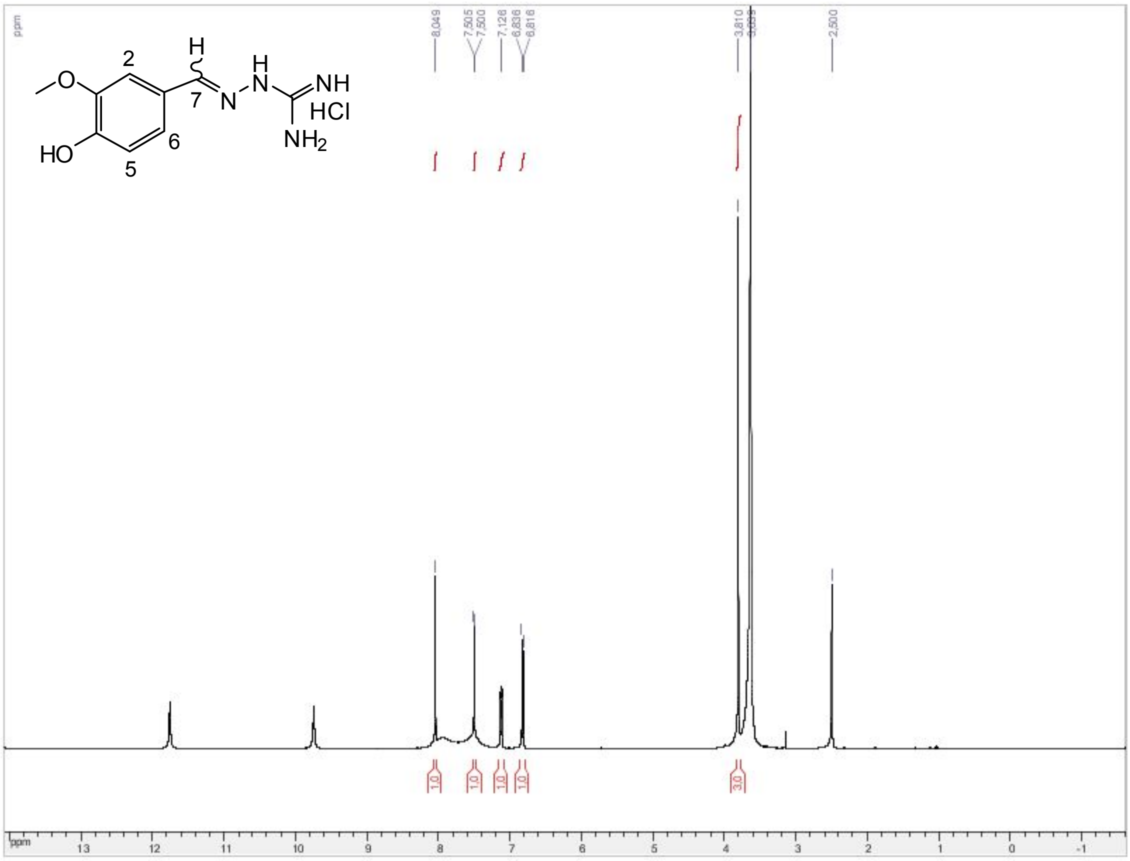

Figure S22. ^{1}H NMR (400Hz) in DMSO-d6 of LQM06.

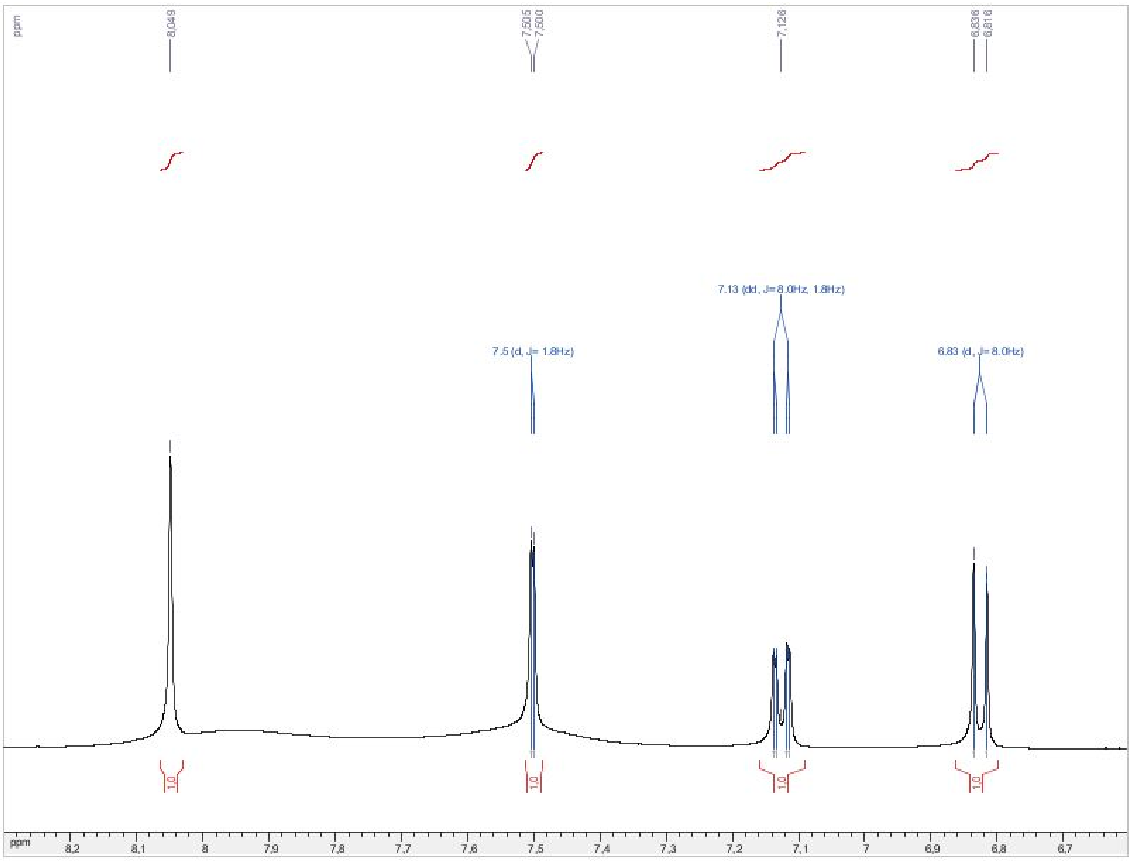

Figure S23. Enlargement of the spectrum from δ7.6 to δ8.4.

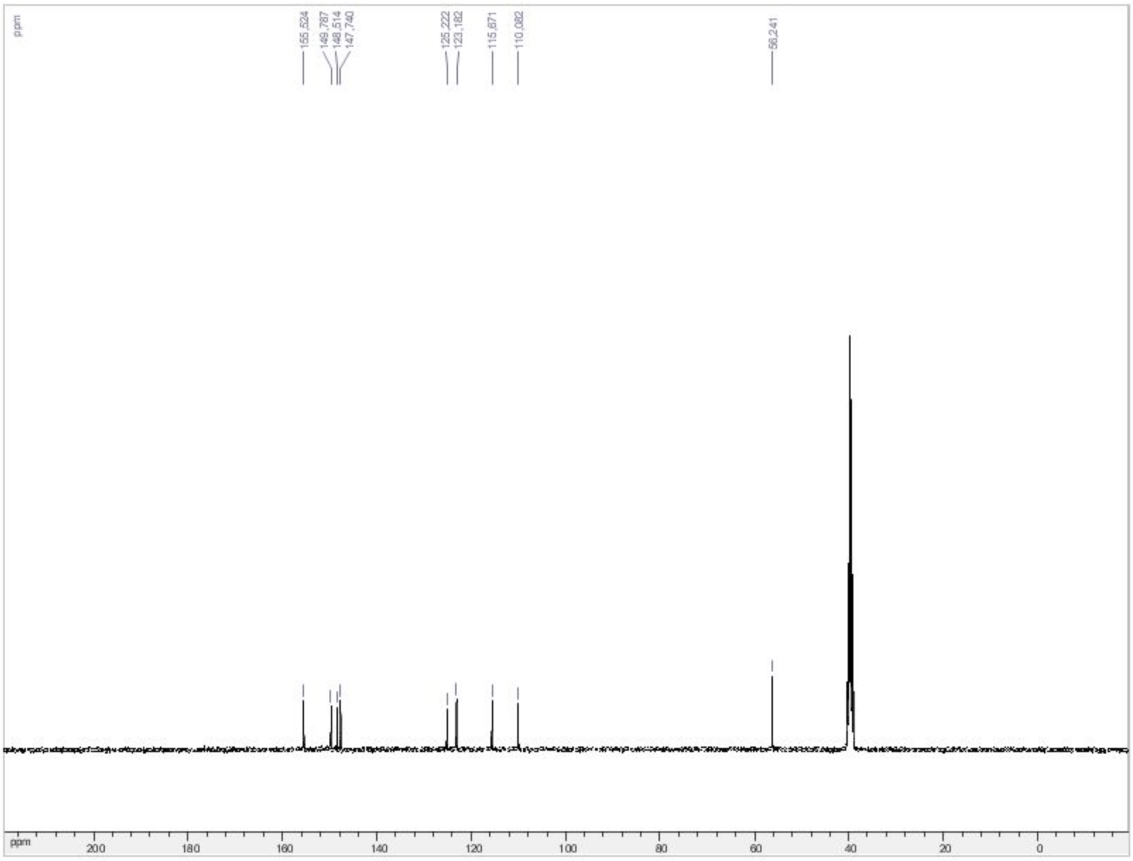

Figure S24. ^{13}C NMR (100Hz) in DMSO-*d6* of LQM06.

LQM07:

0.3357g of substance 7 (1.5639 mmol) was obtained with a yield of 95.06%.

^{1}H NMR (400 MHz, DMSO-d6): 6.83 (d, 2H, J=8.5); 7.66 (d, 2H, J=8.5); 8.06 (s, 1H). **^{13}C NMR** (100 MHz, DMSO-d6): 116.067; 124.797; 129.854; 147.522; 155.622; 160.287.

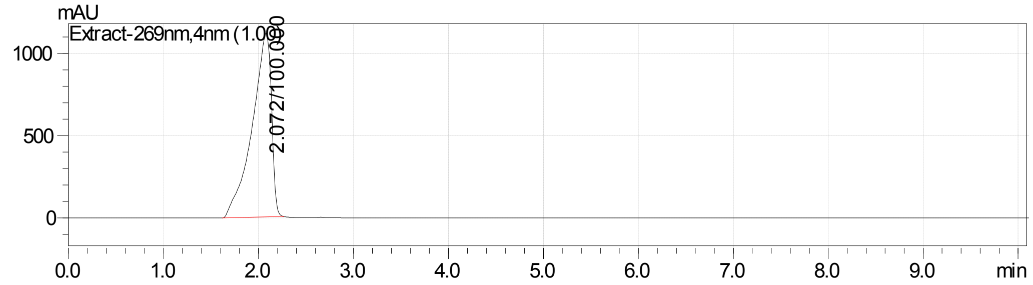

Figure S26. Chromatogram of substance LQM07

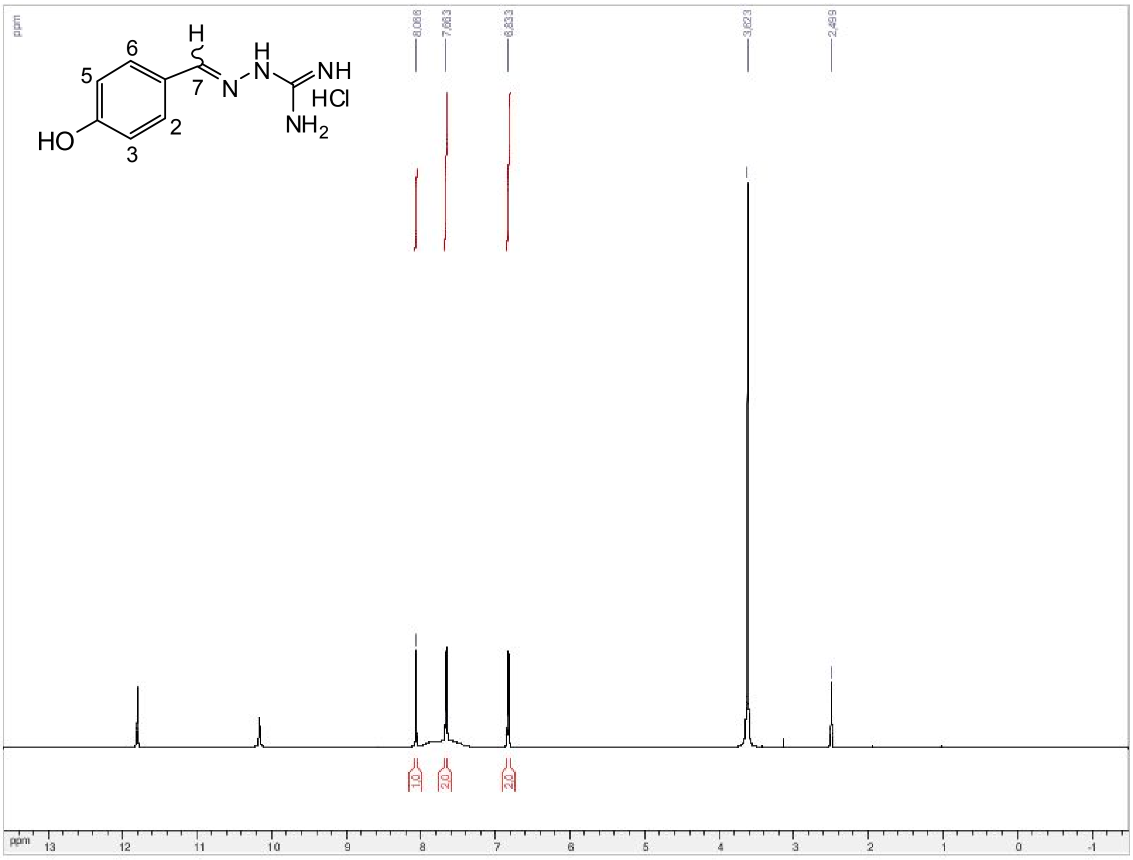

Figure S27. ^{1}H NMR (400Hz) in DMSO-d6 of LQM07.

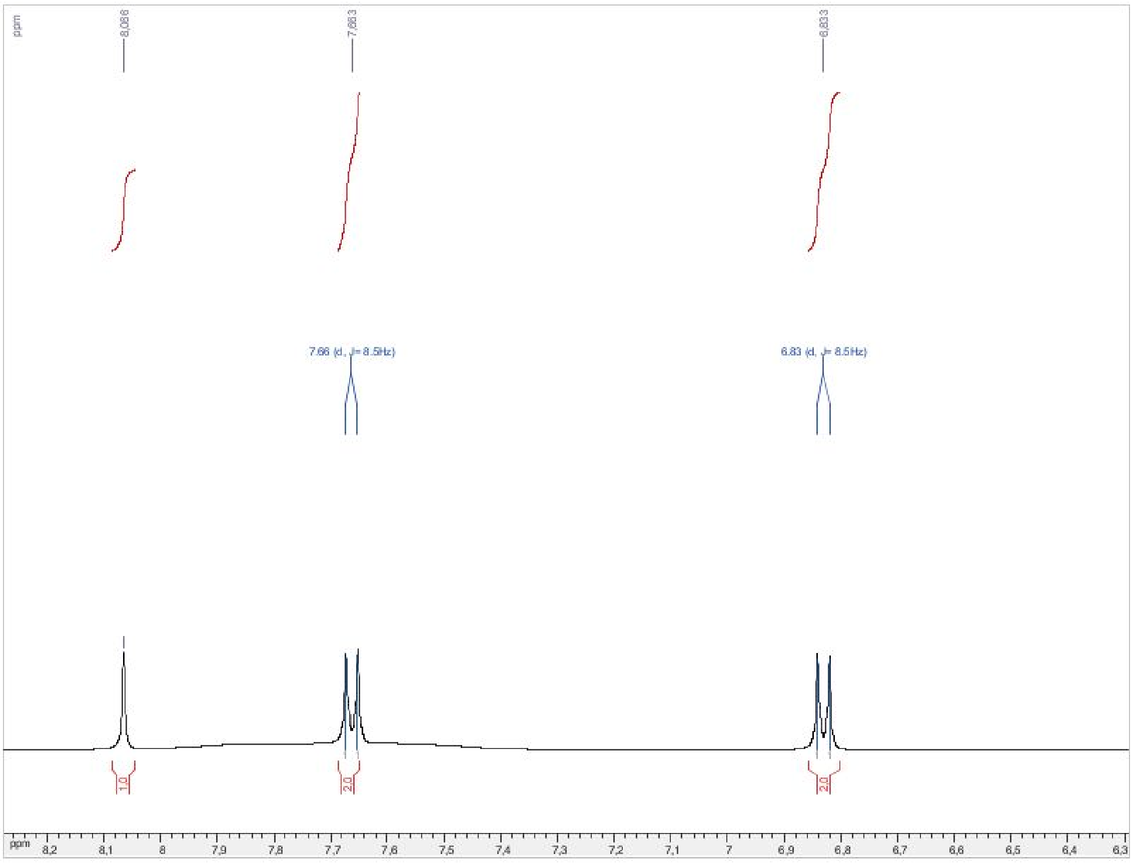

Figure S28. Enlargement of the spectrum from δ7.6 to δ8.4.

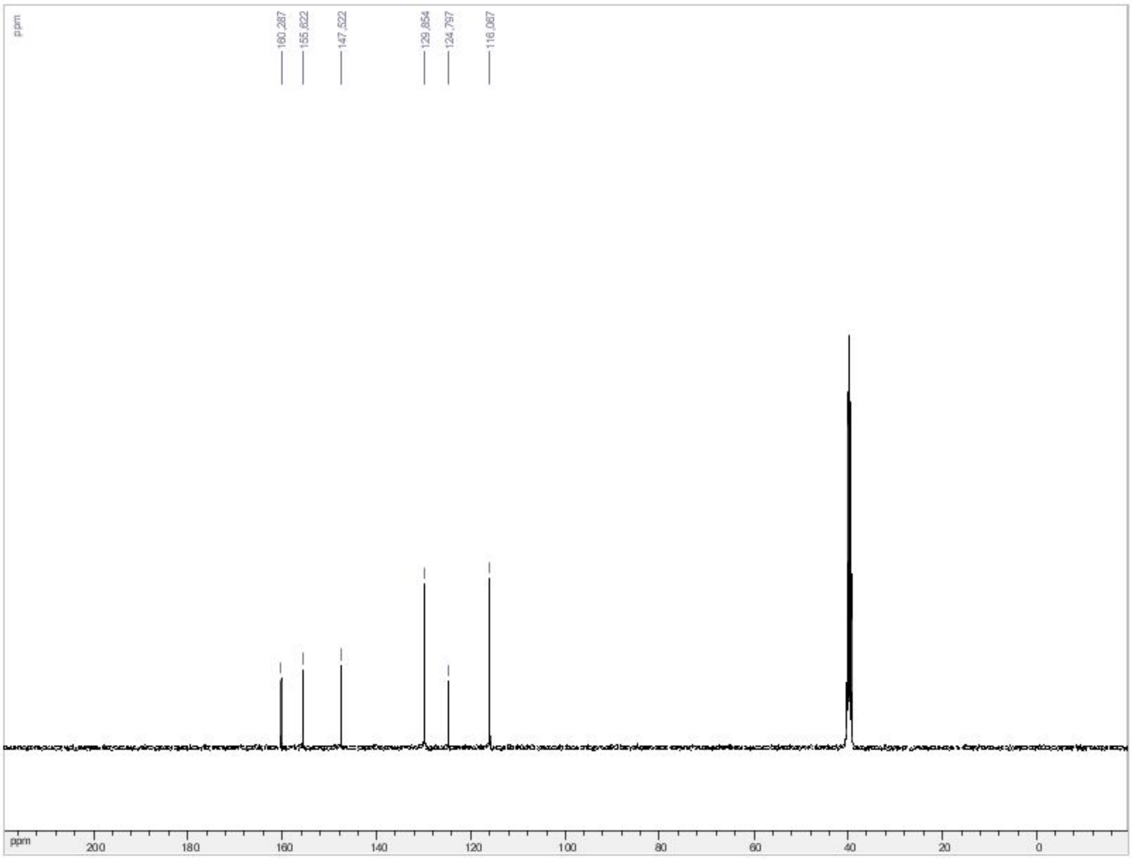

Figure S29. ^{13}C NMR (100Hz) in DMSO-d6 of LQM07.

LQM08:

0.2506 g of substance 8 (0.9029 mmol) was obtained with a yield of 83.28%.

^{1}H NMR (400 MHz, DMSO-d6): 7.63 (d, 2H, *J*=8.5); 7.81 (d, 2H, *J*=8.5); 8.17 (s, 1H). **^{13}C NMR** (100 MHz, DMSO-d6): 124.317; 129.934; 132.156; 133.159; 146.118; 155.797.

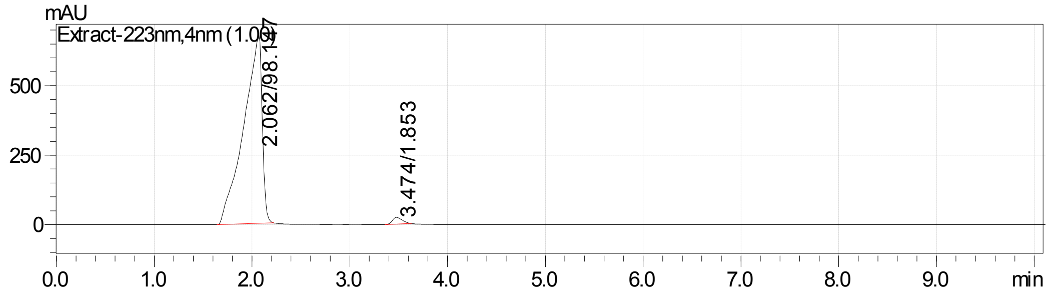

Figure S30. Chromatogram of substance LQM08

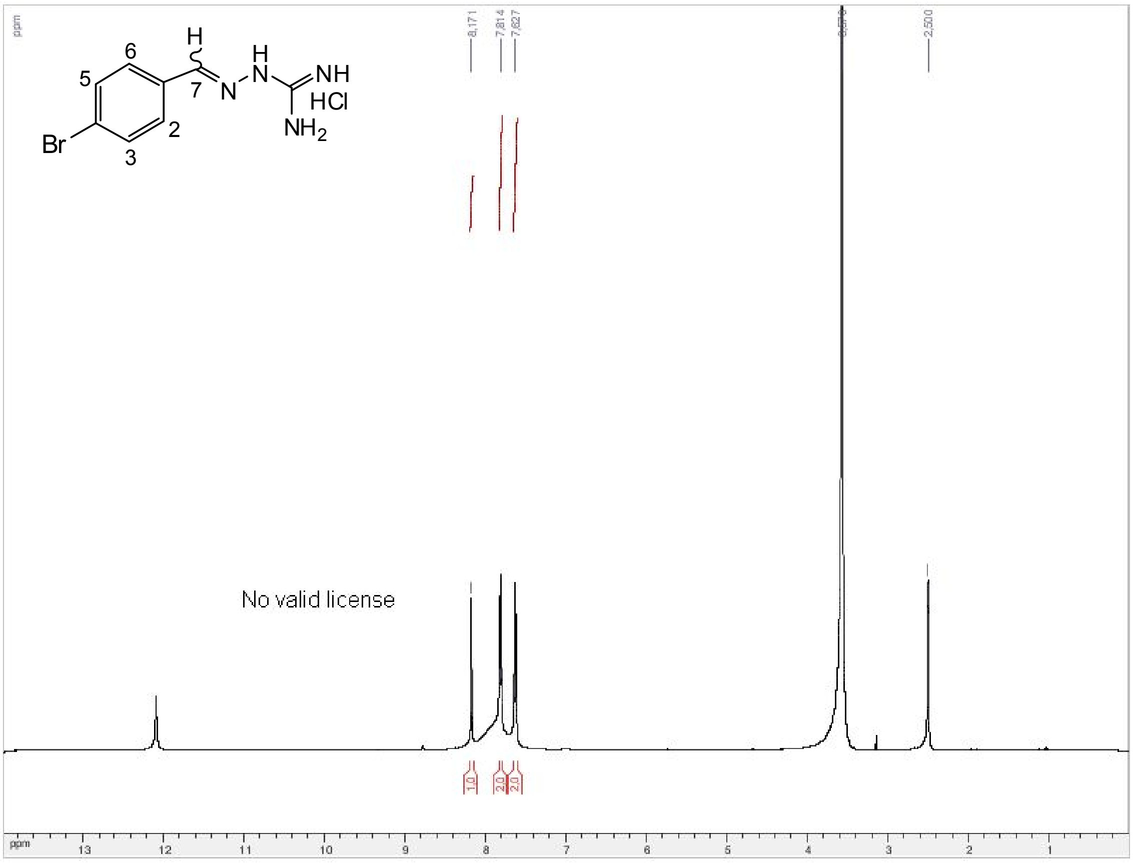

Figure S31. ^{1}H NMR (400Hz) in DMSO-*d6* of LQM08

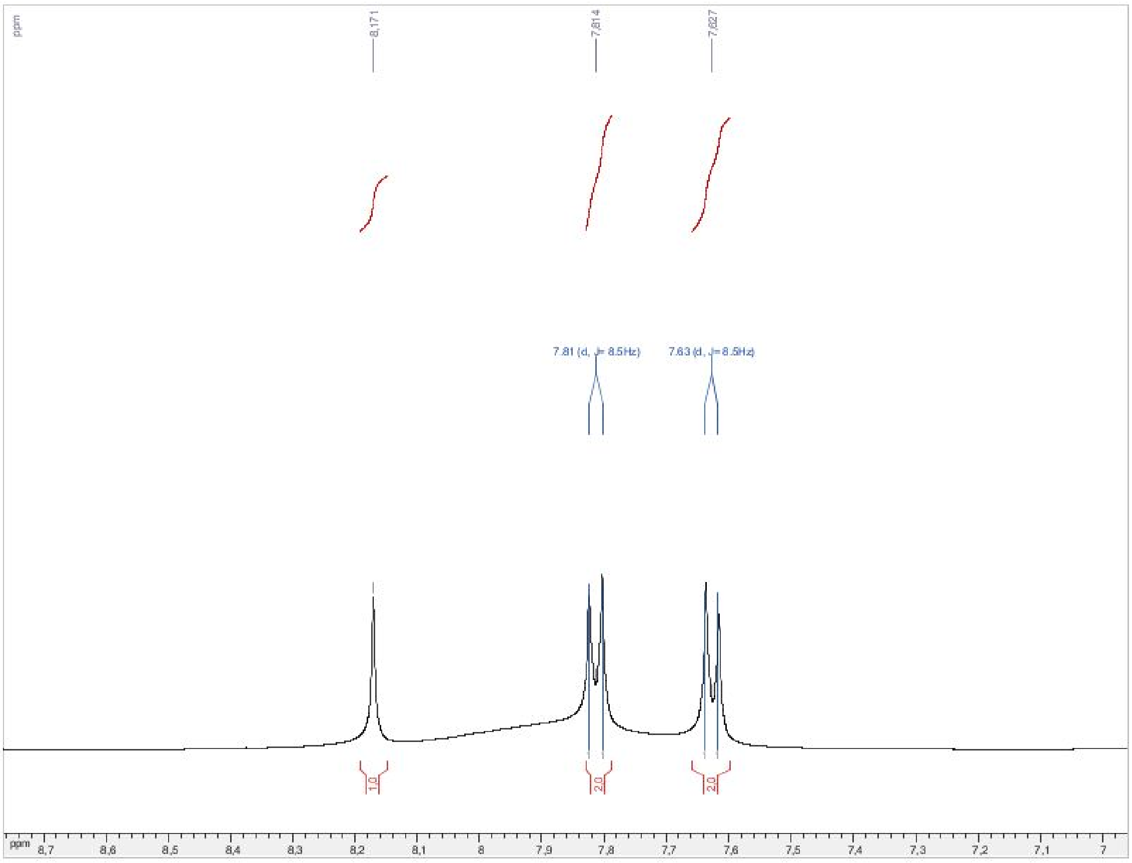

Figure S32. Enlargement of the spectrum from δ7.6 to δ8.4.

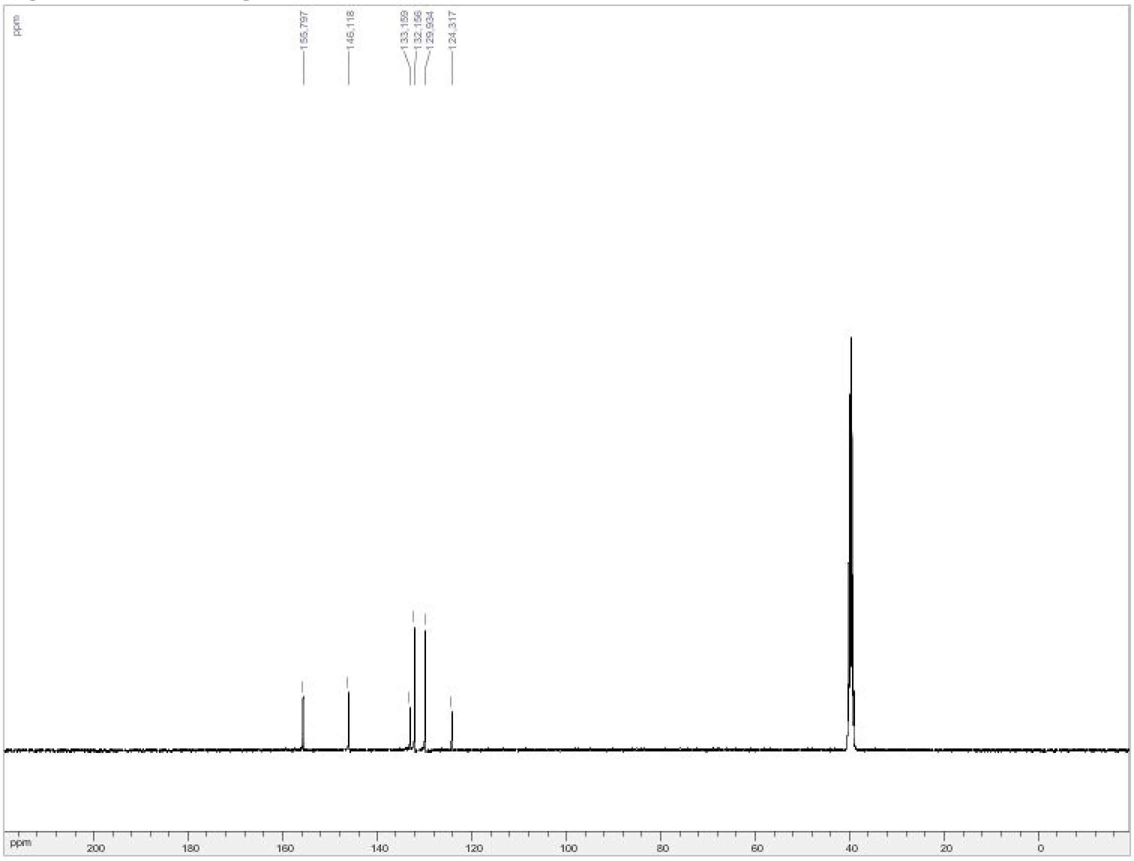

Figure S33. ^{13}C NMR (100Hz) in DMSO-*d6* of LQM08.

LQM09:

0.2632 g of substance 9 (1.0846 mmol) was obtained with a yield of 81.42%.

^{1}H NMR (400 MHz, DMSO-d6): 6.05 (s, 2H); 6.94 (d, 1H, *J*=8.0); 7.17 (dd, 1H, *J*=8.0 Hz and 1.6 Hz); 7.64 (d, 1H, *J*=1.6 Hz); 8.07 (s, 1H). **^{13}C NMR** (100 MHz, DMSO-d6): 102.032; 105.944; 108.657; 124.804; 128.378; 146.834; 148.456; 149.809; 155,746.

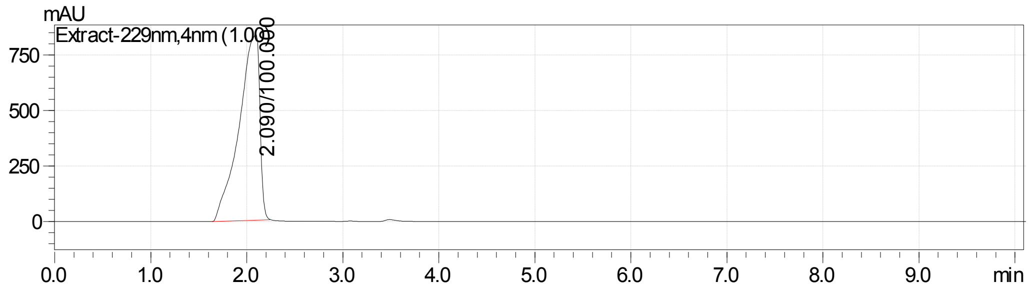

Figure S34. Chromatogram of substance LQM09

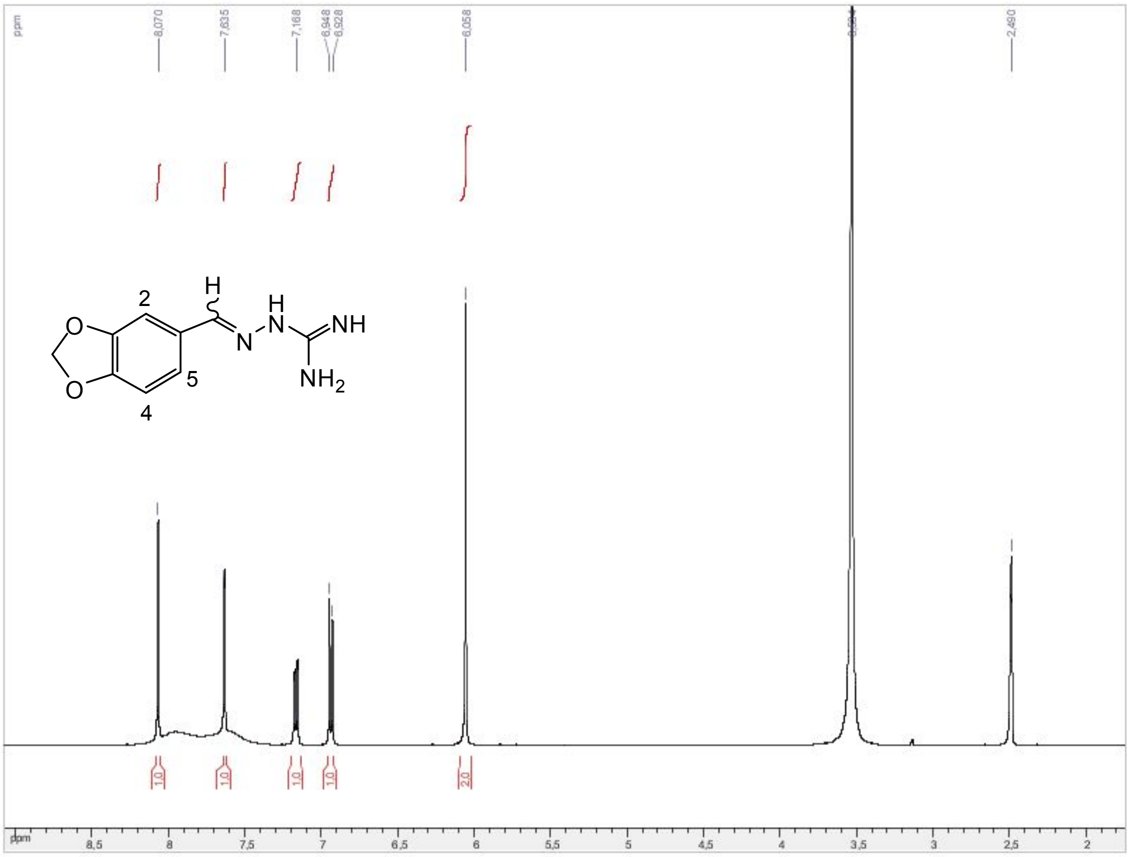

Figure S35. ^{1}H NMR (400Hz) in DMSO-d6 of LQM09.

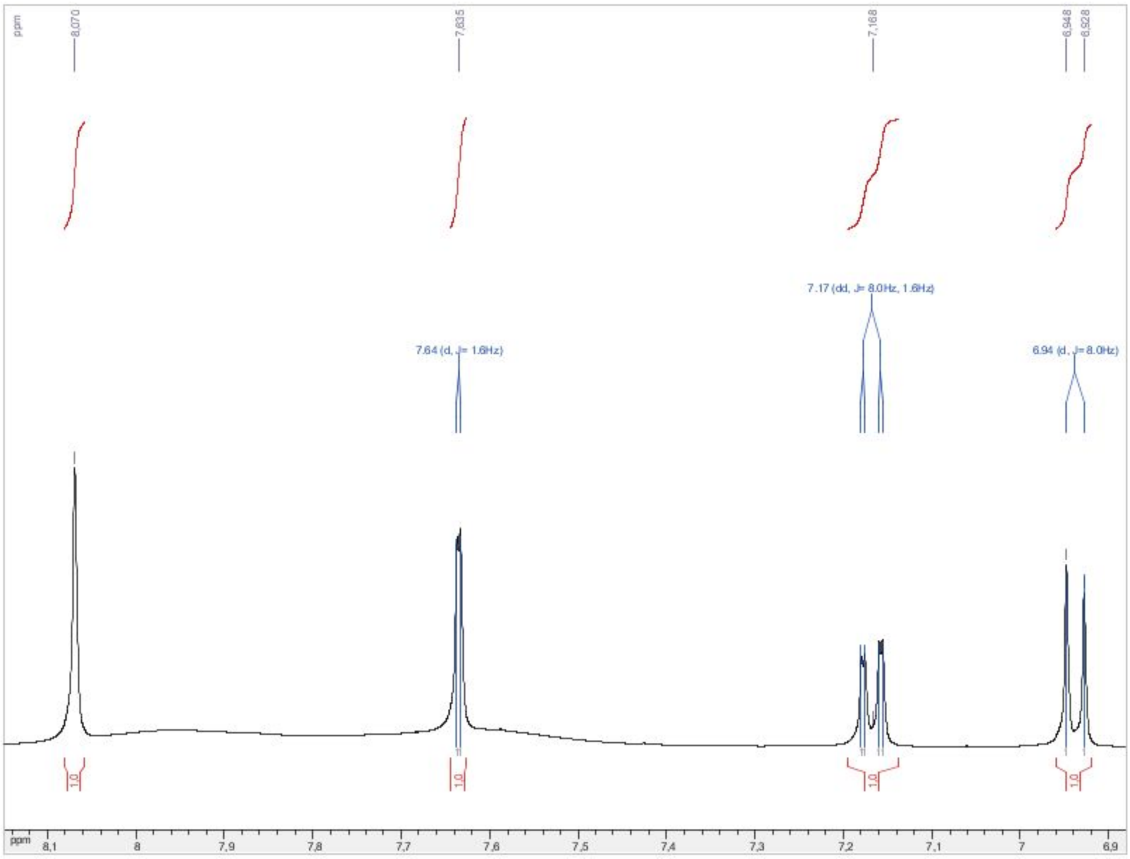

Figure S36. Enlargement of the spectrum from δ7.6 to δ8.4.

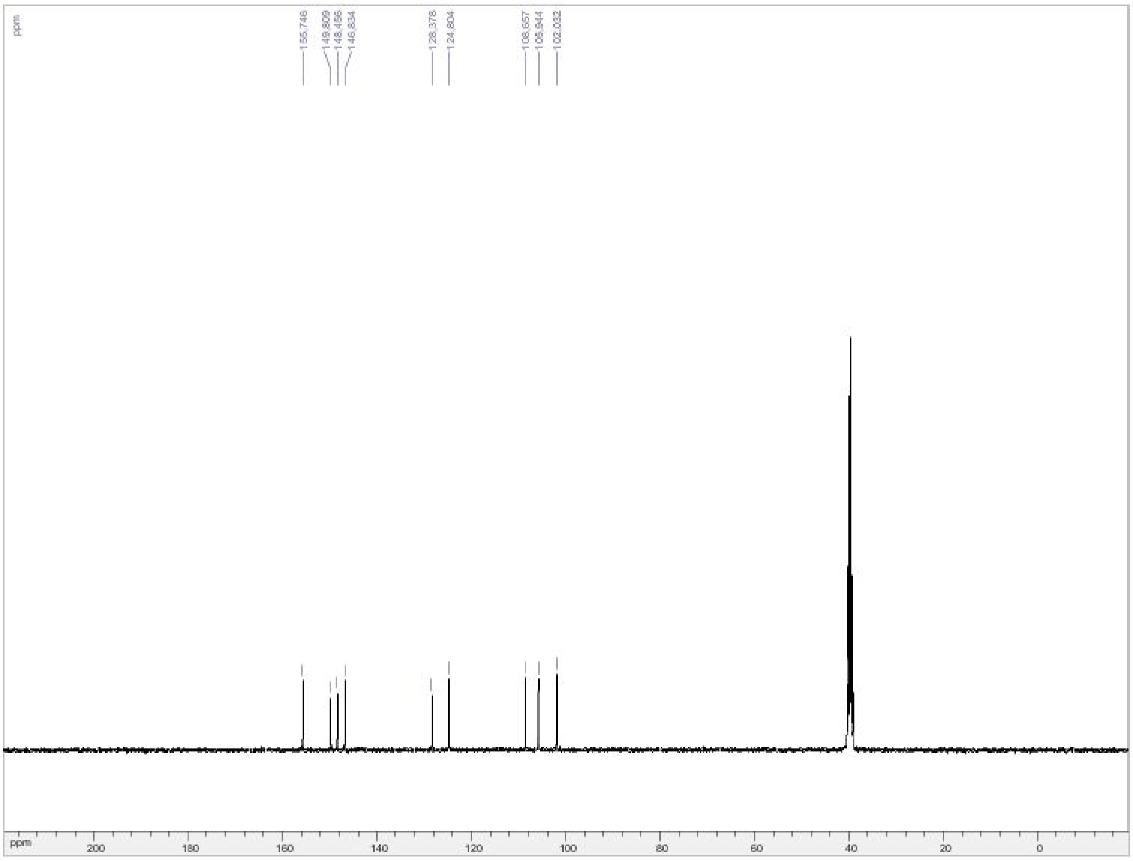

Figure S37. 13C NMR (100Hz) in DMSO-d6 of LQM09.

LQM10:

0.2379 g of substance 10 (0.7283 mmol) was obtained with a yield of 85.23%. **^{1}H NMR** (400 MHz, DMSO-d6): 1.38 (s, 18H); 7.50 (s, 2H); 8.09 (s, 1H). **^{13}C NMR** (100 MHz, DMSO-d6): 30.691; 35.018; 124.993; 139.661; 148.961; 155.532; 156.982.

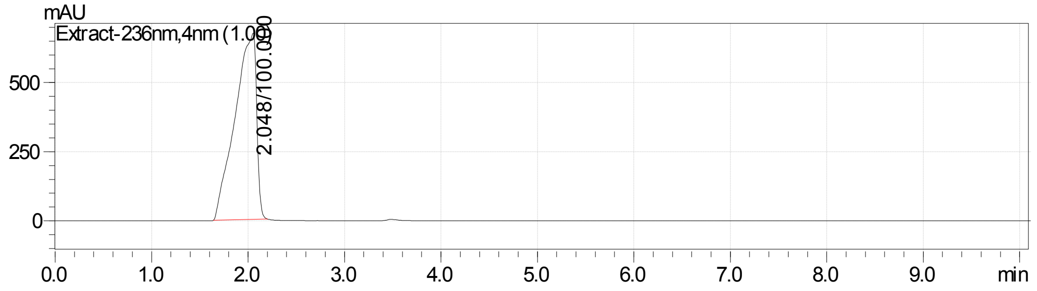

Figure S38. Chromatogram of substance LQM10

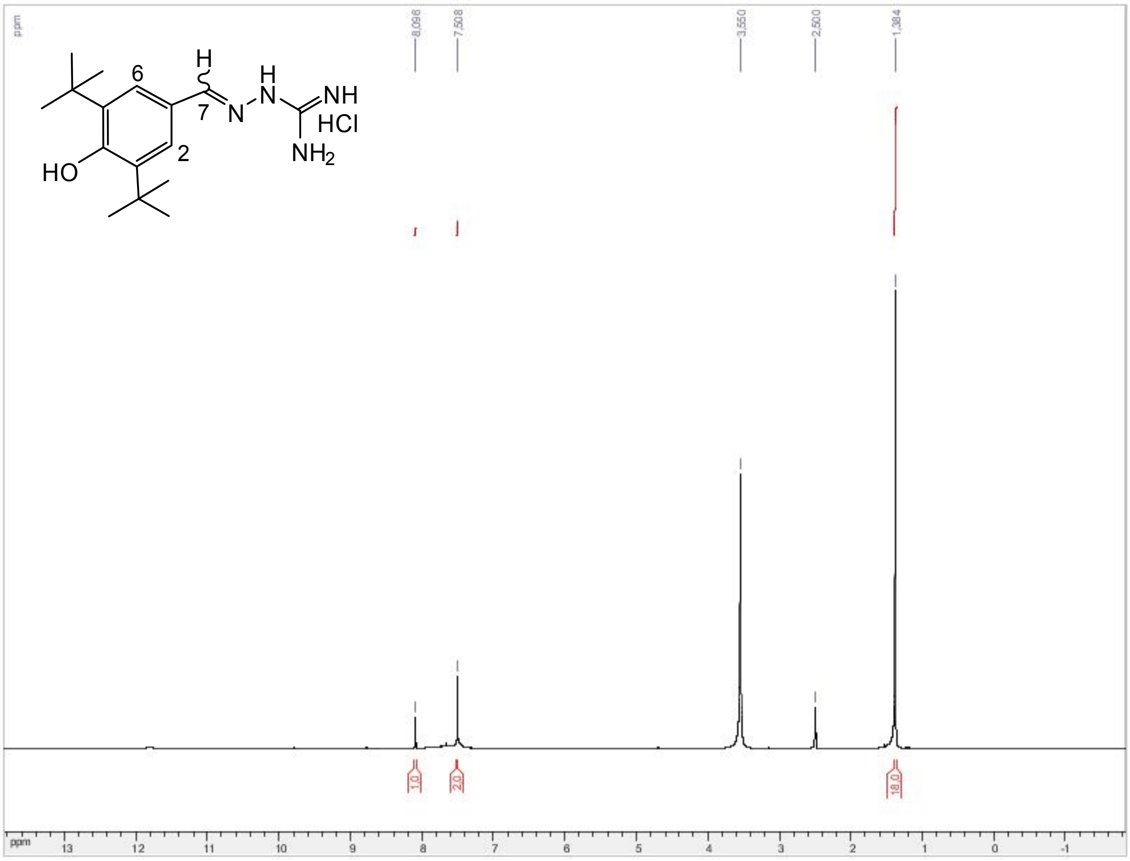

Figure S39. ^{1}H NMR (400Hz) in DMSO-d6 of LQM10.

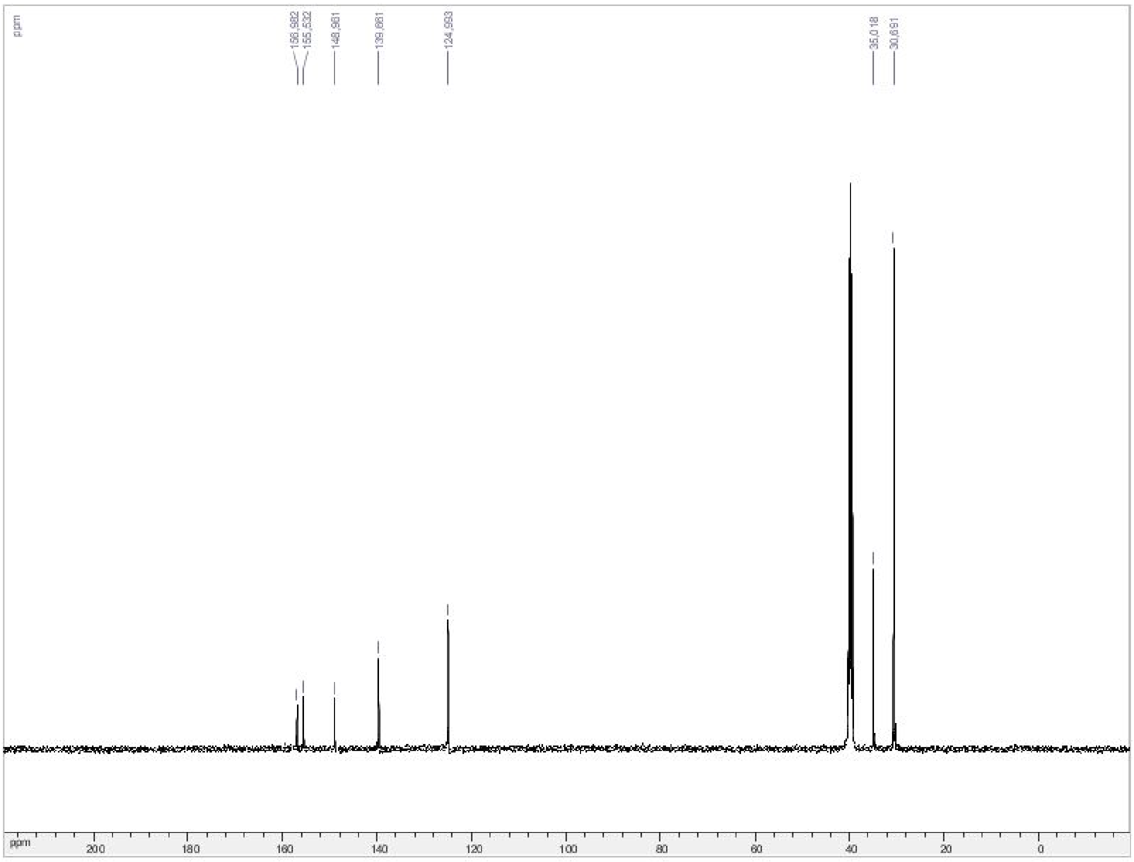

Figure S40. ^{13}C NMR (100Hz) in DMSO-d6 of LQM10.

LQM11:

0.2308 g of substance 11 (0.9486 mmol) was obtained in 71.21% yield. 1H NMR (100 MHz, DMSO-d6): 7.90 (s, 4H); 8.20 (s, 1H). 13C NMR (100 MHz, DMSO-d6): 128.073; 130.011, 132.458, 137.868; 146.18; 155.848; 167.367.

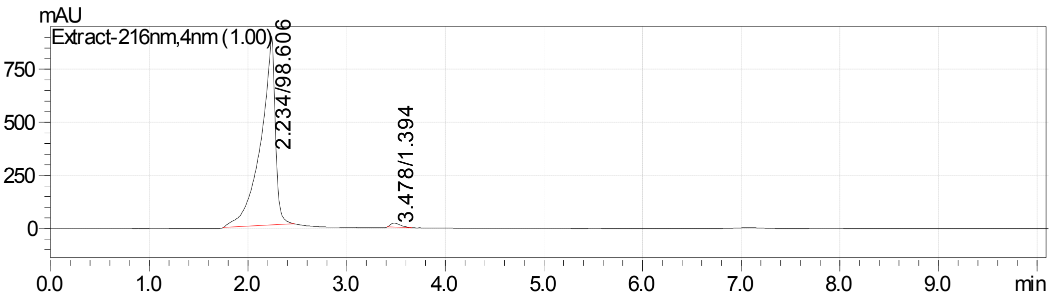

Figure S41. Chromatogram of substance LQM11

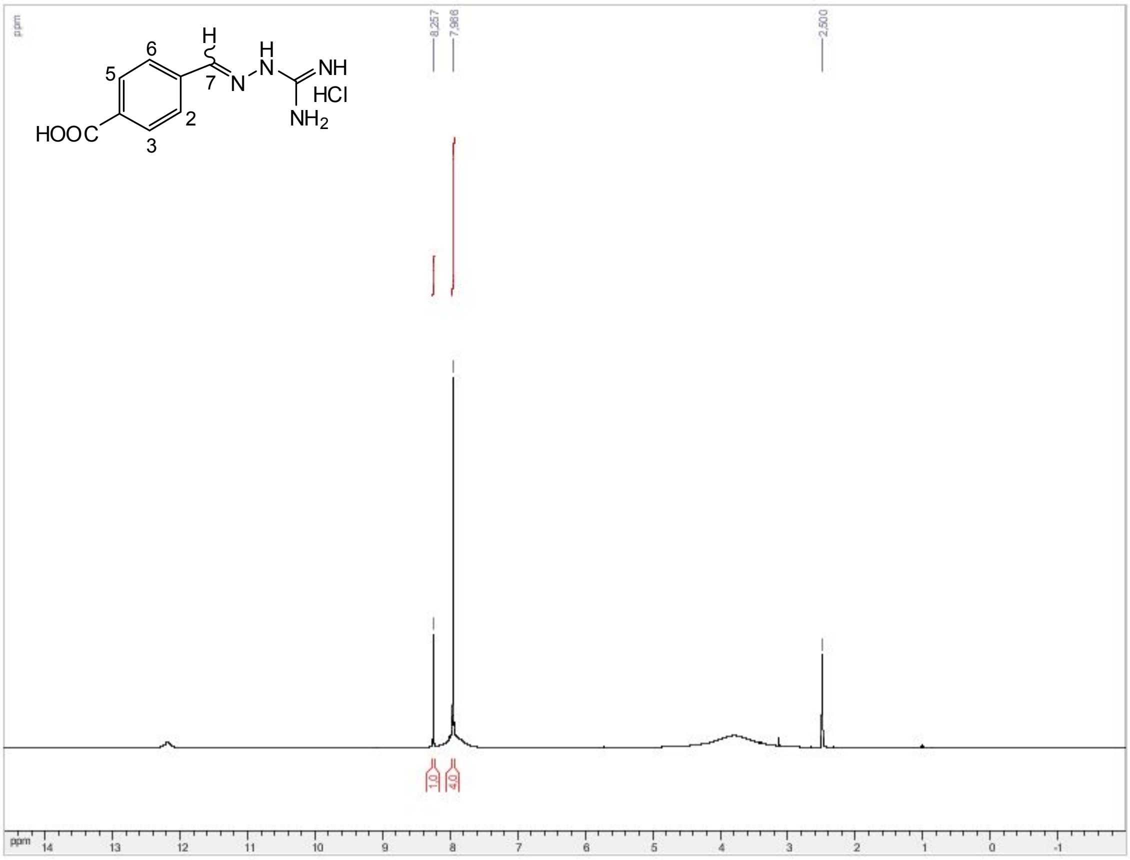

Figure S42. ^{1}H NMR (400Hz) in DMSO-d6 of LQM11.

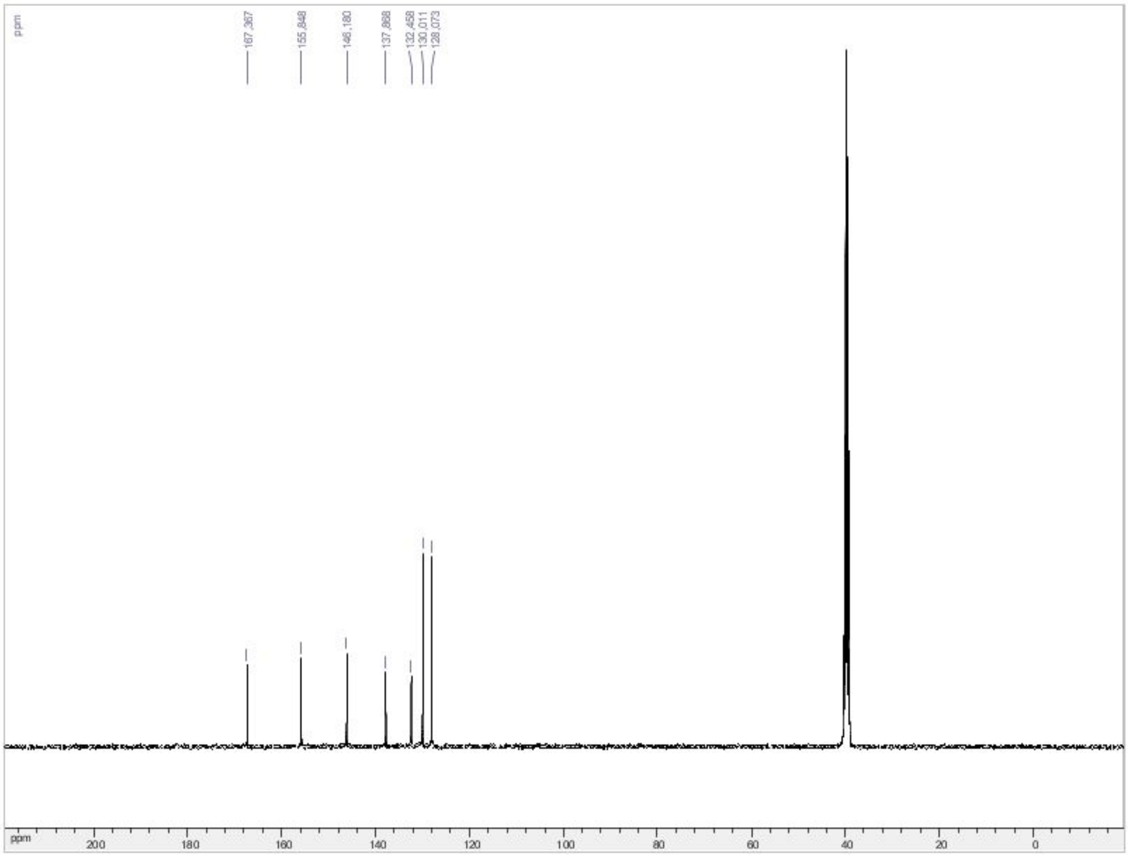

Figure S43. ^{13}C NMR (100Hz) in DMSO-*d6* of LQM11.

LQM12:

0.2725 g of substance 12 (1.0185 mmol) was obtained with a yield of 89.12%. **^{1}H NMR** (400 MHz, DMSO-d6): 7.45 (m, 1H); 7.57 (d, 2H, J=7.9); 8.42 (s, 1H). **^{13}C NMR** (100 MHz, DMSO-d6): 129.564; 130.011; 132.185; 134.53; 142.998; 155.786.

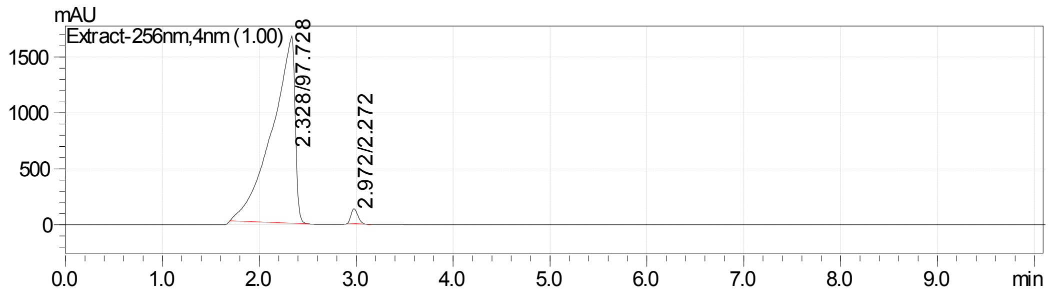

Figure S44, Chromatogram of substance LQM12

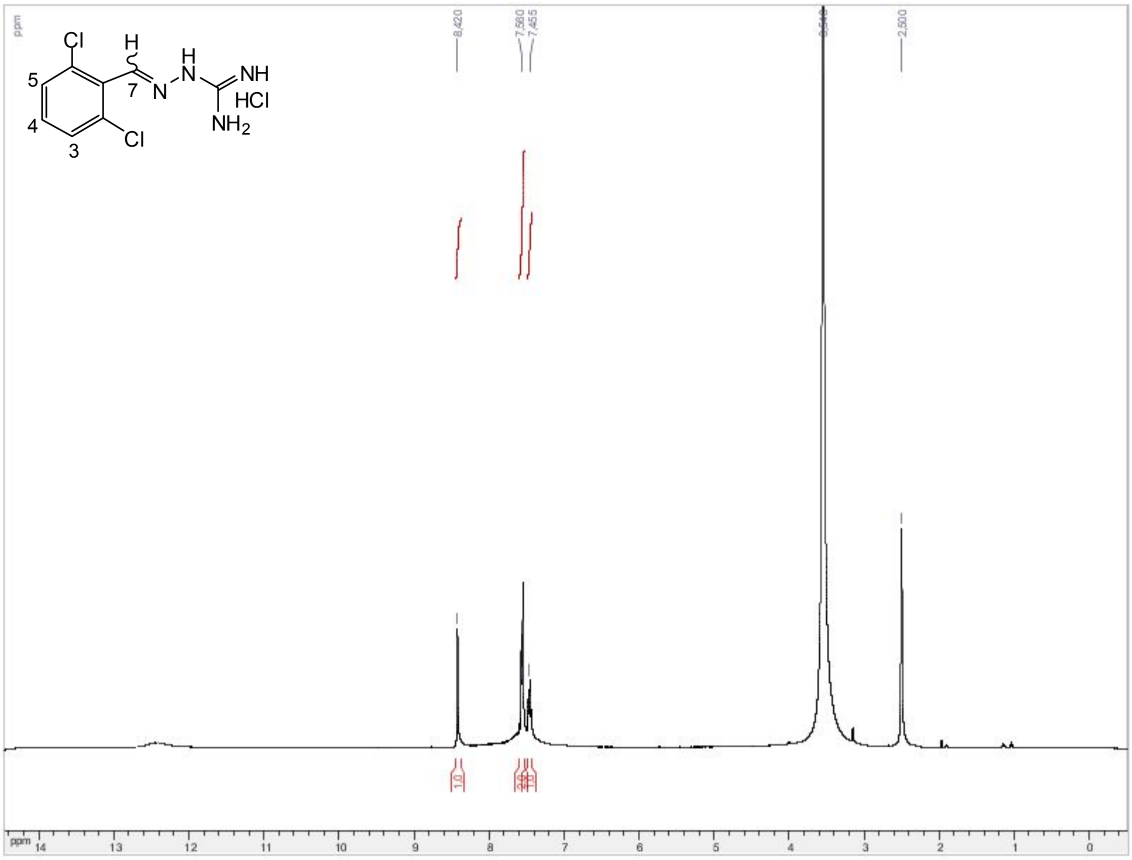

Figure S45. ^{1}H NMR (400Hz) spectrum in DMSO-d6 of LQM12.

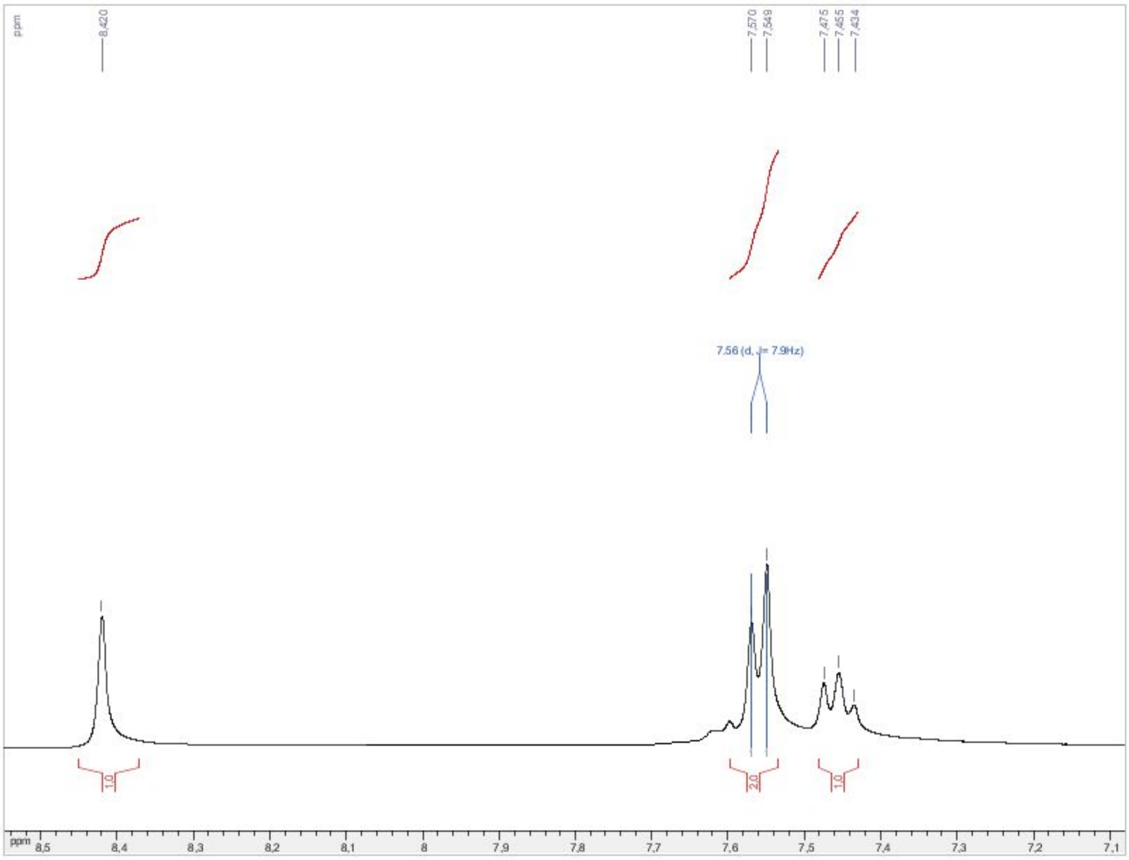

Figure S46. Enlargement of the spectrum from δ6.8 to δ8.6.

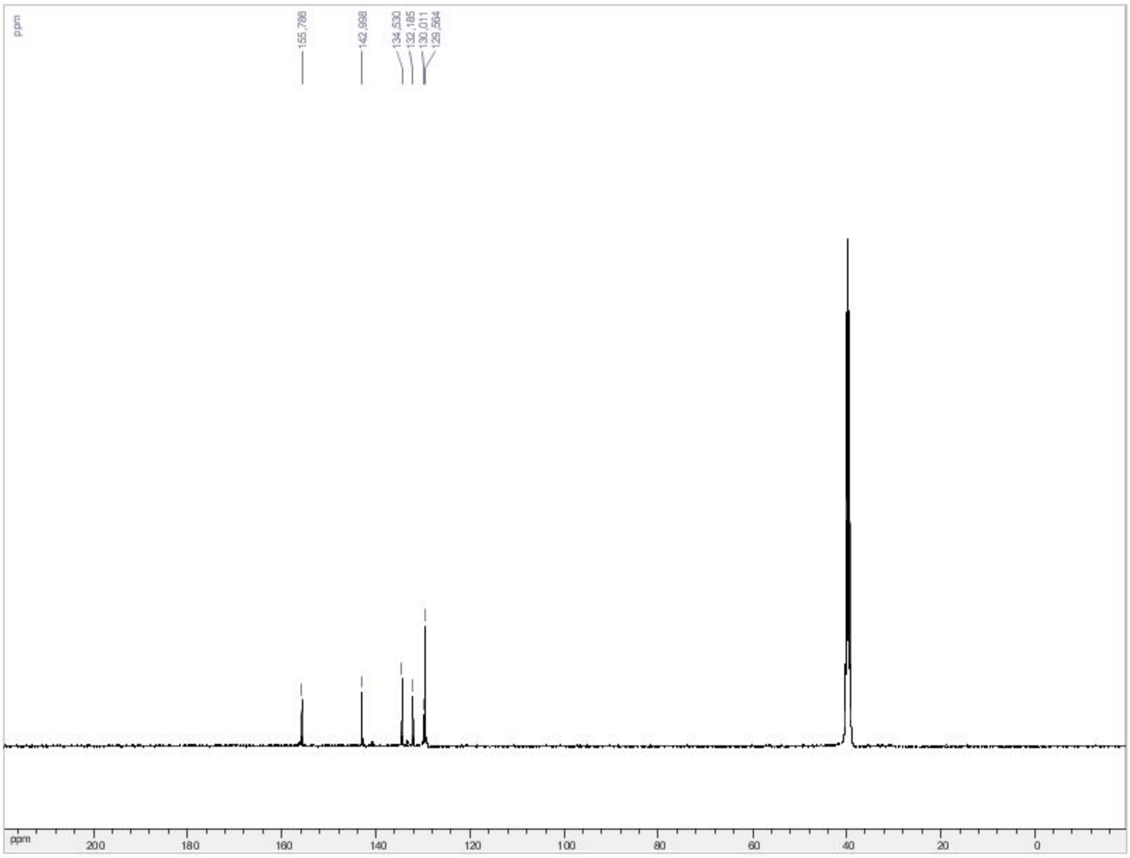

Figure S47. ^{13}C NMR (100Hz) in DMSO-*d6* of LQM12.

LQM13:

0.2808 g of substance 13 (1.1534 mmol) was obtained with a yield of 90.64%. ^{1}**H NMR** (400 MHz, DMSO-d6): 7.72 (t, 1H, J=7.5); 7.84 (t, 1H, J=7.5); 8.09 (d, 1H, J=8.2); 8.40 (d, 1H, J=8.2); 8.18 (d, 1H, J=4.3); 8.98 (d, 1H, J=4.3); 9.05 (s, 1H). ^{13}**C NMR** (100 MHz, DMSO-d6): 119.081; 123.891; 125.313; 128.127; 130.149; 130,291; 137,097; 143,478; 148,7; 150,616; 155,775.

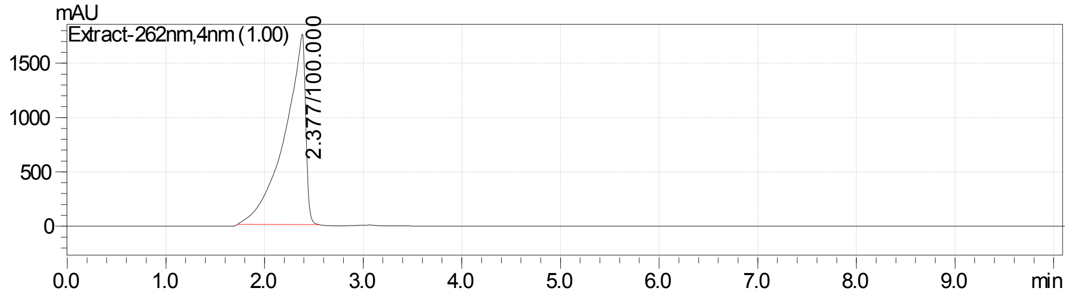

Figure S48. Chromatogram of substance LQM13

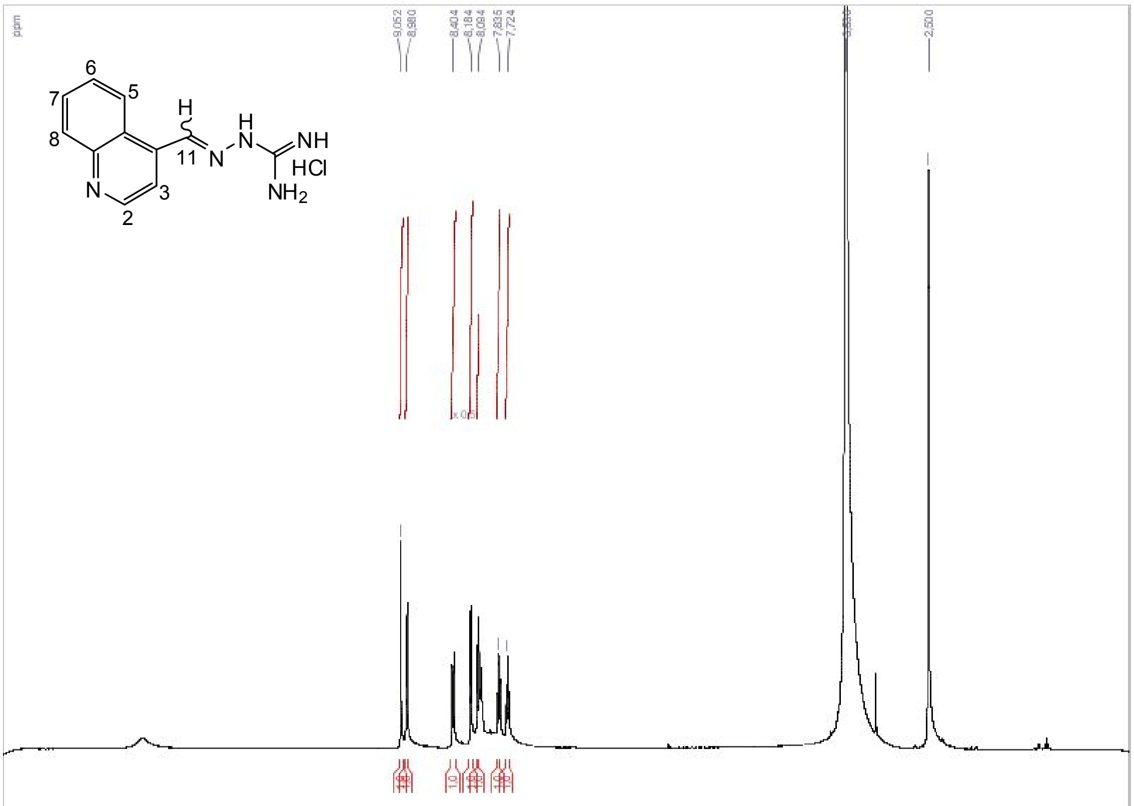

Figure S49. ^{1}H NMR (400Hz) in DMSO-d6 of LQM13.

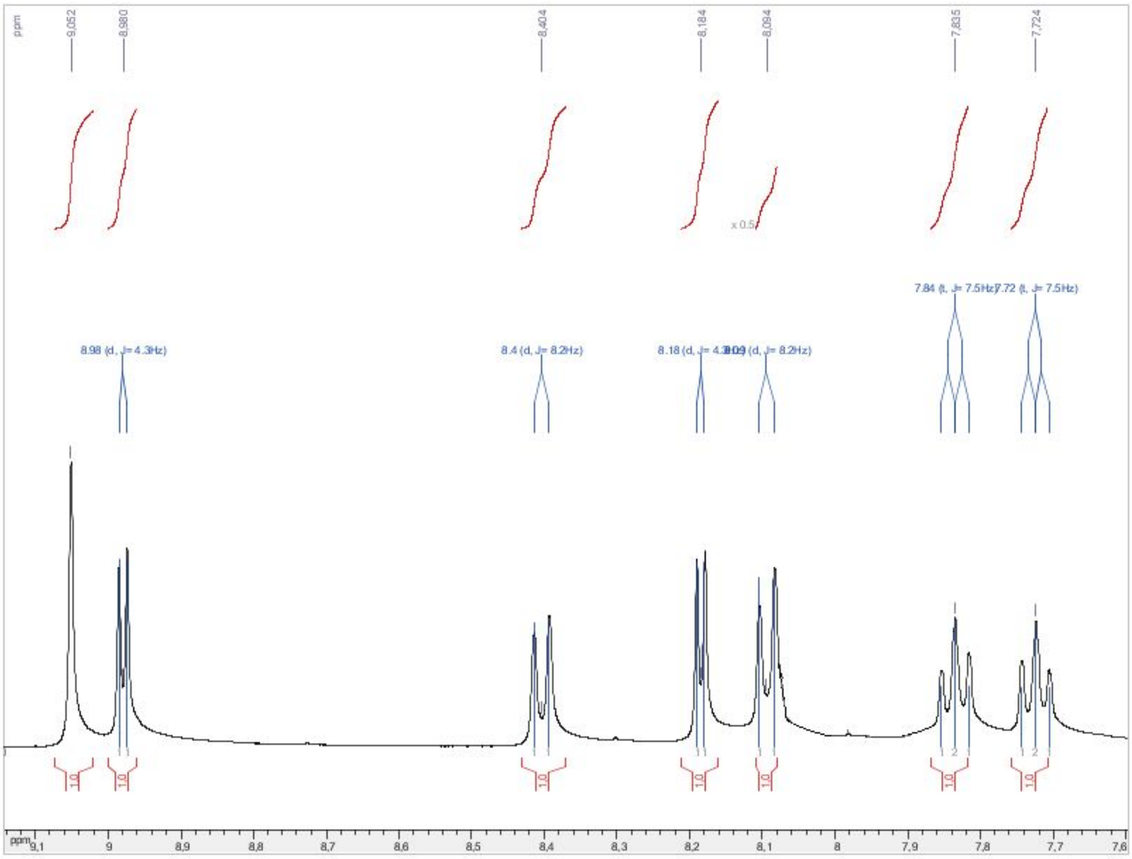

Figure S50. Enlargement of the spectrum from δ6.8 to δ8.6.

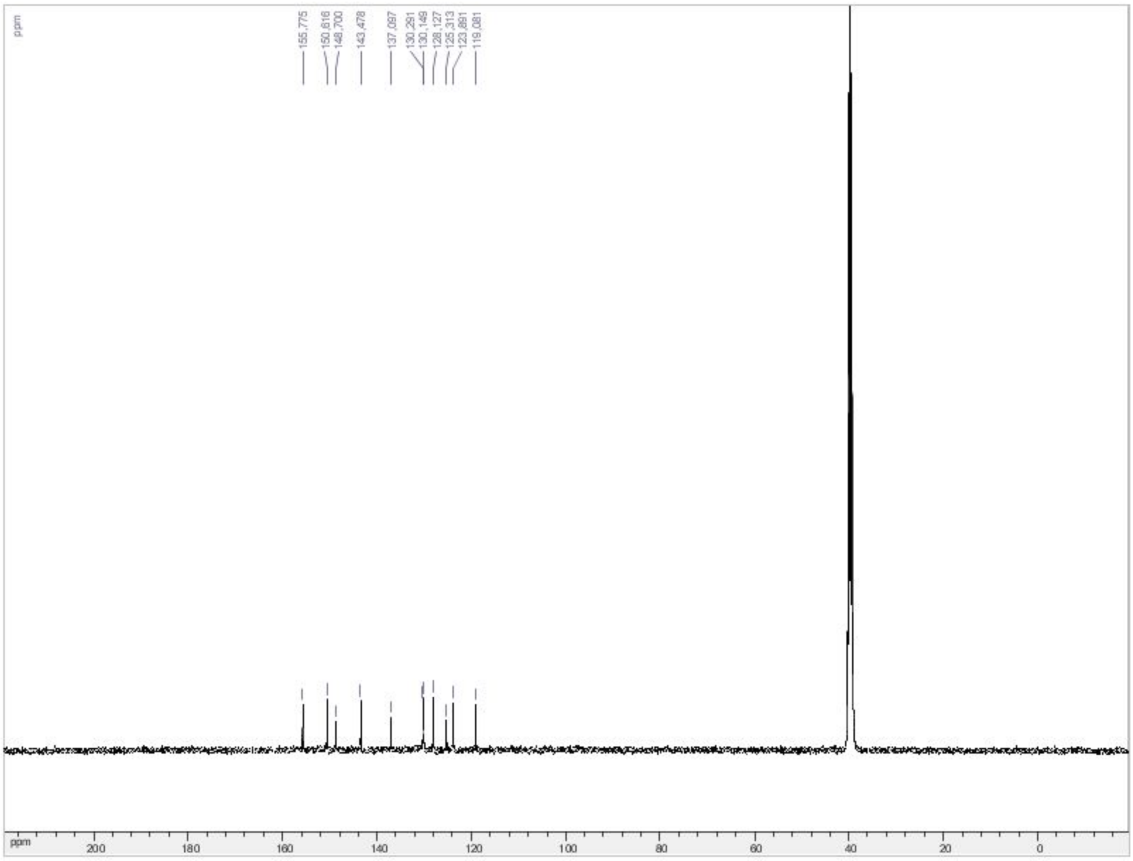

Figure S51. ^{13}C NMR (100Hz) in DMSO-d6 of LQM13.

LQM14:

0.2706g of substance 14 (0.9849 mmol) was obtained with a yield of 89.73%. **^{1}H NMR** (400 MHz, DMSO-d6): 7.47 (m, 5H); 7.74 (d, 2H, J=8.4); 7.94 (d, 2H, J=8.4); 8.24 (s, 1H). **^{13}C NMR** (100 MHz, DMSO-d6): 127.2; 127,357; 128,447; 128,684; 129,516; 132,945; 139,682; 142,413; 146,958; 155.79.

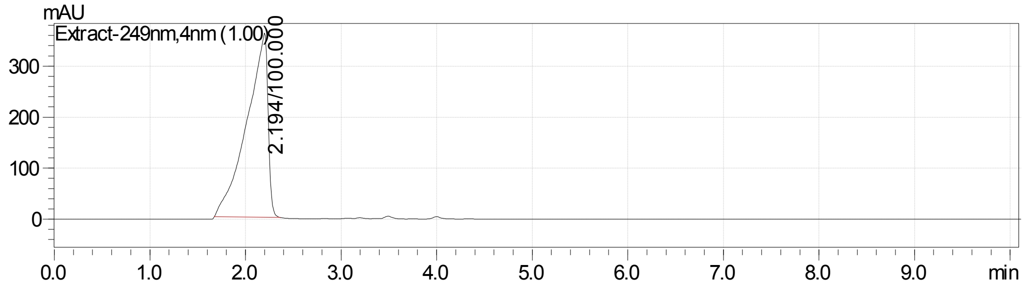

Figure S52. Chromatogram of substance LQM14

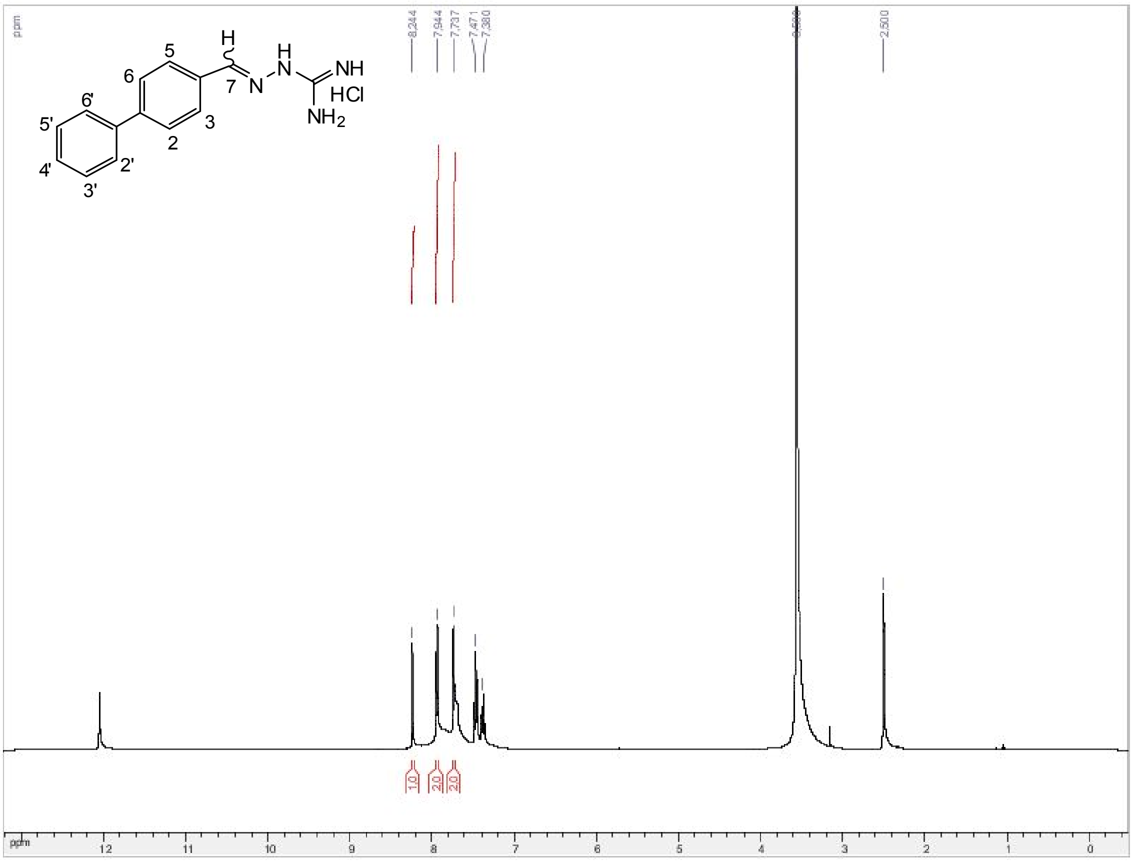

Figure S53. ^{1}H NMR (400Hz) spectrum in DMSO-*d6* of LQM14.

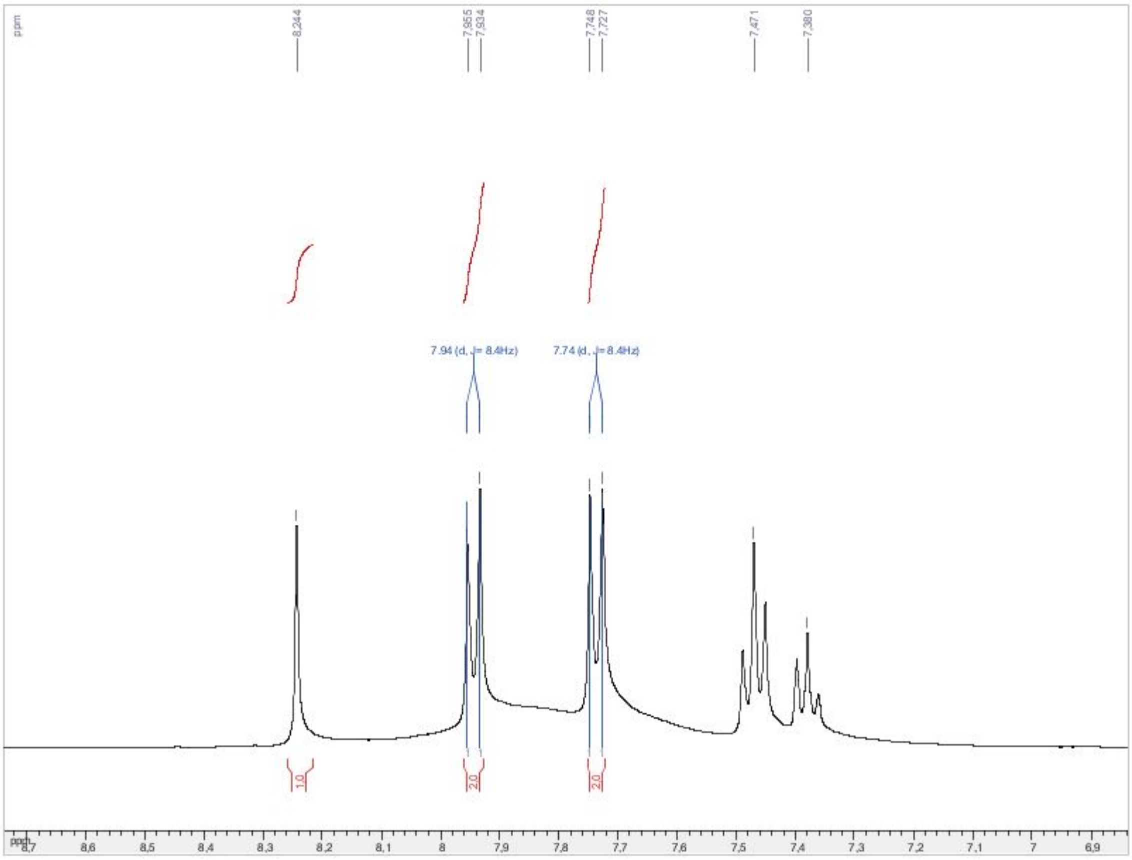

Figure S54. Enlargement of the spectrum from δ6.8 to δ8.6.

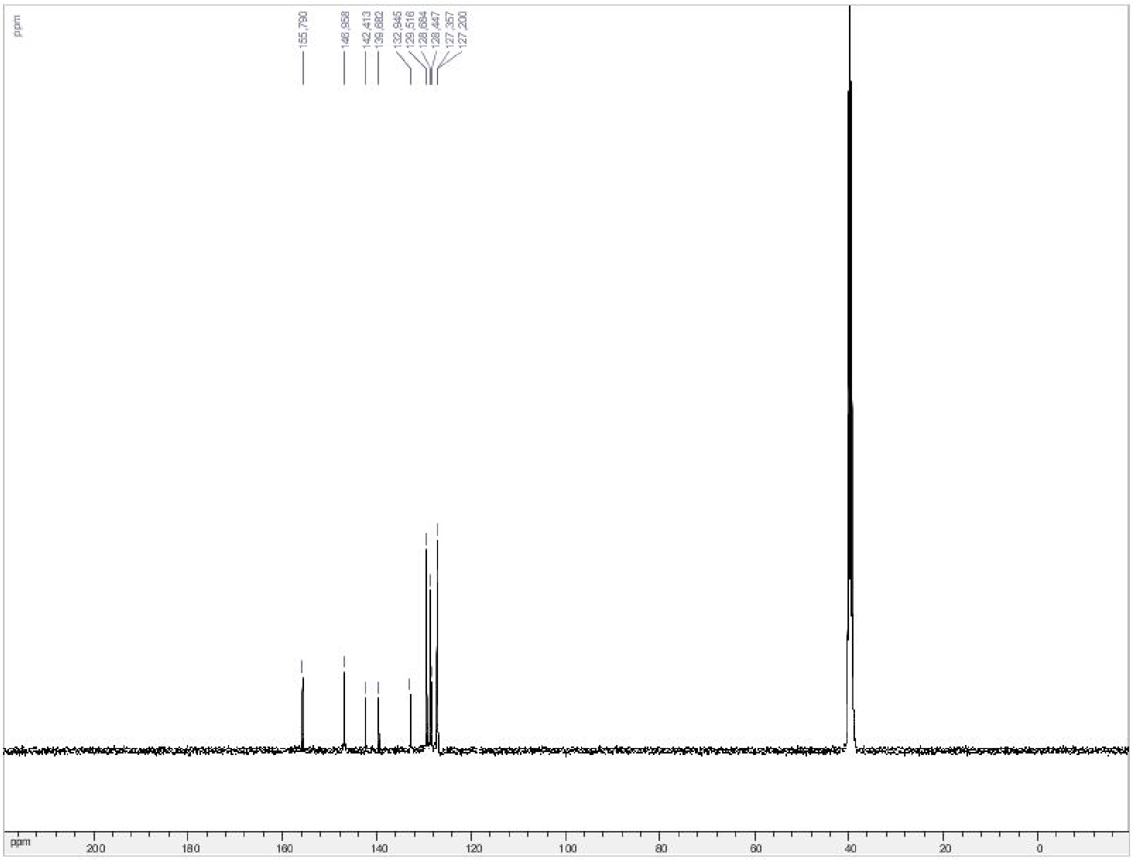

Figure S55. ^{13}C NMR (100Hz) in DMSO-*d6* of LQM14.

LQM15:

0.3333 g of substance 15 (1.6777 mmol) was obtained in 89.02% yield. **^{1}H NMR** (400 MHz, DMSO-d6): 7.43 (m, 3H); 7.84 (m, 2H); 8.19 (s, 1H). **^{13}C NMR** (100 MHz, DMSO-d6): 128.033; 129.178; 130.996; 133.818; 147.351; 155.801.

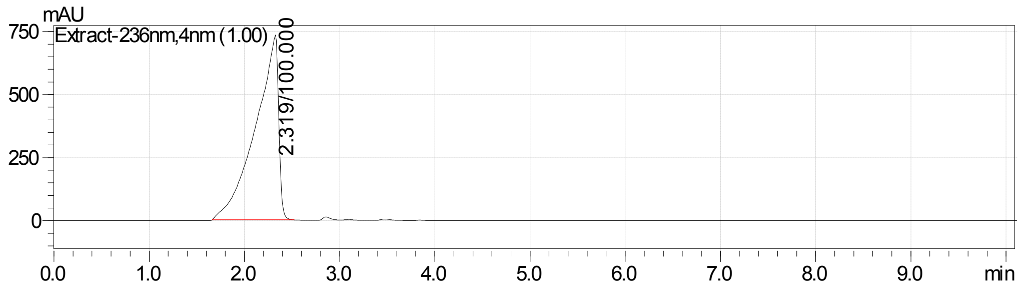

Figure S56. Chromatogram of substance LQM15

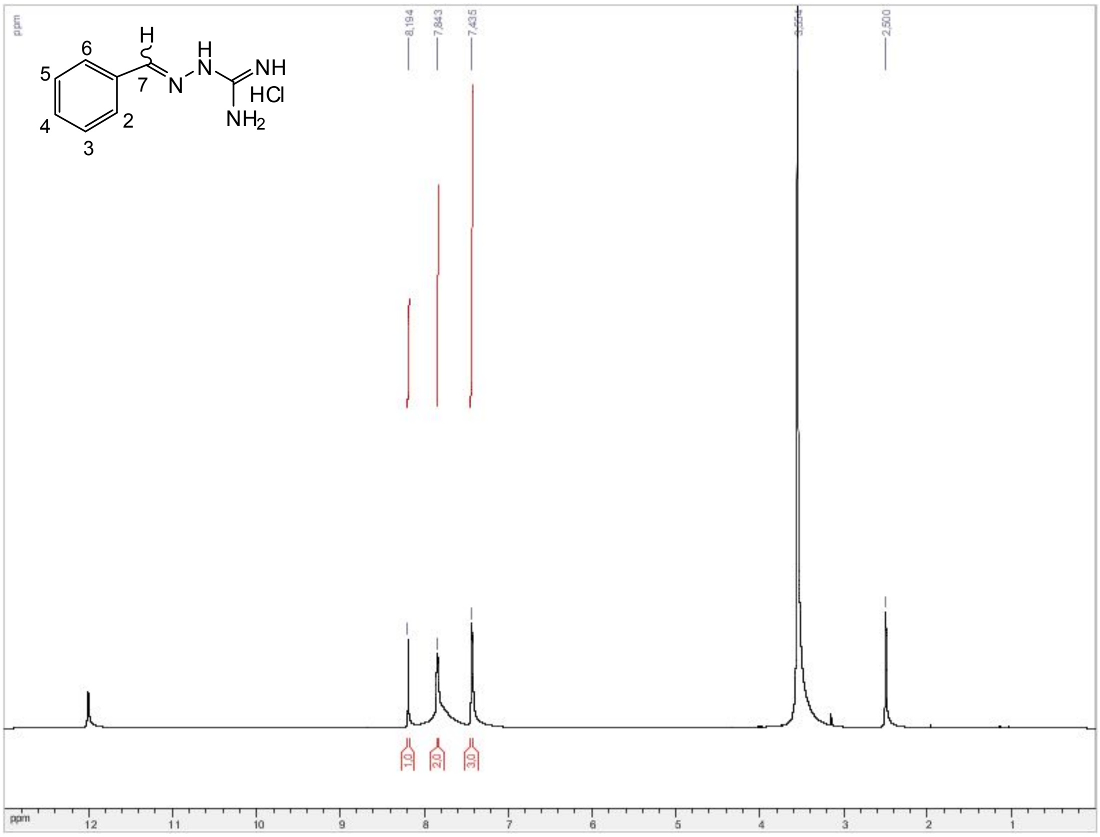

Figure S57. 1H NMR (400Hz) in DMSO-*d6* of LQM15.

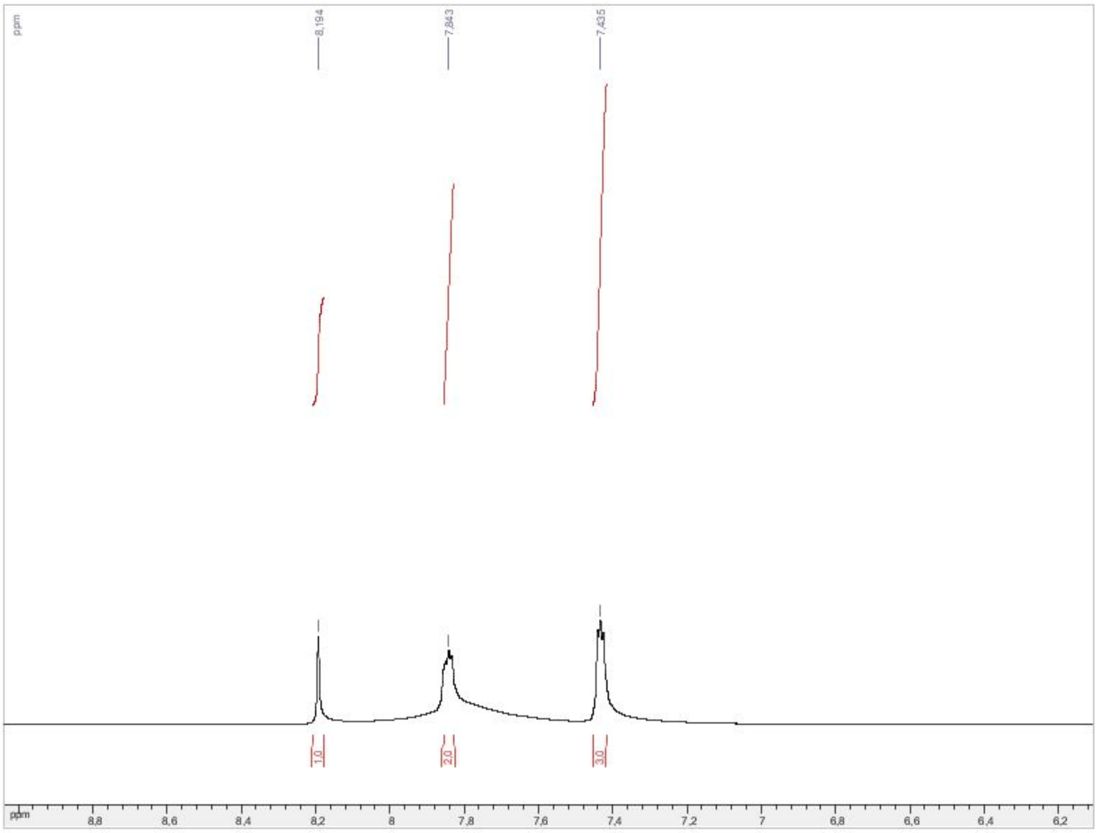

Figure S58. Enlargement of the spectrum from δ6.2 to δ8.8.

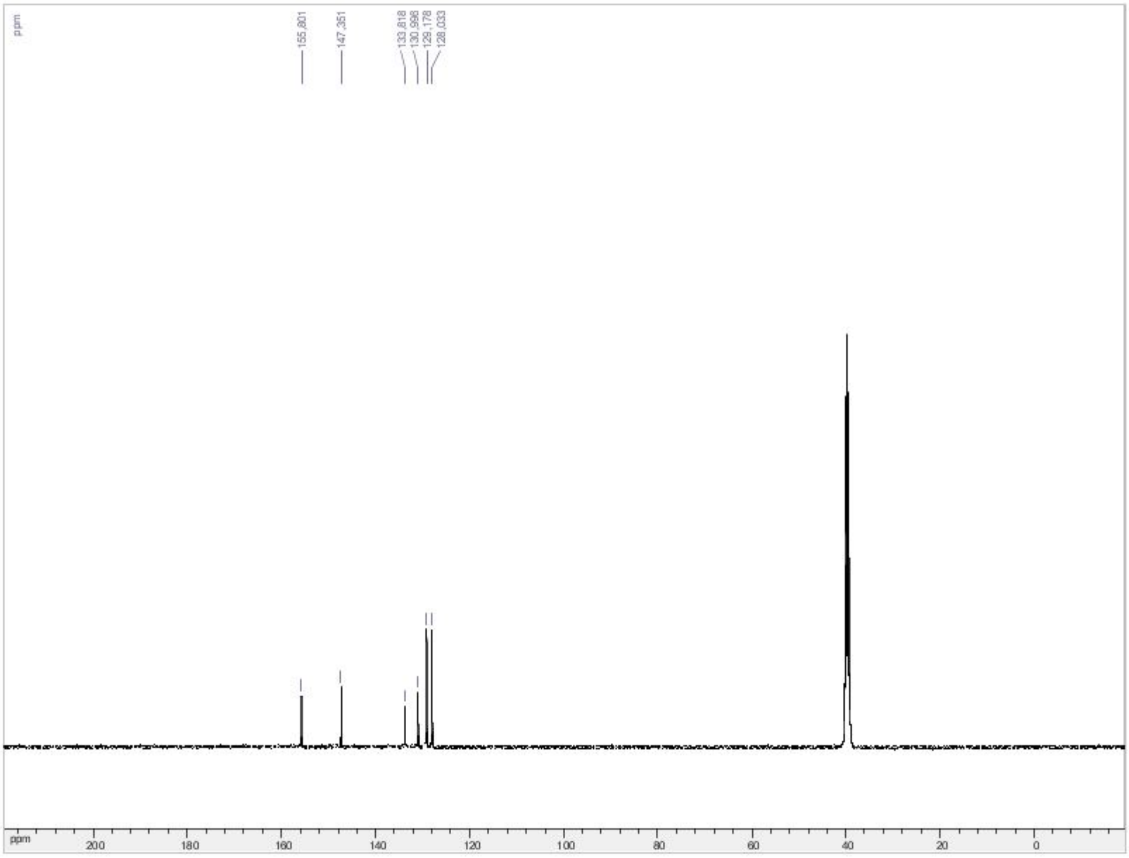

Figure S59. ^{1}H NMR (400Hz) in DMSO-*d6* of LQM15.

LQM16:

0.2885 g of substance 16 (1.12397 mmol) was obtained in 92.25% yield. ^{1}**H NMR** (400 MHz, DMSO-d6): 3.84 (s, 3H); 7.98 (s, 4H); 8.25 (s, 1H). ^{13}**C NMR** (100 MHz, DMSO-d6): 52.798; 128.196; 129.873; 131.214; 138.253; 146.082; 155.826; 166.308.

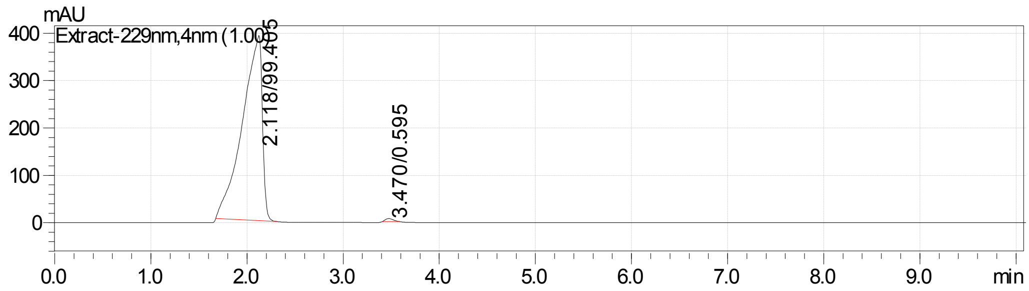

Figure S60. Chromatogram of substance LQM16

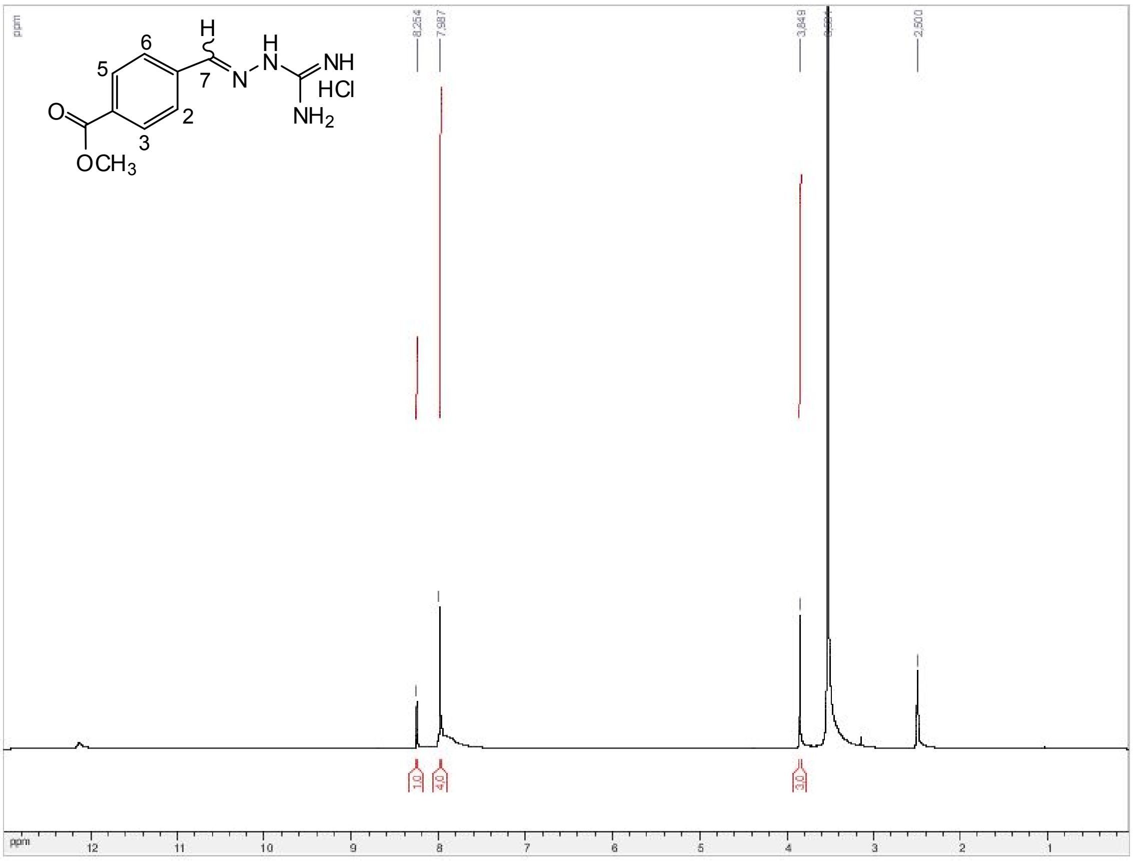

Figure S61. 1H NMR (400Hz) spectrum in DMSO-d6 of LQM16.

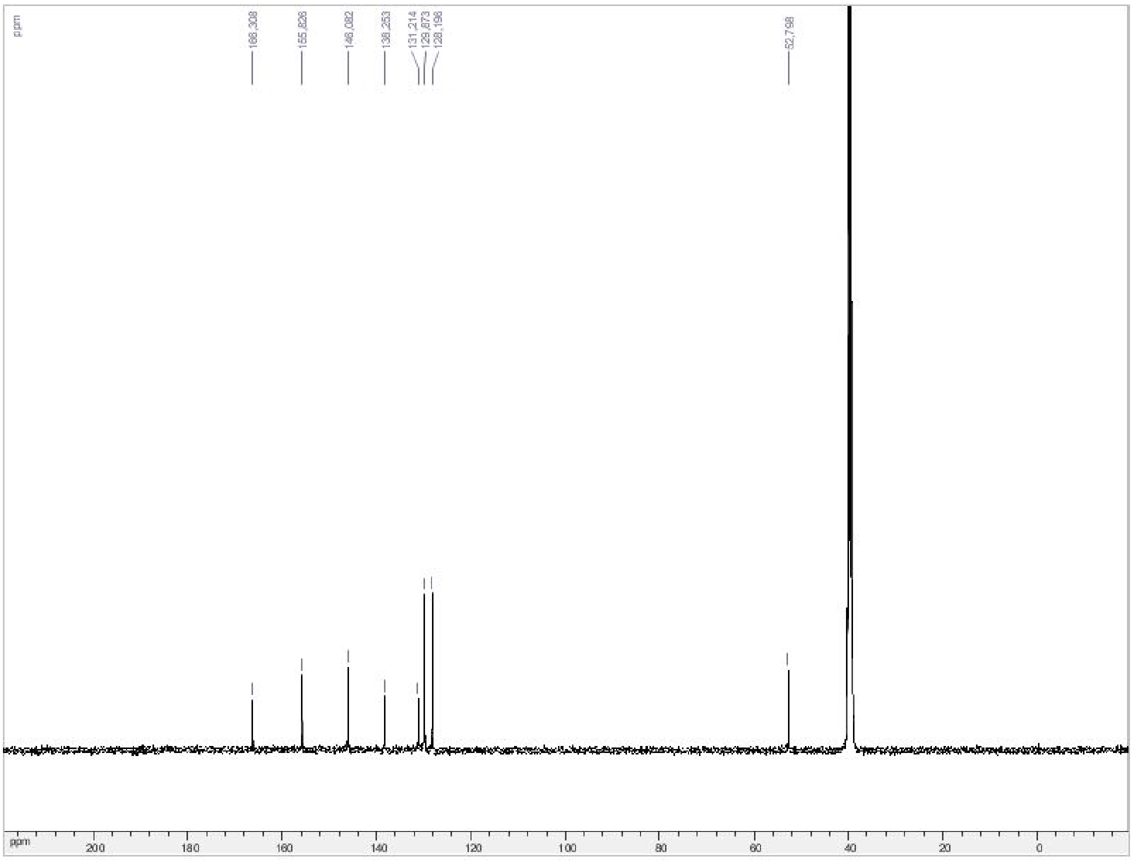

Figure S62. ^{13}C NMR (100Hz) in DMSO-*d6* of LQM16.

LQM17:

It was filtered and washed with AcOEt. 0.2823g of substance 17 (1.0551 mmol) was obtained with a yield of 92.33%. **^{1}H NMR** (400 MHz, DMSO-d6): 7.68 (d, 1H, J=8.4); 7.79 (dd, 1H, J=8.4 and 2.0); 8.24 (d, 1H, J=2.0); 8.16 (s, 1H). **^{13}C NMR** (100 MHz, DMSO-d6): 128.451; 129.025; 131.338; 132.254; 133.13; 134.661; 144.751; 155.819.

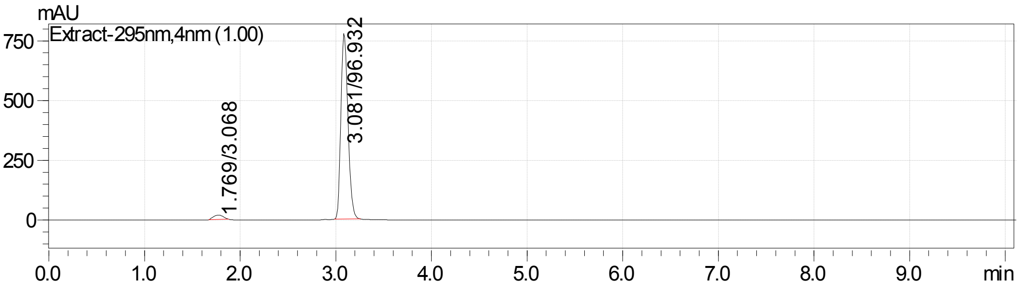

Figure S62. Chromatogram of substance LQM17

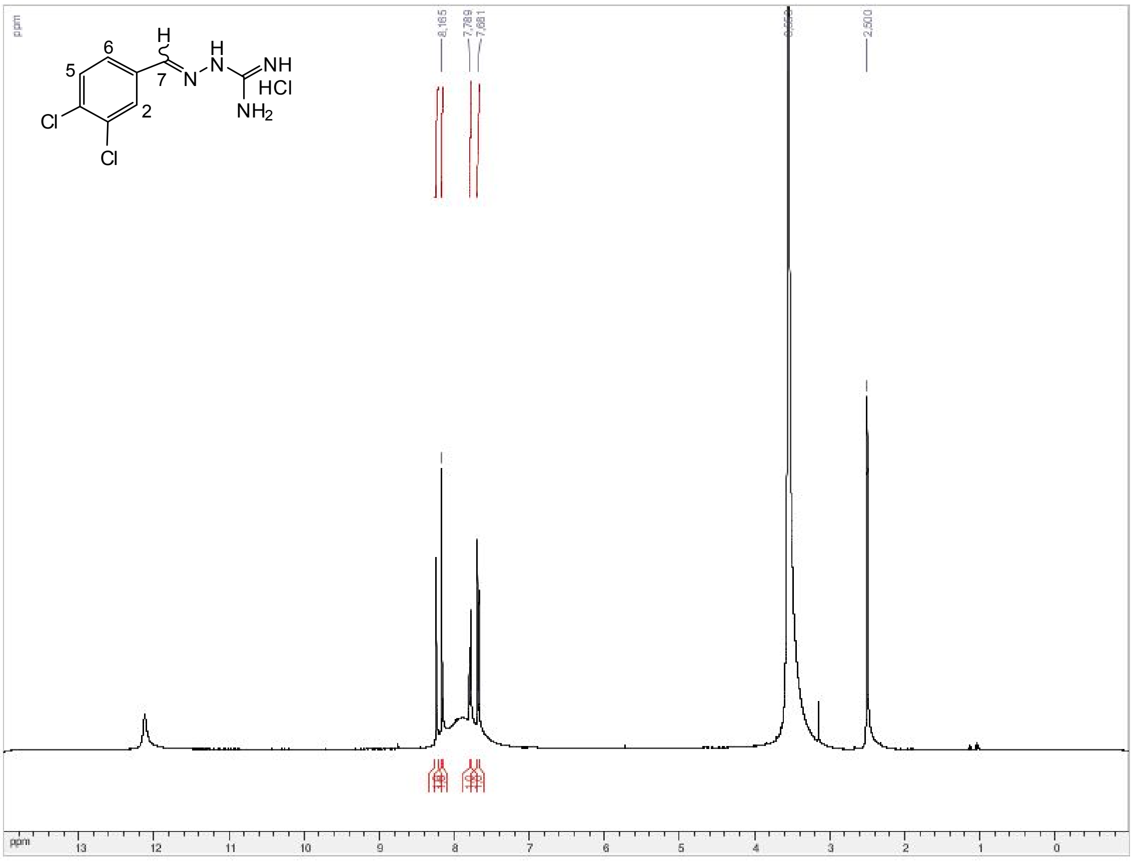

Figure S63. ^{1}H NMR (400Hz) in DMSO-d6 of LQM17.

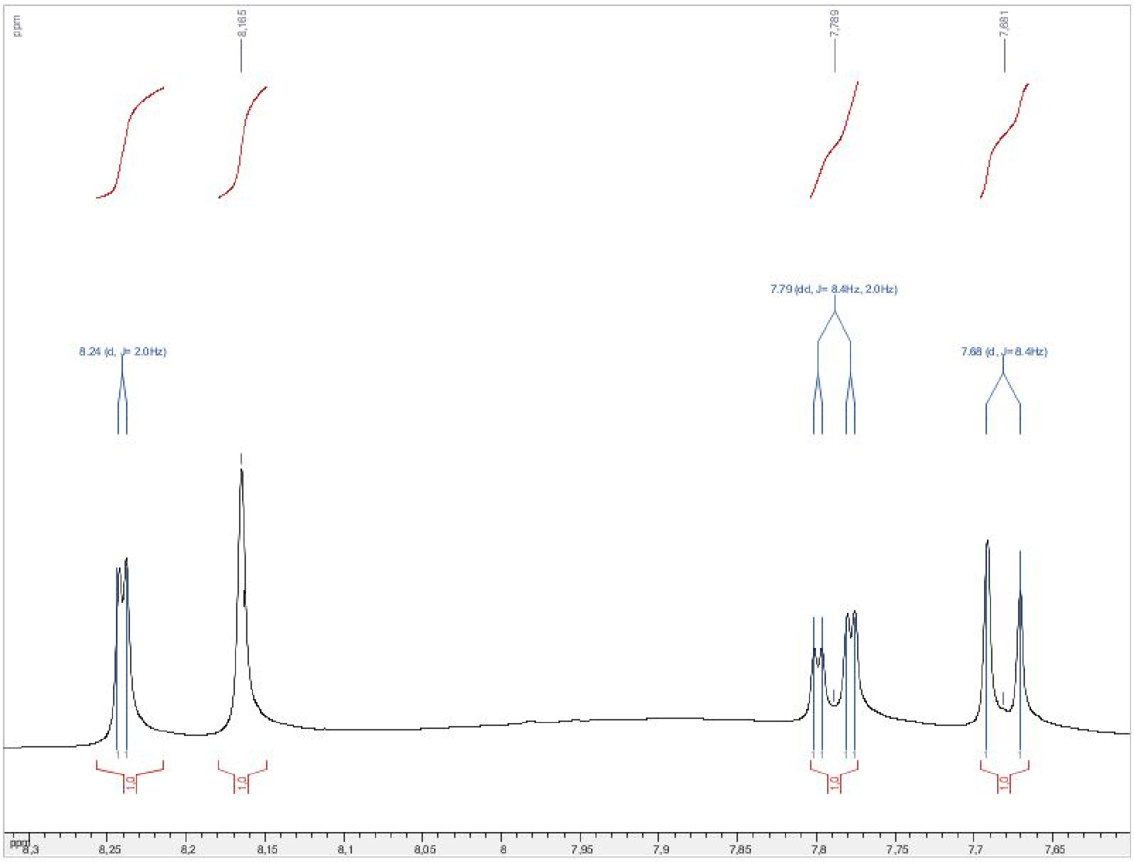

Figure S64. Magnification from δ7.0 to δ8.3.

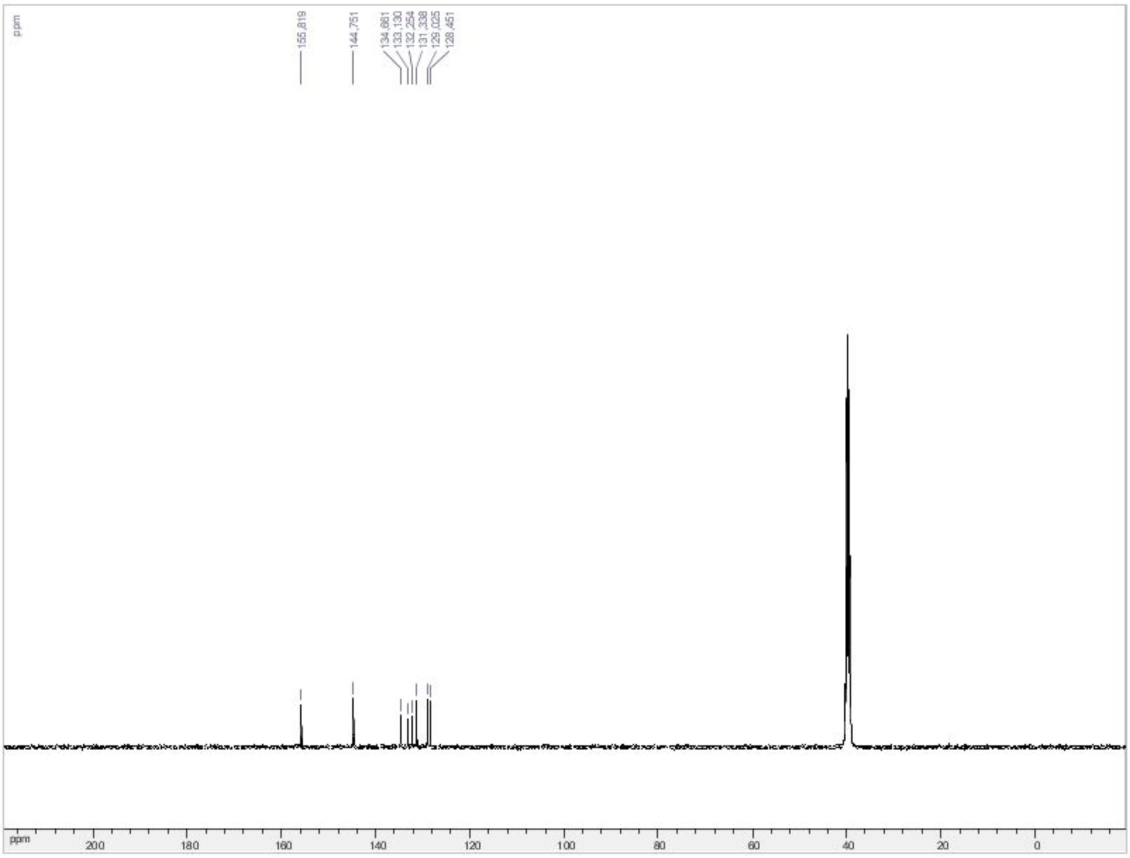

Figure S65. ^{13}C NMR (100Hz) in DMSO-*d6* of LQM17.

LQM18:

0.3015 g of substance 18 (1.2934 mmol) was obtained with a yield of 92.65%. **^{1}H NMR** (400 MHz, DMSO-d6): 7.47 (m, 2H); 7.74 (d, 1H, J=8.0); 8.06 (s, 1H); 8.17 (s, 1H). **^{13}C NMR** (100 MHz, DMSO-d6): 126.844; 127.302; 130.589; 131.043; 134.196; 136.05; 145.791; 155.815.

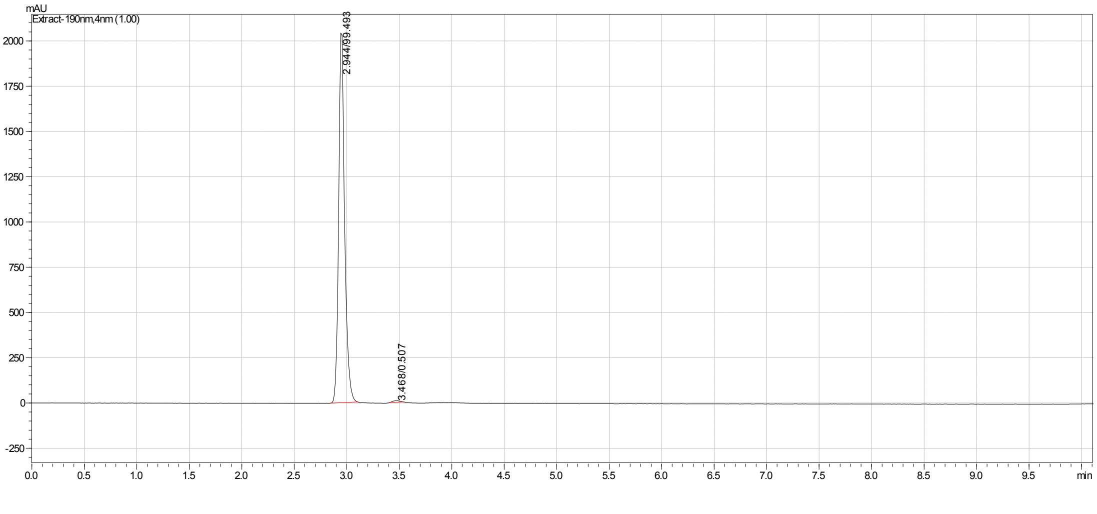

Figure S66. Chromatogram of substance LQM18

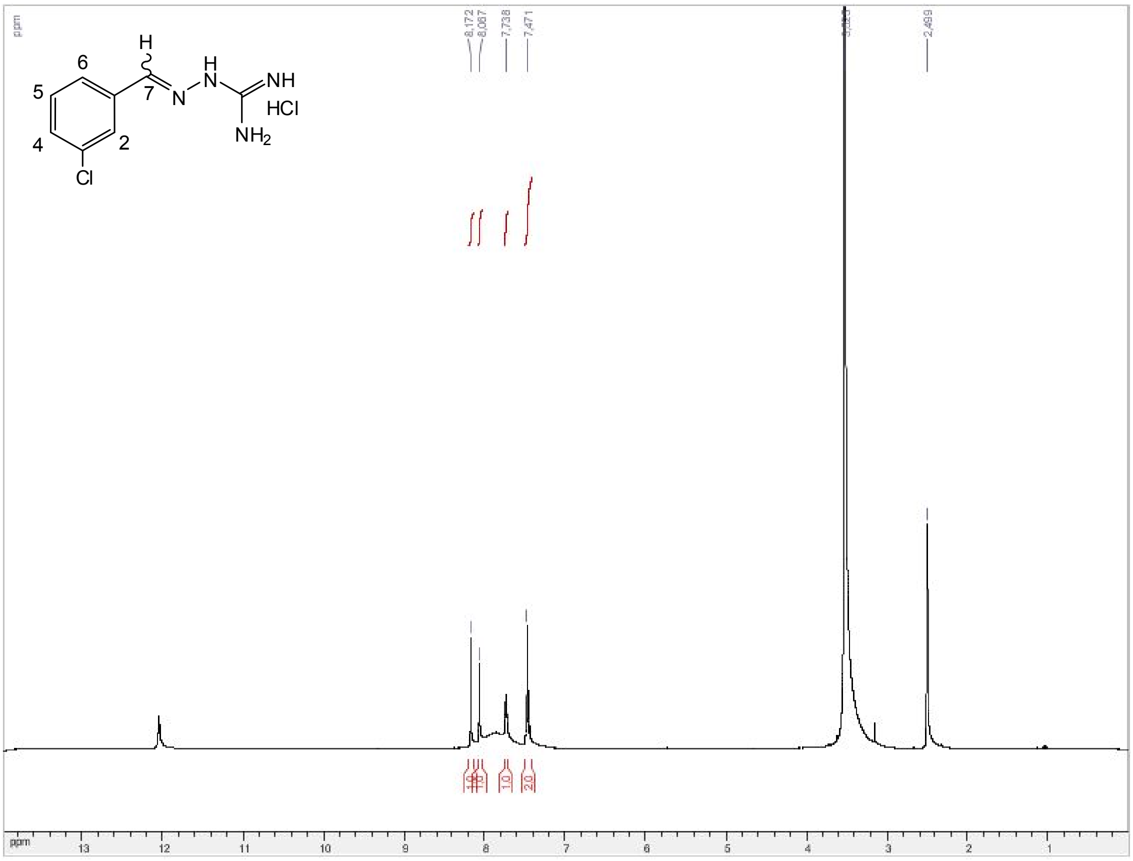

Figure S67. ^{1}H NMR (400Hz) spectrum in DMSO-*d6* of LQM18.

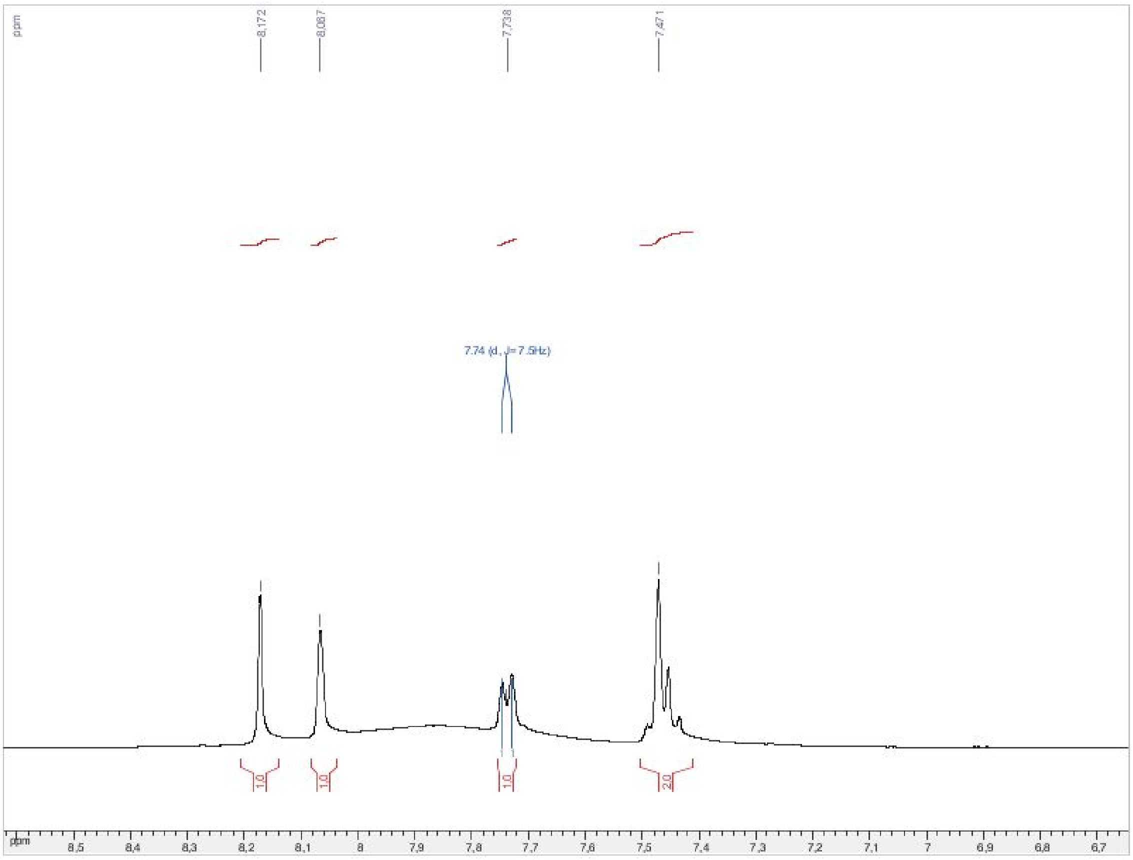

Figure S68. Magnification from δ6.7 to δ8.5.

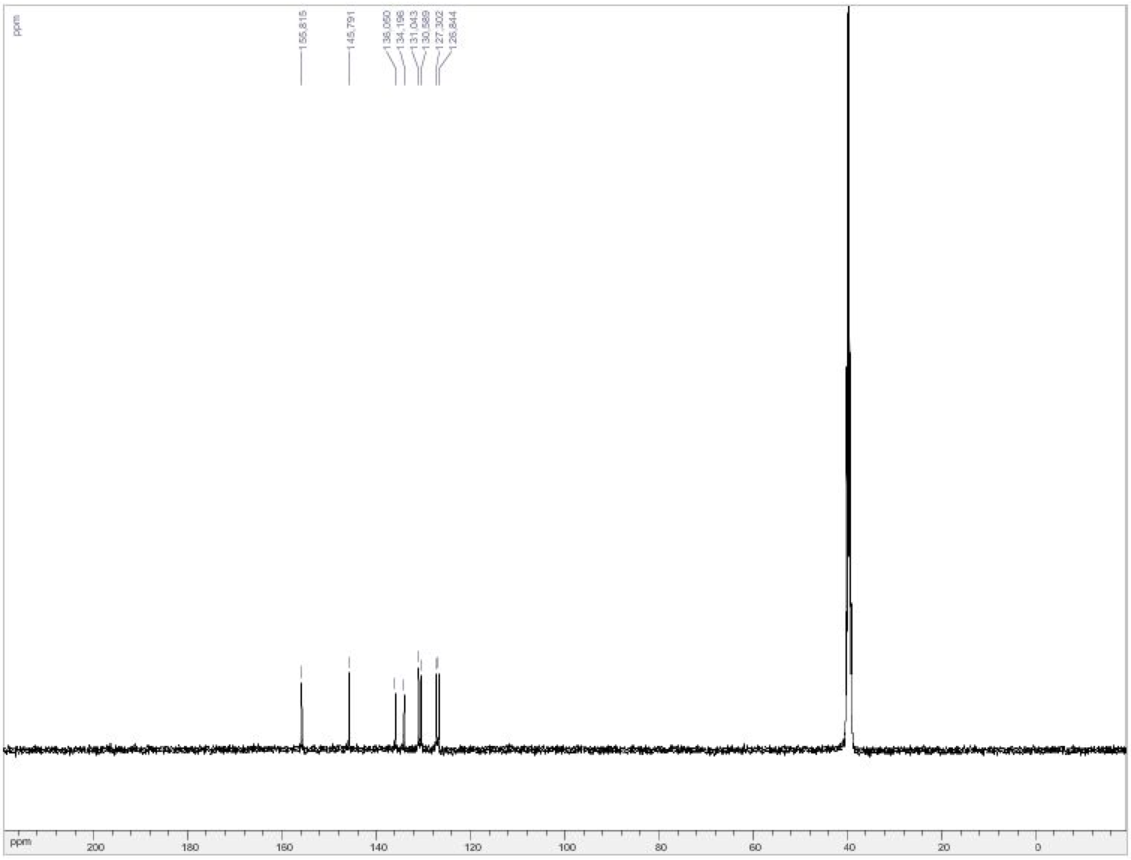

Figure S69. ^{13}C NMR (100Hz) in DMSO-*d6* of LQM18.

LQM19:

0.2761 g of substance 19 (1.0049 mmol) was obtained in 91.54% yield. **^{1}H NMR** (400 MHz, DMSO-d6): 7.36 (m, 5H); 7.49 (m, 4H); 8.06 (s, 1H). **^{13}C NMR** (100 MHz, DMSO-d6): 126.844; 127.302; 130.589; 131.043; 134.196; 136.05; 145.791; 155.815.

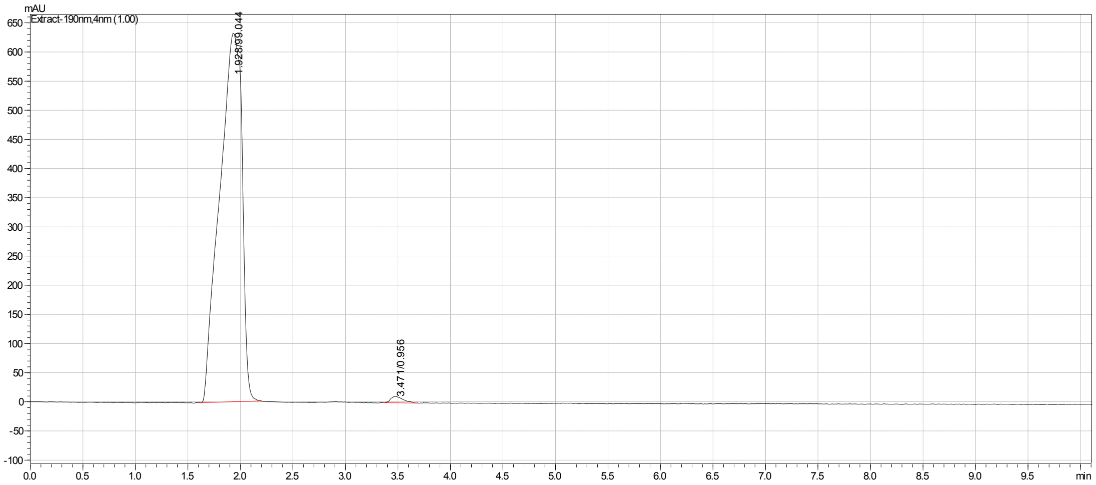

Figure S70. Chromatogram of substance LQM19

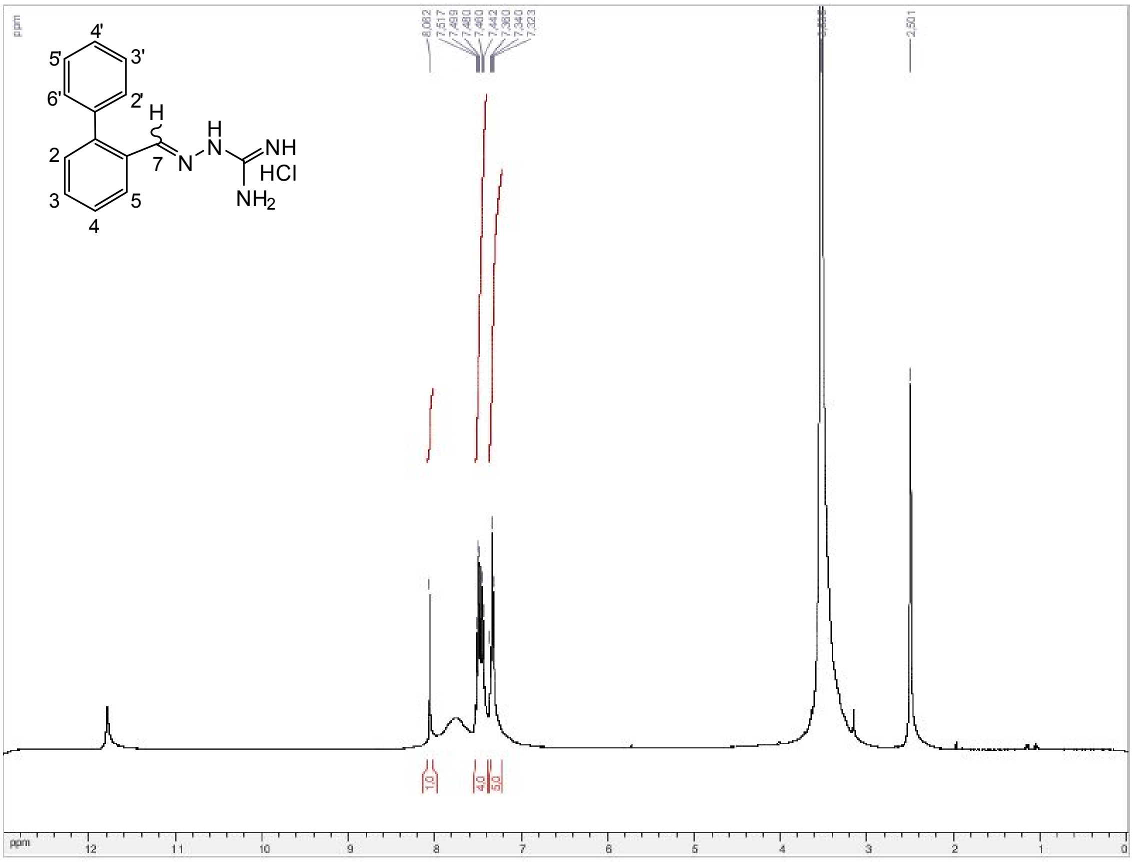

Figure S71. ^{1}H NMR (400Hz) spectrum in DMSO-*d6* of LQM19.

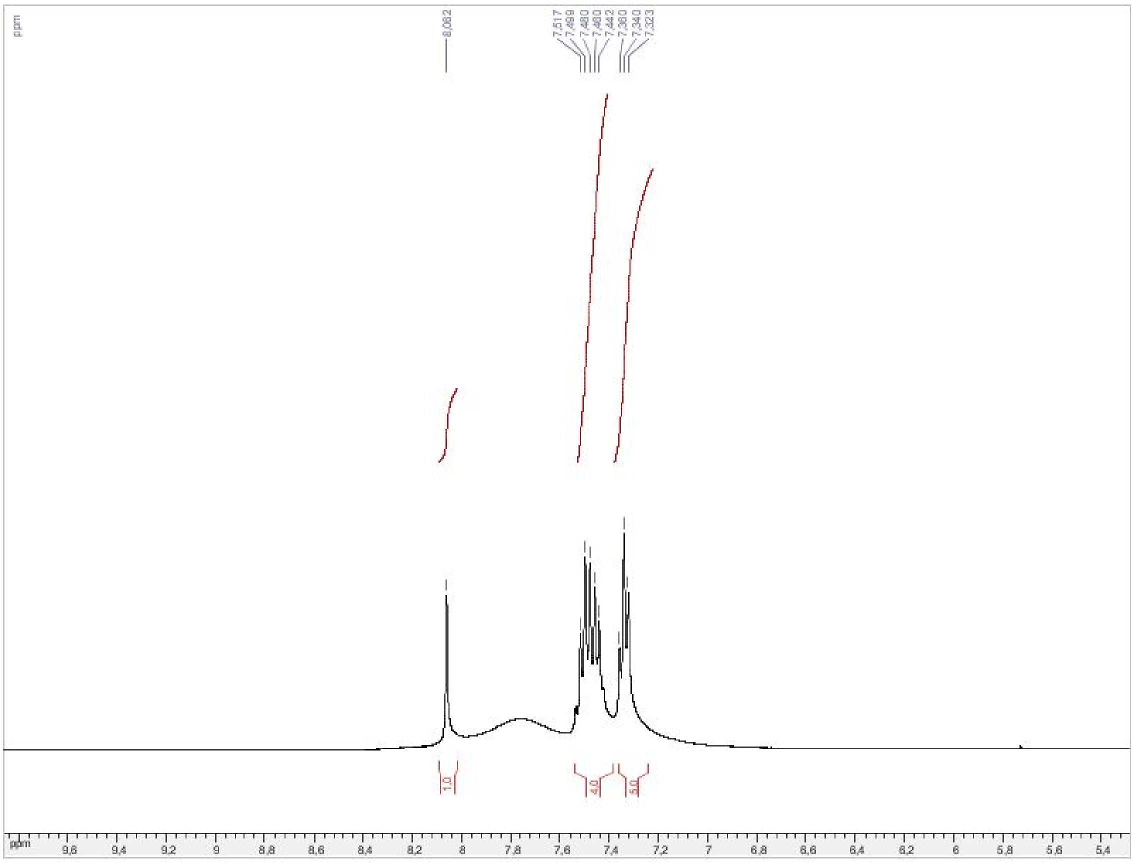

Figure S72. Magnification from δ5.4 to δ9.6.

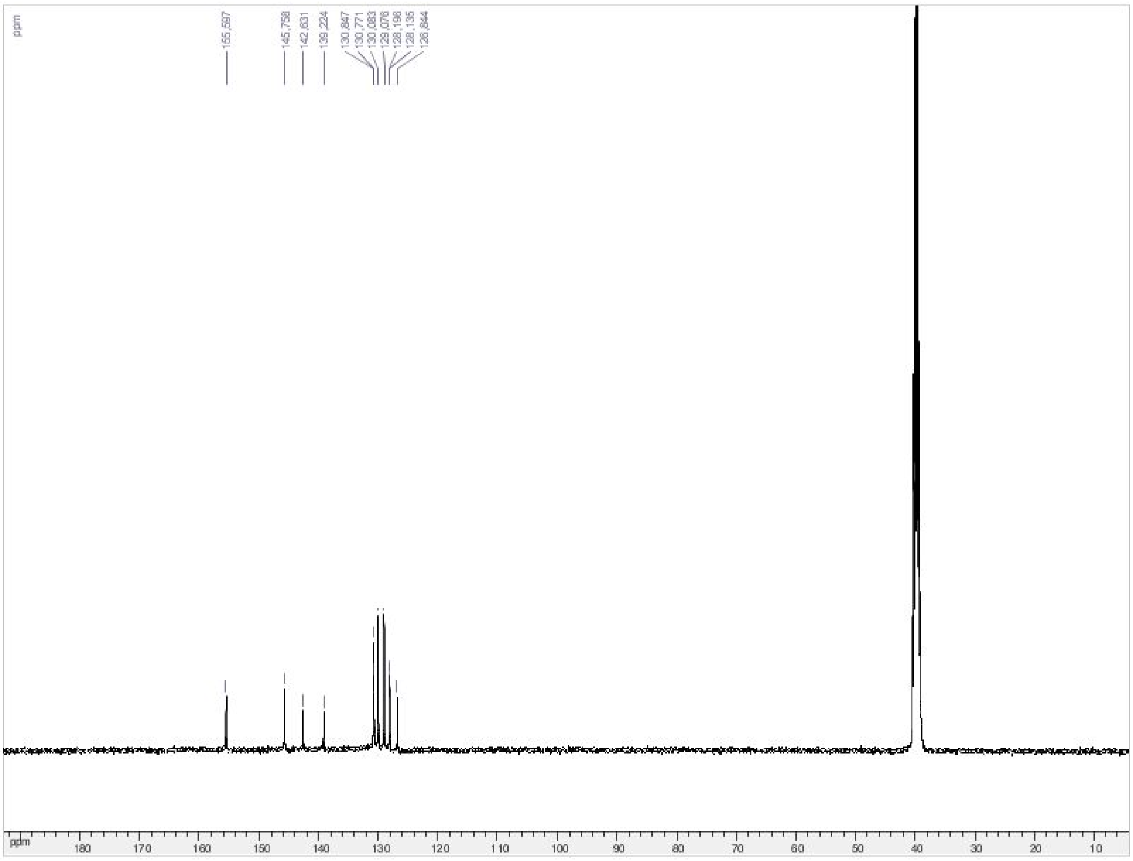

Figure S73. ^{13}C NMR (100Hz) in DMSO-d6 of LQM19.

LQM20:

0.2694 g of substance 20 (1.0104 mmol) was obtained with a yield of 87.96%. **^{1}H NMR** (400 MHz, DMSO-d6): 7.60 (t, 2H, J=7.8); 7.70 (d, 1H, J=7.8); 8.12 (d, 1H, J=7.8); 8.21 (s, 1H); 8.31 (s, 1H). **^{13}C NMR** (100 MHz, DMSO-d6): 124.171; 125.833; 127.157; 130.298; 132.203; 134.959; 145.929; 123.124; 155.746.

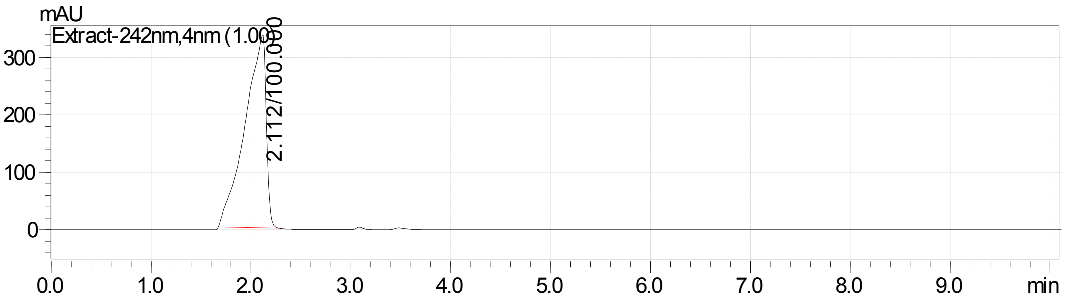

Figure S74. Chromatogram of substance LQM20

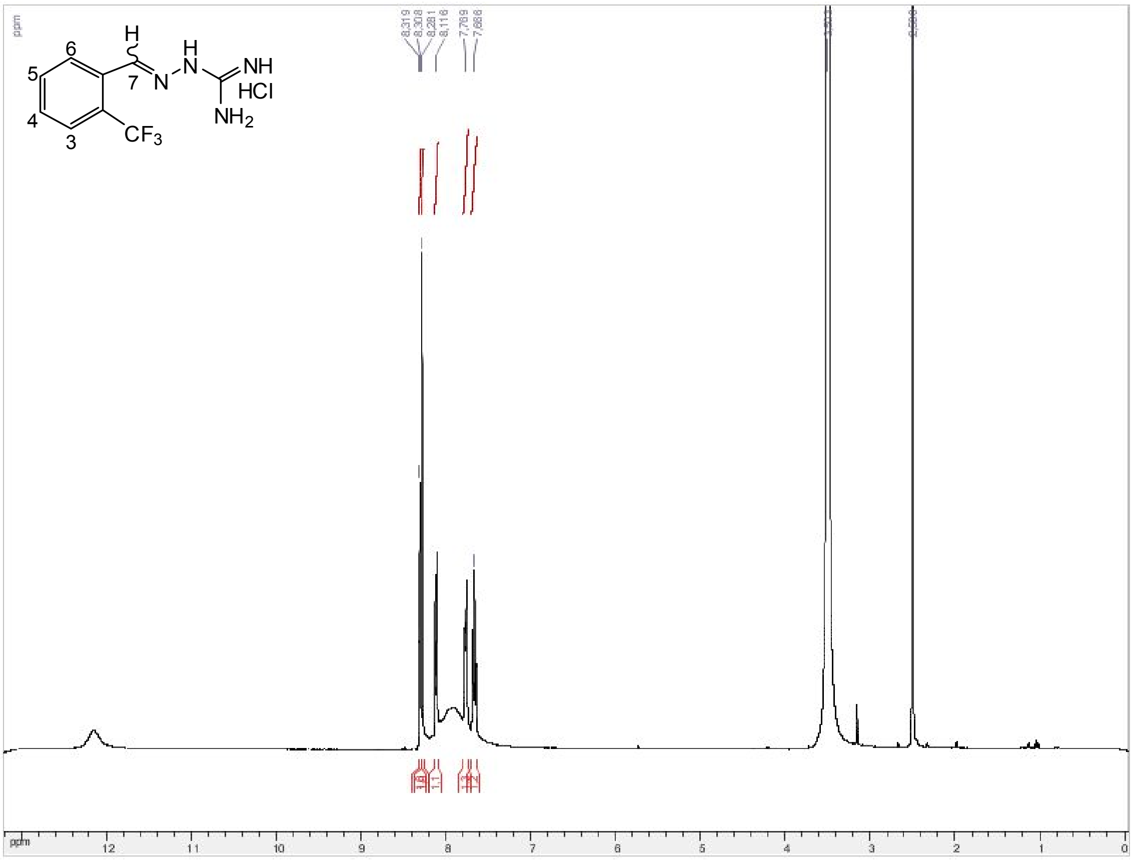

Figure S75. ^{1}H NMR (400Hz) spectrum in DMSO-*d6* of LQM20.

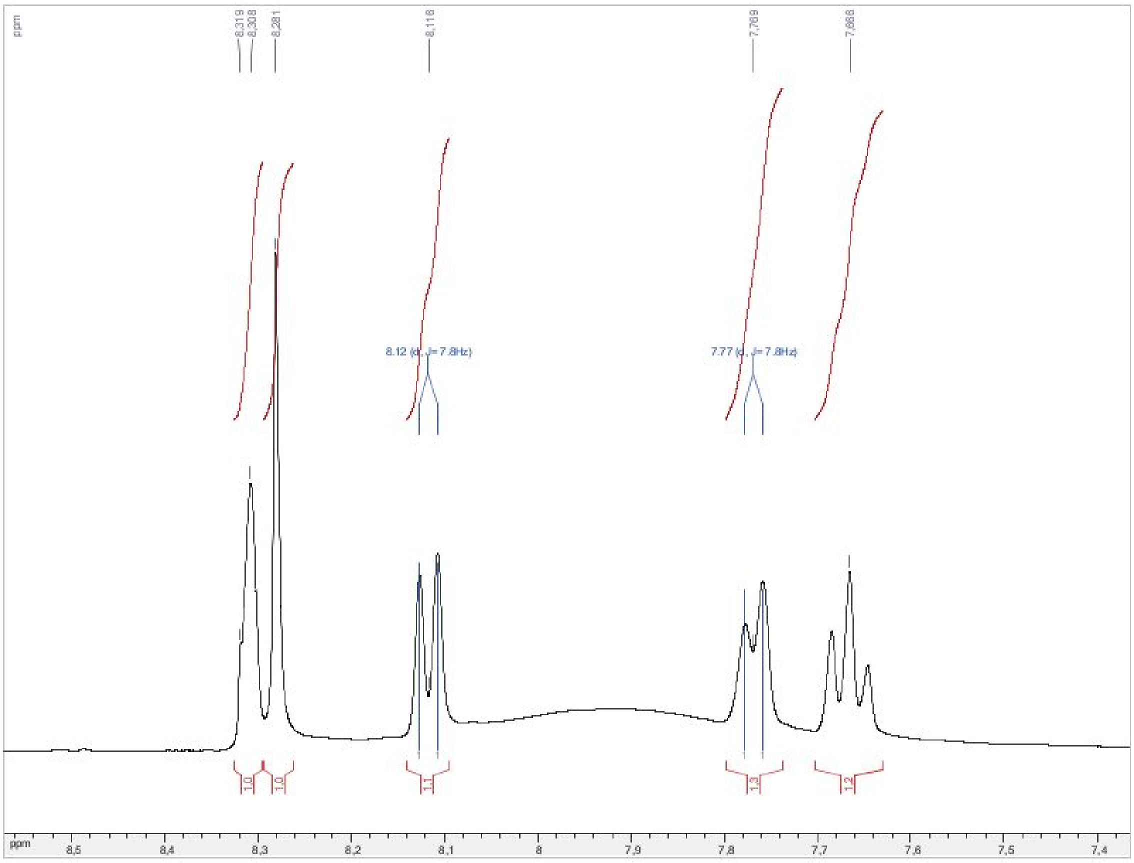

Figure S76. Magnification from δ7.4 to δ8.5.

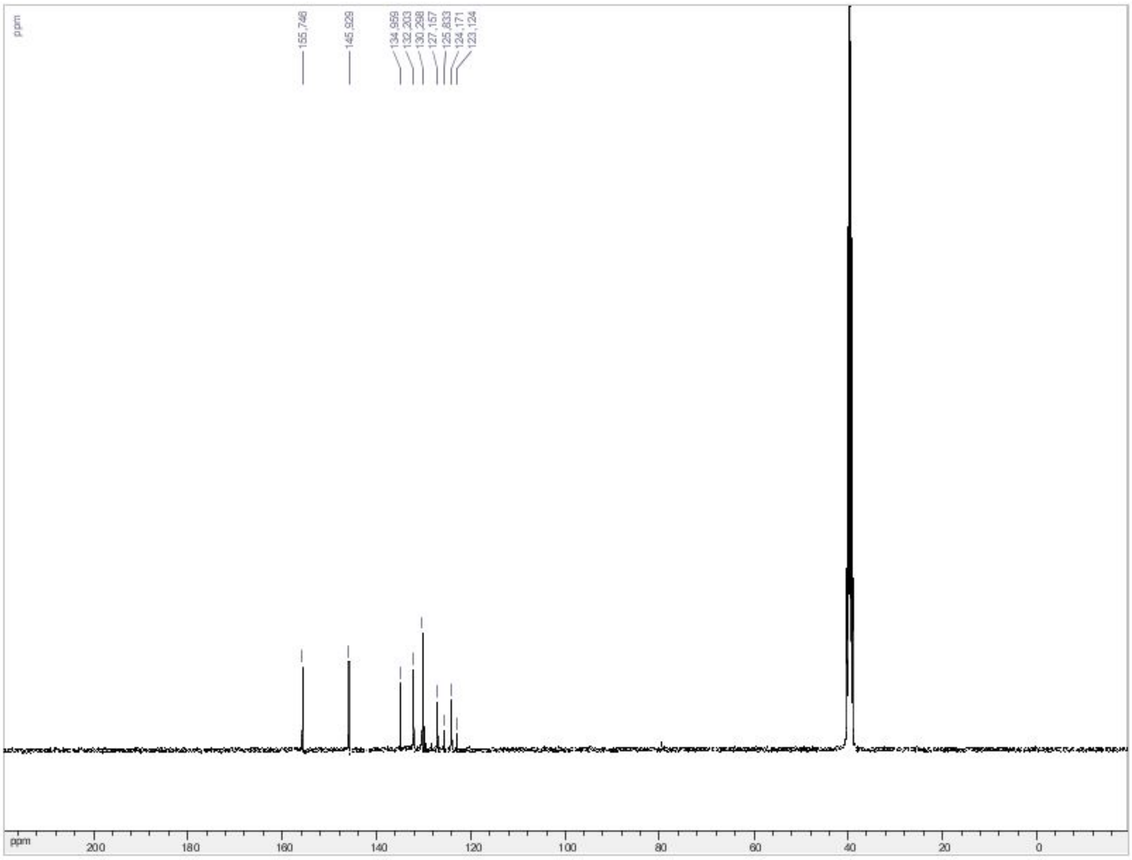

Figure S77. ^{13}C NMR (100Hz) in DMSO-d6 of LQM20.
